# Cytotoxicity of Atropisomeric [1,1′‐Binaphthalene]‐2,2′‐Diamines (BINAM) and Analogs in Human Cancer Cells: Enantioselectivity, Structure–Activity Relationships, and Mechanism

**DOI:** 10.1002/cmdc.202500426

**Published:** 2025-08-27

**Authors:** Malte Eichelbaum, Patrick J. Bednarski

**Affiliations:** ^1^ Pharmaceutical/Medicinal Chemistry Institute of Pharmacy University of Greifswald Friedrich‐Ludwig‐Jahn‐Str. 17 17489 Greifswald Germany

**Keywords:** atropisomers, biaryls, cancer, drug discovery, tubulin

## Abstract

Binaphthyls usually serve as key chiral ligands in catalysts for asymmetric syntheses, having been reported in thousands of published reactions. Herein, the discovery that atropisomeric (*R*)‐[1,1′‐binaphthalene]‐2,2′‐diamine (***R*‐BINAM, 1(*R*)**) is a moderately potent spindle poison, causing antiproliferation, depolymerization of microtubules, multipolar spindles, pericentriolar material (PCM) fragmentation, mitotic catastrophe, multinucleated cells, and apoptosis in cancer and normal human cell lines, is reported. Furthermore, the resulting abnormalities resemble those induced by microtubule‐depolymerizing agents (MDAs) such as colchicine. In contrast, the enantiomer ***S*‐BINAM** (**1(*S*)**) was inactive in all biological assays. Additionally, the structure–activity relationships of a selection of ***R*‐** and ***S*‐BINAM** derivatives with key structural differences have been studied; these studies show the same enantiomeric trend as with ***R*‐BINAM** and provide insight into the structural requirements for the antiproliferative activity of this compound class. These findings should be useful for the development of more selective spindle poisons, especially due to the natural rigidity of binaphthyls and their scaffold that allows for various modifications.

## Graphical abstract

It is reported for the first time that atropisomeric (*R*)‐[1,1^′^‐binaphthalene]‐2,2^′^‐diamine (*R*‐BINAM, 1(*R*)), but not (*S*)‐[1,1^′^‐binaphthalene]‐2,2^′^‐diamine (*S*‐BINAM, 1(*S*)), is a moderately potent spindle poison, causing antiproliferation, depolymerization of microtubules, multipolar spindles, pericentriolar material (PCM) fragmentation, mitotic catastrophe, multinucleated cells, and apoptosis in cancer and normal human cell lines.

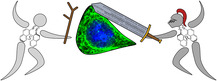

## 1 Introduction

**Figure 1:**
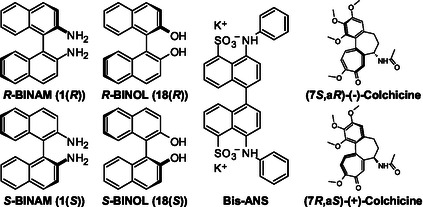
Structures of BINAM, BINOL, Bis‐ANS and colchicine enantiomers.

Atropisomers represent a subclass of axial chirality and are defined as isolatable rotamers due to their strongly sterically hindered rotation around a single bond. In the past, the pharmaceutical industry frequently avoided stable atropisomers and viewed rapidly isomerizing atropisomers as achiral.^[^ ^1^ ^]^ This overlook is now being challenged, as illustrated by the appearance of several recent reviews on atropisomers in medicinal chemistry.^[^ 1, 2, 3, 4 ^]^ Nonetheless, a good example of how this form of isomerism has been ignored in the past is the fact that the atropisomer colchicine is usually represented with no stereo bonds or only those that display the point chirality, although (7*R*,a*S*)‐(+)‐colchicine is about 40‐fold less cytotoxic than (7*S*,a*R*)‐(–)‐colchicine (**Figure** **1**).^[^ ^5^ ^,^ ^6^ ^]^ Due to their toxicity as anticancer drugs, there are currently no FDA‐approved colchicine binding site inhibitors (CBSIs). Furthermore, to our knowledge, there are no novel atropisomeric CBSIs outside structural homologs of colchicine, despite the fact that rigidity may reduce a molecule's pharmacological promiscuity and consequently, its toxicity.^[^ ^7^ ^]^

1,1′‐Binaphthyls can form particularly stable atropisomers; for instance, [1,1′‐binaphthalene]‐2,2′‐diol (BINOL) has a racemization half‐life at room temperature of about two million years.^[^ ^1^ ^]^ 1,1′‐Binaphthyls are frequently used in organic synthesis as reagents, especially as ligands for organometallic catalysts in enantioselective syntheses. Thousands of reactions are described in the literature that employ BINAM‐ and BINOL‐based metal catalysts.

Less has been reported on their biological properties, in particular their effects on living cells. The most prominent example is the dye 4,4′‐bis(phenylamino)‐[1,1′‐binaphthalene]‐5,5′‐disulfonic acid (Bis‐ANS), which is marketed as a hydrophobic surface marker due to its pronounced fluorescence that occurs only upon binding to nonpolar cavities in proteins.^[^ ^8^ ^]^ Additionally, cytotoxic (*R*)‐ and (*S*)‐[1,1′‐binaphthalene]‐2,2′‐diaminodichlorido‐Pt(II) complexes have been previously reported by our group^[^ ^9^ ^]^ as well as others.^[^ 10, 11, 12, 13 ^]^

During this work with the chiral BINAM‐platinum complexes, we observed that the free diamine ligands have enantioselective antiproliferative properties, with ***R*‐BINAM** (**1(*R*)**) acting as eutomer and ***S*‐BINAM** (**1(*S*)**) as distomer. These findings prompted us to further investigate the structure–activity relationships as well as mechanism of action of these interesting chiral ligands. The results of these studies are the topic of this publication.

## 2 Results and Discussion

### 2.1 Chemistry

**Scheme 1:**
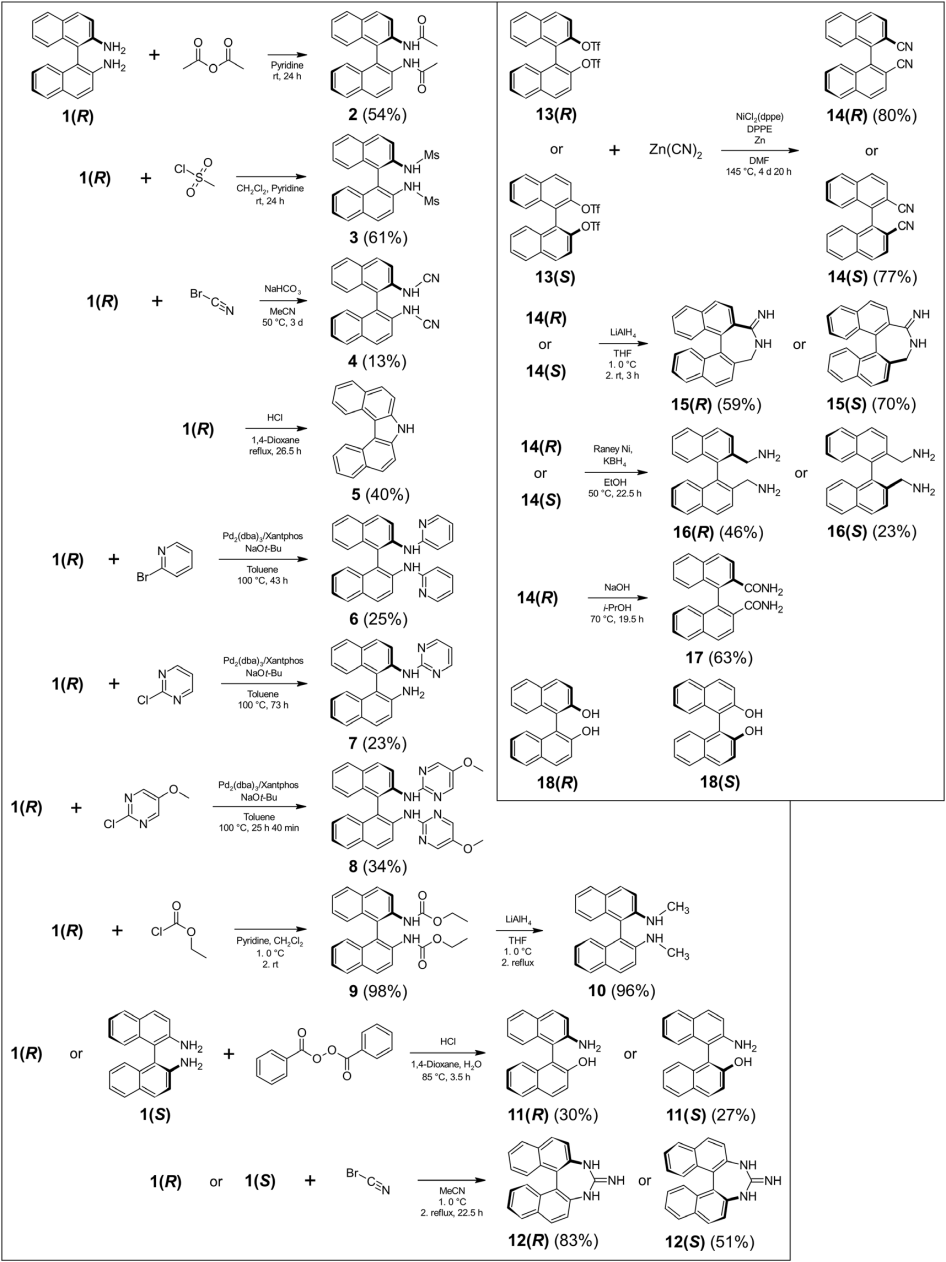
Synthetic routes of binaphthyls 2–17.

The synthetic routes to the analog binaphthyls together with their yields are shown in **Scheme** **1**. Reacting **1(*R*)** with acetic anhydride in pyridine afforded compound **2**. Compound **3** was prepared according to literature in a reaction of **1(*R*)** with mesyl chloride in pyridine and CH_2_Cl_2_.^[^ ^14^ ^]^ Heating **1(*R*)** at reflux in 1,4‐dioxane and HCl resulted in **5** by an acid‐catalyzed condensation.^[^ ^15^ ^]^ Buchwald‐Hartwig amination of **1(*R*)** and heteroaryl chlorides or bromides afforded compounds **6**, **7,** and **8**.^[^ ^16^ ^,^ ^17^ ^]^ Denmark et al. showed that **10** can be obtained by reacting **1(*R*)** with ethyl chloroformate and reducing the formed carbamate **9** with LiAlH_4_.^[^ ^18^ ^]^ Compounds **11(*R*)** and **11(*S*)** were obtained according to a literature method by reacting **1(*R*)** or **1(*S*)** with benzoyl peroxide in 1,4‐dioxane, water, and HCl. The authors proposed a radical Bucherer reaction as an explanation for this transformation.^[^ ^19^ ^]^ Compounds **12(*R*)** and **12(*S*)** were synthesized according to a literature method by reacting **1(*R*)** or **1(*S*)** in MeCN with cyanogen bromide under reflux.^[^ ^20^ ^]^ Compound **4** was obtained with the same reagents by limiting the reaction temperature to 50 °C. Compounds **14(*R*)** and **14(*S*)** were obtained via nickel catalyzed dicyanation of **13(*R*)** and **13(*S*)** with a slightly modified procedure [Zn(CN)_2_ instead of KCN] from Sato et al. In accordance with the same publication, **14(*R*)** and **14(*S*)** were reduced with Raney Nickel and KBH_4_ to obtain **16(*R*)** or **16(*S*)**.^[^ ^21^ ^]^ In contrast, reduction of the same compounds with LiAlH_4_ led to the cyclic amidines **15(*R*)** or **15(*S*)**; Sieveking et al. explained this behavior of dinitriles with the formation of an intermediate imine, that due to its negative charge is protected from another nucleophilic attack, but can act as a nucleophile itself and undergo a ring closure with the second nitrile.^[^ ^22^ ^]^ Partial hydrolysis of **14(*R*)** with NaOH in *i*‐PrOH afforded **17**.^[^ ^23^ ^]^

### 2.2 Antiproliferative Potencies and Structure‐Activity Relationships

Initial testing by the crystal violet antiproliferation assay^[^ ^24^ ^]^ at 10 µM for 96 h (**Table** **1**) showed that besides **1(*R*)**, **11(*R*)**, **15(*R*)**, **12(*R*)**, **12(*S*)**, **16(*R*)**, and **16(*S*)** are also active (percent growth < 40% in every cell line) against the four human cancer cell lines A2780 (ovarian), SISO (cervix), DAN‐G (pancreas), and LCLC‐103H (non‐small cell lung). As with **1(*S*)**, no antiproliferative activity with either **11(*S*)** or **15(*S*)** was observed. This proves that the enantioselective antiproliferative activity is not limited to **1(*R*)** and persists even when key structural changes are introduced. Furthermore, the pronounced activity of **15(*R*)** indicates that the substituents on 2 and 2′ come into close proximity within the target structure. Due to the observed chiral discrimination between *R*‐ and *S*‐stereoisomers, the target structure must also be chiral, most likely a protein or nucleic acid.

**Table 1:** Percent growth following a 96 h treatment with 10 µM of the respective binaphthyl analogs in four human cancer cell lines.

Compd	Percent growth ± SDa), b)			
	A2780	SISO	DAN‐G	LCLC‐103H
1(*R*)	−0.29 ± 0.69	−2.22 ± 0.74	−0.82 ± 2.81	−1.78 ± 1.15
1(*S*)	87.24 ± 6.92	84.63 ± 10.28	80.37 ± 8.70	88.35 ± 4.62
2	101.05 ± 21.35	102.09 ± 3.42	92.53 ± 15.20	92.99 ± 21.56
3	106.56 ± 3.24	102.96 ± 6.25	100.53 ± 4.22	107.00 ± 9.32
4	21.48 ± 3.82	67.86 ± 4.00	88.99 ± 8.55	82.79 ± 13.73
5	79.04 ± 7.56	68.10 ± 2.47	93.30 ± 3.84	80.25 ± 7.76
6	Insoluble			
7	55.99 ± 4.04	81.14 ± 3.83	88.23 ± 1.30	73.97 ± 3.03
8	48.99 ± 12.48	80.81 ± 8.33	66.31 ± 9.85	83.74 ± 15.62
9	32.34 ± 1.83	87.72 ± 1.82	59.51 ± 4.60	49.71 ± 9.89
10	91.35 ± 12.76	102.51 ± 9.15	92.66 ± 16.49	97.53 ± 9.70
11(*R*)	−0.31 ± 0.51	0.13 ± 1.62	−4.61 ± 0.79	0.00067 ± 0.37
11(*S*)	79.68 ± 12.79	85.24 ± 10.11	73.10 ± 7.25	83.14 ± 6.16
12(*R*)	−0.26 ± 0.25	−1.15 ± 0.88	−3.34 ± 1.19	−0.66 ± 0.70
12(*S*)	−0.38 ± 1.14	−0.34 ± 2.31	−2.17 ± 0.73	−0.57 ± 0.93
13(*R*)	70.80 ± 7.68	91.31 ± 5.58	66.56 ± 3.52	68.65 ± 8.41
13(*S*)	68.47 ± 8.55	99.90 ± 6.04	68.65 ± 6.16	63.34 ± 4.90
14(*R*)	Insoluble			
14(*S*)	Insoluble			
15(*R*)	0.78 ± 2.53	−1.73 ± 0.83	−4.07 ± 1.53	−1.31 ± 0.63
15(*S*)	59.52 ± 6.21	69.96 ± 14.50	84.45 ± 3.27	73.17 ± 9.67
16(*R*)	−0.71 ± 1.21	−2.79 ± 0.65	−2.12 ± 1.42	−2.30 ± 0.37
16(*S*)	−0.65 ± 0.72	−2.29 ± 0.87	−2.42 ± 1.18	−2.67 ± 0.44
17	94.02 ± 10.93	108.59 ± 7.72	104.76 ± 11.27	102.84 ± 14.96
18(*R*)	76.94 ± 5.03	94.30 ± 9.30	8.10 ± 6.55	28.10 ± 12.83
18(*S*)	65.98 ± 9.69	76.07 ± 6.68	67.47 ± 3.75	56.19 ± 14.97

a) Growth measured by the crystal violet staining method.

b) Mean of three or more independent experiments.

Interestingly, the cyclic guanidines **12(*R*)** and **12(*S*)** both exhibited a comparable high activity against all tested cancer cell lines. We hypothesize that the ring closure caused the angle between the two naphthyl rings to be reduced and constrained, making the structures of both enantiomers similar and no longer chirally distinguishable by the target structure. The dihedral angles between positions 2 and 2′ of the active binaphthyl *R*‐enantiomers were determined by using PyMOL^[^ ^25^ ^]^ after UFF optimization in Avogadro.^[^ ^26^ ^]^ Indeed, **12(*R*)** showed a remarkably smaller dihedral angle (24.3°) compared to **1(*R*)** (66.1°), **11(*R*)** (62.7°), and **15(*R*)** (50.5°) (Figure S1, Supporting Information).

Due to their chemical instability at room temperature, which may have led to the formation of side products during the course of treatment, we did not calculate the GI_50_ values of **16(*R*)** and **16(*S*)**, despite the fact that we observed antiproliferation following treatment.

Interestingly, in the cell line A2780, compounds **4** and **9** did maintain a moderate antiproliferative activity at 10 µM. Carbamates are well‐known prodrug motifs and reports of cyanamide prodrugs (which were studied as alcohol deterrents) and their bioactivation were published as well.^[^ ^27^ ^,^ ^28^ ^]^ Therefore, the amines of **1(*R*)** might be easily formed inside cells after cleavage of the carbamate, making these possible prodrugs for **1(*R*)**. This argument is supported by the esterase activity of the A2780 cell line, which is similar to that of the liver cancer cell line HEPG2.^[^ ^29^ ^]^

Due to their prominence in anticancer agents, pyridines and pyrimidines substituents were introduced.^[^ ^30^ ^]^ In case of **6** this caused a decrease in aqueous solubility, that prohibited the determination of the antiproliferative activity at 10 µM. Compounds **7** and **8** were better water soluble and could be tested in cell culture, but they only showed low antiproliferative activity at 10 µM.

Remarkably, even small steric hindrances like a methyl group in **10** were able to negate the antiproliferative activity, which explains the low potency of binaphthyls with bulky substituents.

The carbazole compound **5** showed only weak antiproliferative activity, which suggests that the two naphthyl rings must be tilled out of the plane of each other for the compounds to be active.

Sufficiently active compounds were subsequently put through a secondary screening scheme in order to determine their 50‐percent growth‐inhibitory (GI_50_) values (**Table** **2**). With one exception (**18(*R*)**), all showed antiproliferative activity with GI_50_ values below 4 µM in five human cancer cell lines: A2780, SISO, DAN‐G, LCLC‐103H, and MCF‐7 (breast). In addition, a nontransformed human cell line, the 16HBE14o‐ (lung), was also sensitive to **1(*R*)**. The LCLC‐103H cell line appeared to be the most sensitive to **1(*R*)** and its analogs but there was little selectivity between the cell lines.

**Table 2:** Fifty‐percent growth‐inhibitory (GI_50_) values of binaphthyl analogs in five human cancer cell lines and one nontransformed lung cell line (16HBE14o‐) following a 96 h exposure.

Compd	GI_50_ a) [µM] (95% CI)					
	A2780	SISO	DAN‐G	LCLC‐103H	MCF‐7	16HBE14o‐
1(*R*)	1.80 (1.03–2.57)	1.99 (1.51–2.47)	2.00 (1.95–2.05)	1.27 (0.85–1.69)	1.33 (1.24–1.42)	1.13 (1.08–1.18)
11(*R*)	3.50 (2.08–4.91)	4.14 (1.89–6.38)	3.26 (2.80–3.72)	2.44 (1.74–3.14)	N/A	N/A
12(*R*)	1.48 (1.33–1.63)	2.40 (2.21–2.58)	2.60 (2.18–3.01)	1.11 (0.99–1.22)	N/A	N/A
12(*S*)	2.91 (2.47–3.35)	2.11 (1.56–2.66)	2.67 (2.43–2.90)	2.14 (1.95–2.32)	N/A	N/A
15(*R*)	2.36 (0.98–3.73)	3.06 (2.73–3.38)	4.04 (2.76–5.31)	2.33 (1.02–3.63)	N/A	N/A
18(*R*)	11.09 (10.69–11.50)	13.30 (11.15–15.44)	5.93 (3.60–8.26)	7.61 (3.20–12.02)	N/A	N/A

a) Mean of three or more independent experiments.

Interestingly, the antiproliferative activity in comparison to **1(*R*)** was decreased approximately by a factor of two when one amine was replaced by a phenol (**11(*R*)**) and by a factor of three to six when both were replaced by phenols (**18(*R*)**). BINAM bears two aromatic amines and thus is able to act as hydrogen‐bond donor (HBD). However, because of the resonance of the amines with the aromatic rings, BINAM is incapable of functioning as a hydrogen‐bond acceptor (HBA). Therefore, the superiority of **1(*R*)** over **11(*R*)** and **18(*R*)** suggests that the potential to function as an HBA will not enhance the potency and a weakened HBD capacity will diminish potency. A further explanation could be that oxygen bearing functional groups lead to inter‐ and intramolecular hydrogen bonds, which would also explain why a compound like **17** is completely inactive.

Additionally, we assessed the acute toxicity of the sufficiently active compounds and their enantiomers by the crystal violet antiproliferation assay after treatment at 10 µM for 24 h (**Table** **3**). All active compounds exhibited noticeable growth inhibition or even cytotoxicity after just 24 h, whereas their inactive enantiomers showed only marginal or no acute effects. Notably, **12(*R*)** and **12(*S*)** displayed high cytotoxicity in all cell lines, which could be attributed to a faster effect due to their low dihedral angle.

**Table 3:** Percent growth following a 24 h treatment with 10 µM of the respective binaphthyl analogs in four human cancer cell lines.

Compd	Percent Growth ± SDa), b)			
	A2780	SISO	DAN‐G	LCLC‐103H
1(*R*)	−3.55 ± 0.88	−38.25 ± 6.04	4.37 ± 7.27	−25.51 ± 15.52
1(*S*)	73.88 ± 6.98	88.60 ± 5.16	87.39 ± 6.64	70.01 ± 10.95
11(*R*)	8.67 ± 4.93	−31.82 ± 9.96	−1.78 ± 13.23	−5.46 ± 12.82
11(S)	89.19 ± 3.24	78.68 ± 15.28	82.23 ± 9.50	82.12 ± 8.61
12(*R*)	−21.64 ± 1.12	−47.54 ± 4.21	−42.86 ± 11.50	−44.42 ± 18.44
12(*S*)	−16.19 ± 2.26	−43.16 ± 1.48	−38.95 ± 10.93	−36.81 ± 16.06
15(*R*)	15.73 ± 4.08	−1.44 ± 5.06	31.14 ± 9.23	46.45 ± 25.08
15(*S*)	91.27 ± 5.18	87.05 ± 21.81	97.29 ± 12.36	95.06 ± 7.61
18(*R*)	93.90 ± 16.81	89.08 ± 16.61	84.50 ± 6.88	109.92 ± 11.83
18(*S*)	107.34 ± 10.67	104.12 ± 22.17	98.34 ± 7.71	119.15 ± 18.19

a) Growth measured by the crystal violet staining method.

b) Mean of three independent experiments.

For further biological studies to identify the mechanism of action, only **1(*R*)** and **1(*S*)** were investigated because **1(*R*)** was the most potent of the compounds and **1(*S*)** was its inactive enantiomer.

### 2.3 Compound 1(*R*) Causes G2/M Arrest, Mitotic Catastrophe, and Apoptosis

**Figure 2:**
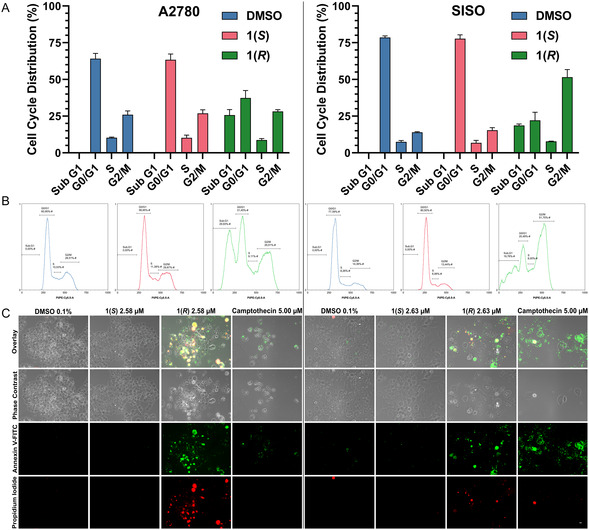
Compound **1(*R*)** causes G2/M arrest and apoptosis. A) Mean percentage (*n* = 3) of cells in each cell cycle phase following a 24 h treatment with the GI_90_ of **1(*R*)** (A2780: 2.58 µM, SISO: 2.63 µM), the same concentration of **1(*S*)** or DMSO (0.1%). B) Representative cell cycle histograms of each treatment condition. C) Annexin V‐FITC (green) and PI (red) staining is positive in A2780 and SISO cells after 48 h of treatment with the GI_90_ of **1(*R*)**, indicating early and late apoptosis. The same concentration of **1(*S*)** shows no difference to the DMSO solvent control. The scale bar (10 µm) is valid for all images.

Treatment of A2780 and SISO cancer cells at the GI_90_ of **1(*R*)** for 24 h and subsequent flow cytometric cell cycle analysis (**Figure** **2**A,B) showed a remarkable decrease of cells in G0/G1 and emergence of a Sub‐G1 population. The latter is a characteristic sign for DNA fragmentation that frequently occurs during apoptotic cell death.^[^ ^31^ ^]^ Furthermore, SISO cells showed a sharp rise in the G2/M population, indicating that **1(*R*)** is able to force cells into a G2/M arrest. On the other hand, a 24 h treatment with the same concentration of **1(*S*)** displayed no differences to the DMSO solvent control in both cell lines. This is consistent with the enantioselective effects of **1(*R*)** and suggests a cell cycle specific mechanism of action.

Utilizing the same cell lines and concentrations, we stained cells with Annexin V‐FITC and the fluorescent dye propidium iodide (PI) after a 48 h treatment (Figure 2C). Camptothecin (5.00 µM) was used as positive control. Annexin V is capable of binding phosphatidylserine, which is translocated from the inner to the outer side of the membrane during early apoptosis, and then detectable as fluorescence signal due to the conjugation with FITC (displayed in green). PI (displayed in red) cannot penetrate the membranes of viable cells but can in late apoptotic cells.^[^ ^32^ ^]^ Both overlaid dyes are visualized as yellow. After treatment with **1(*R*)**, both cell lines showed remarkable morphological abnormalities (multinucleation, macronucleation, micronucleation), which indicated that the cells underwent mitotic catastrophe. The latter is defined as an onco‐suppressive mechanism that occurs after abnormal mitosis and can be exogenously activated by various substances, including microtubule‐targeting agents (MTAs).^[^ ^33^ ^]^ Additionally, the cells exhibited a positive Annexin V staining in the outer cell membrane. The PI particularly stained small fragments and nonadherent cells. The camptothecin treatment showed a comparable Annexin V pattern to **1(*R*)**, despite the absence of multinucleated cells and a more pronounced reduction in cell count. **1(*S*)** once more displayed no distinction from DMSO. In summary, these findings indicate that **1(*R*)** triggers abnormal mitosis, leading to mitotic catastrophe, which subsequently results in nonviable multinucleated cells that ultimately undergo apoptosis.

### 2.4 Compound 1(*R*) Causes Malformed Spindles

**Figure 3:**
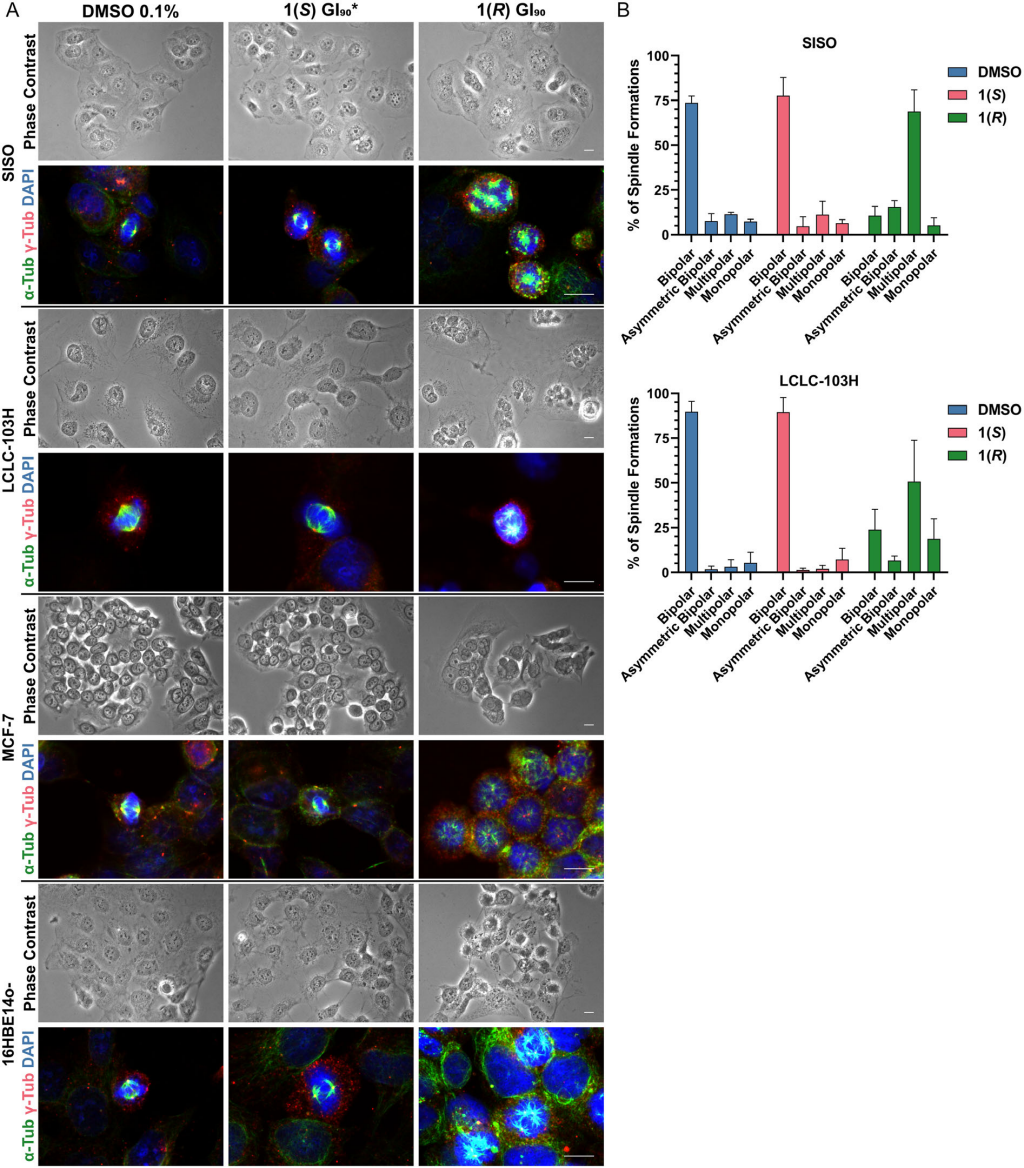
Compound **1(*R*)** induces the formation of multinucleated cells and multipolar spindles in both normal and cancerous human cell lines. A) Following 24 h of treatment at its GI_90_, **1(*R*)** promotes the development of multinucleated cells and multipolar spindles in diverse human cancer and normal cell lines; at the same concentration, **1(*S*)** does not exhibit differences to DMSO. *GI_90_ of **1(*R*)**. Alpha‐tubulin in green, gamma‐tubulin in red, nuclear DNA in blue. The scale bar (10 µm) is valid for the respective row of images. B) During the experiment outlined in section “A”, at least 50 mitotic SISO or LCLC‐103H cells per treatment condition were categorized based on their spindle morphology (*n* = 3, ± SD), showing that **1(*R*)** induces spindle abnormalities.

We further investigated the cell cycle and morphological aberrations with immunostaining of alpha‐tubulin and gamma‐tubulin coupled to fluorescence microscopy (**Figure** **3**A). After a 24 h treatment with the GI_90_ of **1(*R*)**, almost all SISO (GI_90_: 2.63 µM) and LCLC‐103H (GI_90_: 1.87 µM) cells were multinucleated. In addition, most mitotic cells treated with **1(*R*)** exhibited malformed spindles (monopolar, asymmetric bipolar, multipolar), with chromosomes failing to organize at the metaphase plate and instead being scattered across the cell (Figure 3B). In contrast, cells treated with the same concentration of **1(*S*)** showed no differences to those cells treated only with DMSO. These findings provide an additional puzzle piece, as multipolar cell divisions typically result in mitotic catastrophe.^[^ ^34^ ^]^ In subsequent experiments, we observed the same outcomes using the normal bronchial epithelial cell line 16HBE14o‐ (GI_90_ of **1(*R*)**: 1.66 µM) and the breast cancer cell line MCF‐7 (GI_90_ of **1(*R*)**: 2.00 µM), as discussed later.

### 2.5 Compound 1(*R*) Treatment Results in Cellular Microtubule Depolymerization

**Figure 4:**
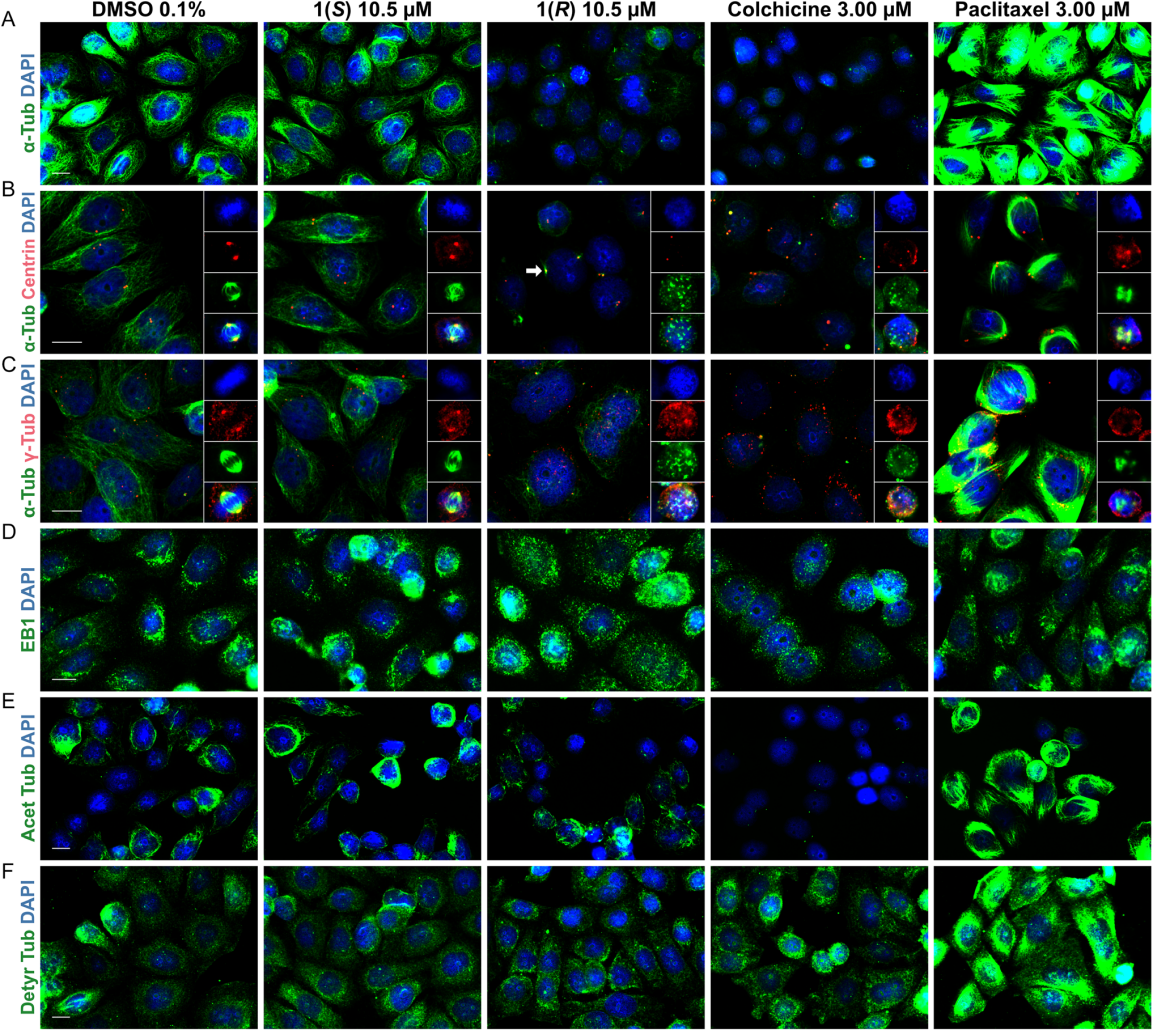
Similar to colchicine, **1(*R*)** depolymerizes cellular microtubules, exhibits signs of PCM fragmentation, scatters EB1, and has a minor effect on detyrosinated microtubules; **1(*S*)** did not show differences to the DMSO solvent control. SISO cells were subjected to treatment for 8 h, followed by staining with various antibodies and DAPI. Nuclear DNA is displayed in blue in all images. The scale bar (10 µm) is valid for the respective row of images. A) Alpha‐tubulin staining (green) indicates a reduced microtubule skeleton following **1(*R*)** treatment. B) **1(*R*)**‐treated cells do not show signs of centrosome amplification, but form multiple small tubulin fragments instead of bipolar spindles; alpha‐tubulin (green) is concentrated at the centrioles/centrosomes (red) after **1(*R*)** treatment (white arrow), indicating a high amount of non‐polymerized tubulin. C) **1(*R*)**‐treated cells exhibit evidence of PCM fragmentation, as demonstrated by the overlapping of gamma‐tubulin (red) fragments with alpha‐tubulin (green) fragments. D) **1(*R*)** induces a comparable scattering of EB1 (green) as colchicine. E) **1(*R*)** has no effects on acetylated microtubules (green). F) **1(*R*)** and colchicine induce a comparable minor decrease in detyrosinated microtubules (green).

We then aimed to amplify the observed effects with a higher concentration of **1(*R*)** before the cells complete a cell cycle; for this, SISO cells were treated with DMSO (0.1%), **1(*R*)** (10.5 µM, 4 x GI_90_), **1(*S*)** (10.5 µM), paclitaxel (3.00 µM) or colchicine (3.00 µM) for 8 h. While DMSO‐ and **1(*S*)**‐treated interphasic cells displayed no difference to the previous described 24 h experiment, **1(*R*)** caused an extreme malformed and perforated microtubule cytoskeleton, indicating a depolymerization of microtubules (**Figure** **4**A).

Moreover, the mitotic cells treated with 10.5 µM of **1(*R*)** no longer formed spindles; instead, they displayed numerous small microtubule fragments of varying sizes scattered throughout the cell (Figure 4B,C). We observed a similar morphology in mitotic SISO cells treated with 3.00 µM colchicine; this has been both shown and described for colchicine as well as other microtubule‐depolymerizing agents (MDAs), such as combretastatin A4, nocodazole, avanbulin, vinblastine, and the recently discovered gatorbulin‐1.^[^ 35, 36, 37, 38, 39, 40, 41, 42 ^]^ Consistent with these findings, the fragments were primarily observed at relatively high doses and in some publications represented the final stage before the tubulin lost its structure entirely and became diffusely distributed.

Interestingly, Bis‐ANS (Figure 1), another 1,1^′^‐binaphthyl compound, binds to tubulin and inhibits its polymerization.^[^ ^43^ ^,^ ^44^ ^]^ It features one primary and 40–50 secondary binding sites on tubulin, with the primary, high affinity site being responsible for the tubulin depolymerization and distinct from those sites targeted by colchicine, podophyllotoxin, vinblastine, or maytansine.^[^ ^45^ ^,^ ^46^ ^]^ Curiously, the binding of Bis‐ANS to tubulin was thoroughly explored from 1975 to 2003, but after that, little has been published since on its biological effects. Bis‐ANS is not atropisomeric, but is unlikely to bind to tubulin in its energetically unfavorable coplanar conformation, instead adopting a conformation similar to **1(*R*)**. Therefore, Bis‐ANS and **1(*R*)** may possibly target the same binding site, but further experimentation will be needed to verify this idea.

### 2.6 Compound 1(*R*) Induces a Centrosome Distortion Similar to Colchicine

Given the influence of **1(*R*)** on microtubules, we next examined its impact on the cell centrosomes. With the same treatment conditions as described for the experiment on microtubules, cells were subsequently stained with either alpha‐tubulin and gamma‐tubulin antibodies or alpha‐tubulin and centrin antibodies (Figure 4B,C). Centrin is a centriolar protein and allows the visualization and localization of the centrioles/centrosomes.^[^ ^47^ ^]^ Gamma‐tubulin usually serves the same purpose, but it technically accumulates in the pericentriolar material (PCM) which envelops the centrioles.^[^ ^48^ ^]^

With cells in interphase, the gamma‐tubulin antibody showed several densely red‐colored areas, which made it inappropriate for centrosome localization during this stage of the cell cycle. However, the centrin antibody provided a better visualization of the centrosomes/centrioles. In most interphase cells, the centrioles were thus represented by two distinct red spots, regardless of the treatment compound (Figure 4B). Furthermore, alpha‐tubulin was densely accumulated at the centrioles of interphase cells treated with **1(*R*)** (Figure 4B, white arrow). This insinuates that a substantial amount of tubulin polymerizes at the centrioles, supporting the microtubule depolymerizing effect of **1(*R*)**.

When using the gamma‐tubulin antibody in mitotic cells, we saw two individual red spots at the poles of a bipolar spindle apparatus in cells treated with DMSO and **1(*S*)**, but cells treated with **1(*R*)** showed several spots of varying sizes that overlapped with the microtubule fragments, which is consistent with our alpha‐tubulin observations. In contrast, irrespective of the compound, the centrin antibody displayed just two red spots following treatment.

Depending on the cell cycle, animal cells, with a few exceptions, only form one or two centrosomes, and thus a maximum of four centrioles. Cancer cells often have supernumerary (more than two) centrosomes.^[^ ^49^ ^,^ ^50^ ^]^ Without another regulating step, centrosome amplification (CA) would result in multipolar spindles and consequently aneuploidy, which can contribute to tumorigenesis in some cases, but if it is too intense can be lethal to the cell.^[^ ^51^ ^]^ To avoid this, cancer cells mimic the physiological process and gather the supernumerary centrosomes at two poles, which is called centrosome clustering (CC) and results in a pseudobipolar spindle.^[^ ^52^ ^]^ The loss of spindle pole integrity is an additional mechanism that can result in multipolar spindles and is unrelated to an increase in centrosome occurrence.^[^ ^53^ ^]^

In order to investigate whether the gamma‐tubulin fragments result from the inhibition of CC, we applied **1(*R*)**, **1(*S*)**, or DMSO to the noncancerous bronchial epithelial cell line 16HBE14o‐ and the breast cancer cell line MCF‐7. We selected MCF‐7 because it has been shown in the literature to have exceptionally low levels of centriole amplification, on par to noncancerous cell lines.^[^ ^54^ ^]^ Although **1(*S*)** and DMSO once more displayed no anomalies, **1(*R*)** resulted in multinucleated cells and multipolar spindles that contained gamma‐tubulin fragments inside their aster's center (Figure 3A). Accordingly, it is unlikely that centrosome declustering can account for the effects of **1(*R*)**.

CA is brought on by either a dysregulation of the centriole formation (centriole overduplication, de novo formation of centrioles, fragmentation of overlong centrioles) or a malfunction of pre‐existing structures (cytokinesis‐failure, cell‐fusion).^[^ ^51^ ^]^ Low concentrations of MDAs, such as colchicine, colcemide, and nocodazole, are able to inhibit centriole elongation and higher levels block the initiation of centriole growth.^[^ ^55^ ^,^ ^56^ ^]^ Since **1(*R*)** and colchicine cause similar morphological aberrations and those aberrations are induced by a short (8 h) treatment time, we accordingly find it unlikely that the exceptionally high number of gamma‐tubulin fragments is the result of centriole overduplication, de novo formation of centrioles, or fragmentation of overlong centrioles. This assumption is further supported by the findings that in cells treated with **1(*R*)**, only two centrin spots were observed. As cytokinesis failure and cell‐fusion only cause a low‐grade CA (duplication), they are not appropriate explanations either.

Consequently, a mechanism of PCM fragmentation‐induced spindle pole integrity loss is preferred to explain the effects of **1(*R*)** on mitotic cells. Gamma‐tubulin ring complexes (*γ*TuRCs) are primarily responsible for enforcing microtubule nucleation and it is known that PCM fragmentation can cause acentriolar microtubule nucleating sites.^[^ ^53^ ^]^ Moreover, the MDAs nocodazole and colcemide have been demonstrated to cause PCM fragmentation and it has been proposed for combretastatin A4.^[^ ^35^ ^,^ ^55^ ^,^ ^57^ ^]^ PCM proteins, such as gamma‐tubulin, are not inherently concentrated in their final density at the centrosomes/centrioles and consequently have to be recruited in a process called centrosome maturation. This recruitment can be inhibited by the depolymerization of microtubules.^[^ ^58^ ^]^ With that being the case, **1(*R*)** might disrupt the transportation of PCM proteins due to the shown effects on microtubules. This may later result in a loss of spindle pole integrity and the formation of acentriolar microtubule nucleating sites, which serve as a fundament for multipolar spindles or the observed small microtubule fragments.

### 2.7 Compound 1(*R*) Weakly Inhibits the Polymerization of Pure Tubulin

**Figure 5:**
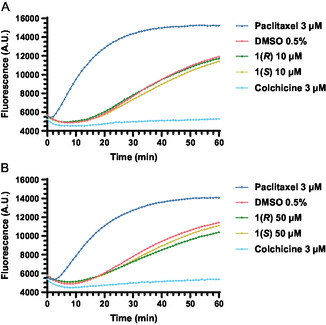
Fluorescence‐based tubulin polymerization assay performed at 37 °C, presented as the mean of three independent experiments. A) 10 µM of **1(*R*)** or **1(*S*)** have no remarkable effect on tubulin polymerization. B) 50 µM of **1(*R*)** slightly inhibits the polymerization of pure tubulin. Due to the intrinsic fluorescence of BINAM, the curves were shifted to the fluorescence of DMSO at time zero.

Based on these results, the influence on the tubulin polymerization was the most obvious possible mode of action. However, in a fluorescence‐based tubulin polymerization assay, **1(*R*)** and **1(*S*)** at 10 µM were unable to greatly affect the polymerization rate in comparison to DMSO (0.5%) (**Figure** **5**A). Nonetheless, at 50 µM, the highest concentration that could be used without visible precipitation, **1(*R*)** noticeably reduced the rate of polymerization, while **1(*S*)** caused a less pronounced decrease (Figure 5B). These results in principle align with the alpha‐tubulin immunostained SISO cells; however, the effect of 50 µM **1(*R*)** on tubulin‐polymerization is relatively weak compared to only 3 µM used with colchicine, which resulted in a dramatic effect on tubulin polymerization. From this, we were able to draw two possible conclusions: First, **1(*R*)** requires the cellular environment to effectively influence microtubules; second, **1(*R*)** is (additionally) able to influence microtubules indirectly. Thus, we examined both of these options through additional experiments.

### 2.8 Compound 1(*R*) Scatters EB1 Similar to Colchicine

EB1 (end‐binding protein 1) is a microtubule‐associated protein (MAP), more specifically a plus‐end tracking protein (+TIP). While MAPs are generally involved in regulating microtubules, +TIPs specifically accumulate on the growing plus‐end of microtubules.^[^ ^59^ ^,^ ^60^ ^]^ The visualization of EB1 therefore allows the tracking of microtubules, with EB1‐rich areas indicating the regions where the majority of microtubules originate.^[^ ^61^ ^]^ The central player in this is typically the centrosome, which is located near the nucleus and serves as the main microtubule‐organizing center (MTOC) in animal cells.

We stained SISO cells with an EB1 antibody after an 8 h treatment with DMSO (0.1%), **1(*R*)** (10.5 µM), **1(*S*)** (10.5 µM), paclitaxel (3.00 µM) or colchicine (3.00 µM) (Figure 4D). Consistent with the above‐described state, EB1 was concentrated around the nucleus in cells treated with DMSO, **1(*S*)** or paclitaxel. In contrast, EB1 was scattered throughout the cell when treated with **1(*R*)** or colchicine. It was previously reported that certain MTAs exhibited the same behavior, which was explained by a disruption in the interaction between EB1 and microtubules.^[^ ^62^ ^,^ ^63^ ^]^ Therefore, we conclude that **1(*R*)**, like colchicine, is able to interfere with the accumulation of EB1 on the growing plus‐ends of microtubules. Another hypothesis is that additional sites for nucleation are formed by the depolymerization, enabling an unstructured growth. In any case, this supports the tubulin‐destabilizing effects observed for **1(*R*)**. Moreover, it was shown that end‐binding proteins are able to strongly enhance the effects of MTAs, which might explain the discrepancy between the effects of **1(*R*)** on cellular microtubules and pure tubulin polymerization.^[^ ^64^ ^]^

### 2.9 Compound 1(*R*) Reduces Detyrosinated Microtubules but has no Effect on Acetylated Ones

There are several post‐translational modifications that tubulins and microtubules can acquire. Acetylated and detyrosinated microtubules are indicative for stabilization and have a slower turnover, although it is still controversial if the stabilization is causative or correlative to the modification.^[^ 65, 66, 67, 68 ^]^ We therefore hypothesized that **1(*R*)** may partially transform or stabilize tubulin. This, in turn, would cause it to be no longer recognized by conventional tubulin antibodies and consequently lead to a perforated appearance of the microtubule cytoskeleton after immunostaining. However, an 8 h treatment with 10.5 µM **1(*R*)** or **1(*S*)** and subsequent immunocytochemistry with antibodies against acetylated or detyrosinated tubulin did not display any remarkable increases of acetylated or detyrosinated microtubules in comparison to the DMSO solvent control (Figure 4E,F). In contrast and consistent with previous research, paclitaxel (3.00 µM) substantially raised the occurrence of both microtubule modifications.^[^ ^69^ ^]^ It is therefore unlikely that **1(*R*)** stabilizes tubulin or rather modifies tubulin due to a prolonged stabilization.

Still, a slight and similar decrease of detyrosinated microtubules in **1(*R*)** and colchicine (3.00 µM) treated cells was observed (Figure 4F), although colchicine had an effect on acetylated microtubules only sparing the centrioles/centrosomes. Overall, this further emphasizes a microtubule destabilizing effect of **1(*R*)**.

### 2.10 Compound 1(*R*) Treatment Scatters Eg5 in Mitotic Cells but has no Effect on KIF1C and Stathmin

Kinesins are a diverse group of motor proteins that move along microtubules. Since the kinesins Eg5 and KIF1C/HSET are essential for centrosome positioning in healthy cells and involved in centrosome clustering in cancer cells, they have been the subject of numerous publications.^[^ ^70^ ^]^ In this context, it was demonstrated that compounds that target Eg5 or KIF1C/HSET are able to decluster centrosomes.^[^ ^71^ ^,^ ^72^ ^]^ Furthermore, it has been shown that both motor proteins are necessary for *γ*TuRCs to migrate to the poles during mitosis.^[^ ^73^ ^]^

**Figure 6:**
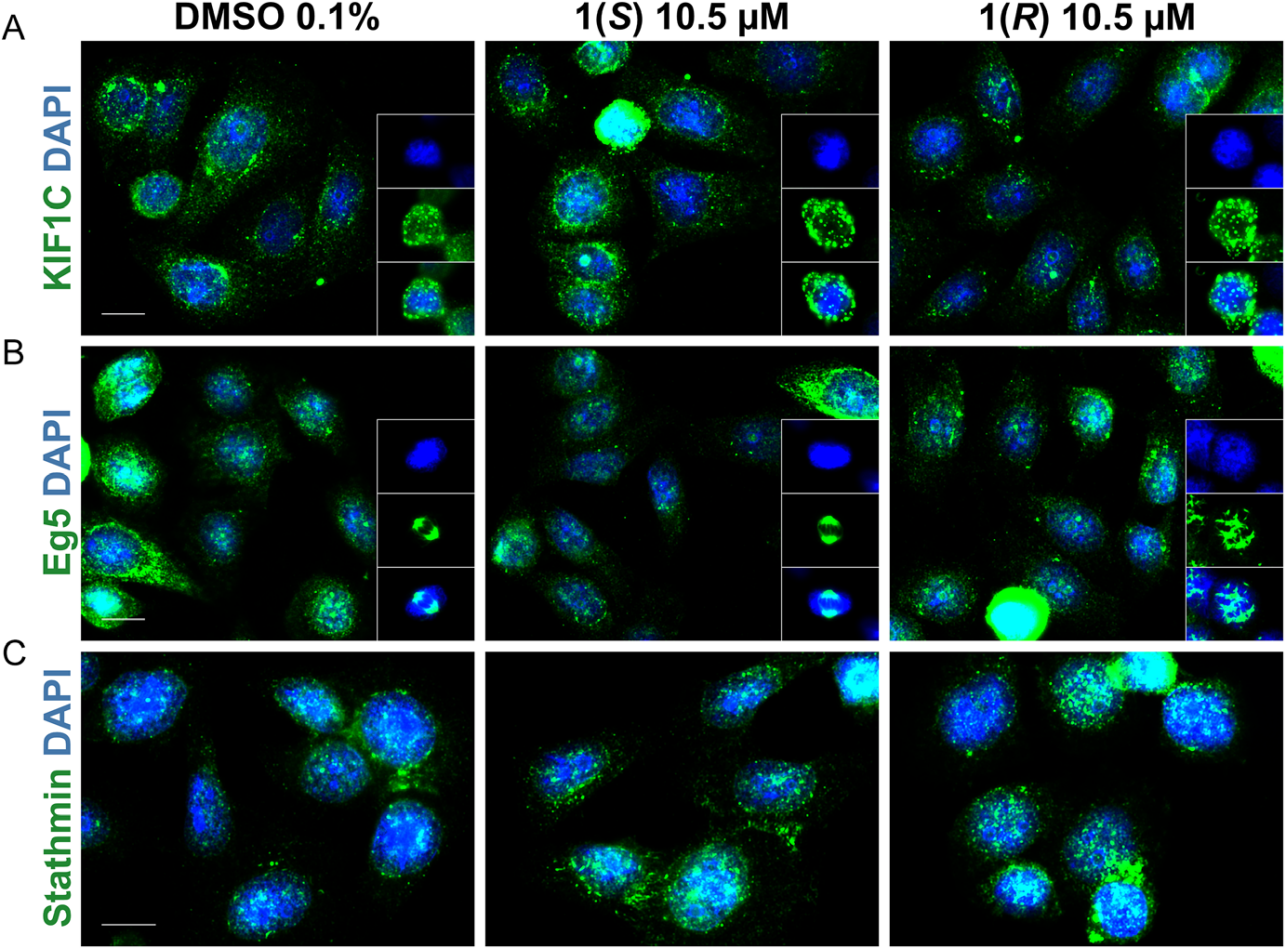
Impact of **1(*R*)** and **1(*S*)** on the motor proteins KIF1C, Eg5, and the microtubule destabilizing protein stathmin. Nuclear DNA is displayed in blue in all images. The scale bar (10 µm) is valid for the respective row of images. A) **1(*R*)** has no effects on the distribution of KIF1C (green). B) Eg5 (green) is diffusely distributed in mitotic cells treated with **1(*R*)**. C) **1(*R*)** treated cells do not show an induction of stathmin (green).

We again treated SISO cells for 8 h with DMSO (0.1%), **1(*R*)** (10.5 µM) or **1(*S*)** (10.5 µM) and subsequently stained them with Eg5 or KIF1C/HSET antibodies. The localization of KIF1C in mitotic cells and both of the motor proteins in interphasic cells were found to be identical in all three treatment conditions (**Figure** **6**A). However, following treatment with **1(*R*)** Eg5 was diffusely distributed in mitotic cells; in contrast, DMSO or **1(*S*)** caused it to be normally concentrated at the spindle apparatus (Figure 6B). Thus, we theorize that this mislocalization of Eg5 is a consequence of the microtubule targeting effects of **1(*R*)**. Eg5 cross‐connects each centrosome by binding to their antiparallel microtubules. Without microtubules, Eg5 is not able to position correctly and direct the centrosomes to the spindle poles, which further contextualizes our findings after alpha‐tubulin, gamma‐tubulin, and centrin staining.

The protein stathmin exerts a destabilizing influence on microtubules, which is crucial for microtubule dynamics throughout the cell cycle.^[^ ^74^ ^]^ Thus, a positive or negative modulation of stathmin expression results in microtubule and spindle abnormalities. However, no variations were noted in stathmin expression among cells treated with DMSO, **1(*R*),** or **1(*S*)** (Figure 6C).

### 2.11 Almost all Major Mitotic Kinases are Unaffected by 1(*R*)

The pyridocarbazole Pyr1, a small‐molecular LIMK1/2 inhibitor, was described to cause stabilized microtubules in HeLa cells without influencing the polymerization of purified tubulin.^[^ ^75^ ^]^ Building on these results, Ramirez‐Rios et al. screened a library of 190 kinase inhibitors on HeLa cells with an assay that used a concomitant combrestatin A4 treatment.^[^ ^76^ ^]^ They identified seven compounds that induce stabilized microtubule cytoskeletons but did not influence the microtubule assembly of purified tubulin and did not bind tubulin directly, namely, SNS‐314 mesylate (Aurora A/B/C), selonsertib (ASK1), PF0477736 (CHK1/2), MPI0479605 (Mps1/TTK), masitinib (tyrosine kinases), ponatinib and nintedanib (RTKs), and SR10905, SR10854, and SR10847 (LIMK). For LIMK1/2,^[^ ^77^ ^,^ ^78^ ^]^ Aurora A^[^ ^79^ ^]^ and CHK1/2^[^ ^80^ ^]^ an influence on microtubule dynamics has been described before and Mps1/TTK is known to regulate the stability of the kinetochore‐microtubule attachment.^[^ ^81^ ^]^

Based on this knowledge, we commissioned a kinase screening by the firm Reaction Biology, targeting those kinases whose inhibition has shown an indirect effect on microtubules. In our selection, we included additional kinases that are known to be involved in centrosome amplification (PLK4^[^ ^82^ ^]^), centrosome clustering (CDK1/2,^[^ ^83^ ^]^ PLK1^[^ ^84^ ^]^), spindle multipolarity (SRC^[^ ^85^ ^]^), microtubule dynamics (AKT1^[^ ^86^ ^]^), and LIMK1/2 activation (ROCK1^[^ ^87^ ^]^) or are crucial for the mitosis (BUB1B, NEK2/9, WEE1^[^ ^88^ ^]^). Nonetheless, **1(*R*)** and **1(*S*)** at 10 µM had almost no inhibitory effects on any of the chosen kinases (**Table** **4**). Only Aurora A (mean residual activity for **1(*R*)**: 86%, **1(*S*)**: 98%) and ASK1 (mean residual activity for **1(*R*)**: 90%, **1(*S*)**: 117%) were slightly enantioselectively impacted. However, we theorize that those marginal inhibitions are not explanatory for the antiproliferative and morphological effects of **1(*R*)** on cancer cells.

**Table 4:** Screening of important mitotic kinases.

Kinasea)	Residual activities [% of control]		
	1(*R*) 10 µM	1(*S*) 10 µM	Staurosporine 100 µM
AKT1	99	101	0
ASK1	90	117	0
AuroraA	86	98	2
AuroraB	101	101	7
AuroraC	101	100	1
BUB1B	118	111	56
CDK1/CycB1	101	100	2
CDK2/CycA2	95	92	0
CHK1	94	95	0
CHK2	97	104	−1
LIMK1	103	103	−1
LIMK2	106	98	2
Mps1/TTK	98	97	0
NEK2	106	96	12
NEK9	94	96	1
PLK1	95	91	3
PLK4	97	99	−2
ROCK1	100	97	0
SRC	99	103	0
WEE1	99	98	4

a) Mean of intraexperimental duplicates (*n* = 1).

### 2.12 A Re‐evaluation of Binaphthyl Toxicity is Necessary

Our findings are also of toxicological relevance due to the importance and prominence of binaphthyls as reagents, especially for organometallic catalysts used in enantioselective synthesis. On SciFinder, there are currently (March 6, 2025) 2,589 reactions described for BINAM and 17,397 for BINOL (racemates and enantiomers). As of now, the GHS hazard statements warn that **1(*R*)** “only” causes serious eye irritation (H319), skin irritation (H315), and respiratory irritation (H335); therefore, the toxicity of **1(*R*)** and other binaphthyls on human cells might be drastically underestimated. Additionally, the harmful potential of binaphthyls is demonstrated by the recognized carcinogenicity of 7*H*‐dibenzo[*c*,g]carbazole (**5**) and its metabolites.^[^ ^89^ ^]^ In light of our findings, a re‐evaluation of the gene toxicity of binaphthyls would seem appropriate.

## 3 Conclusion

In summary, we report for the first time that atropisomeric **1(*R*)** is an enantioselective and moderately potent spindle poison that acts quickly to inhibit the proliferation of human cancer cells. Our investigations into the source of the antiproliferative activity revealed that **1(*R*)** induces G2/M arrest, mitotic catastrophe, multinucleated cells, and apoptosis. By further microscopic examination, we found **1(*R*)** to cause a depolymerization of microtubules, multipolar spindles, and signs of PCM fragmentation similar to CBSIs and other MDAs. Furthermore, a treatment with **1(*R*)** caused the scattering of EB1, which usually is concentrated around the centrosome. Eg5, normally localized at the spindle apparatus in mitotic cells, was diffusely distributed as well. To rule out indirect microtubule targeting, we conducted a kinase screening that revealed no significant inhibition of the major mitotic kinases. Thus, it is hypothesized that **1(*R*)** induces microtubule depolymerization directly, resulting in the loss of spindle pole integrity due to PCM fragmentation. Administering micromolar concentrations leads to the formation of multipolar spindles, whereas higher concentrations cause total fragmentation of the spindle apparatus. Ultimately, nonviable multinucleated cells are generated, leading to apoptosis. Through the synthesis of various binaphthyl analogs, we identified additional antiproliferative compounds (**11(*R*)**, **12(*R*)**, **12(*S*)**, **15(*R*)**); two of them exhibited the same enantiomeric trend as BINAM. Although we could not ascertain the binding site of **1(*R*)** and the other active binaphthyls, we demonstrated a remarkable similarity in the effect of **1(*R*)** to colchicine and other MDAs. Therefore, atropisomeric binaphthyls may be interesting scaffolds for the development of more potent and selective anticancer drugs.

## 4 Experimental Section

4.1

4.1.1

##### General Procedures

Binaphthyls **1(*R*)**, **1(*S*)**, **13(*R*)**, **13(*S*)**, **18(*R*)**, and **18(*S*)** were purchased from BLDPharm. All used chemicals were obtained in high purity, stored in accordance with the manufacturer's instructions and unless specified used without additional purification. Toluene was freshly distilled and dried with 3 Å molecular sieve. Every other solvent was bought water‐free in airtight septa‐sealed bottles. If stated, glassware was dried in an oven at 120 °C overnight and solvents degassed by using the freeze‐pump‐thaw method. For analytical TLC, aluminum sheets (10 × 10 cm) coated with TLC silica gel 60 Å F_254_ (Merck) were used. Silica gel 60 Å, 0.04–0.063 mm (400–230 mesh) (Roth), was used for column chromatography. Celite 545 (Roth) was utilized for dry loading compounds or for filtration. A Bruker Avance III with resonance frequencies of 400 MHz for ^1^H and 100 MHz for ^13^C was used to acquire nuclear NMR spectra. The chemical shift was determined by using tetramethylsilane (TMS) or the residual solvent signal as reference and expressed in parts per million (ppm). The coupling constants (*J*) are expressed in Hz. Splitting patterns were denoted using the common abbreviations: s = singlet, d = doublet, t = triplet, dt = doublet of triplets, dddd = doublet of doublet of doublet of doublets, m = multiplet, br = broad. To measure HRMS data, a Bruker compact with ESI or APCI ionization was utilized. Reversed phase (RP)‐HPLC was performed on a Merck Hitachi D‐7000 interface, L‐7100 pump, L‐7200 autosampler, L‐7450 diode array detector along with a Merck L‐7350 column oven, and L‐7612 solvent degasser. Chiral HPLC was performed on a Merck Hitachi D‐7000 interface, L‐7110 pump, L‐7420 UV‐Vis‐detector along with a Merck L‐7350 column oven, and Degasys DG‐1310 solvent degasser. The purity of all compounds was determined by RP‐HPLC to be >95%. The enantiomerical purity of all measurable compounds was >95% as well. Our highly polar compounds were not measurable by using a Chiralcel OD‐H (5 μm, 4.6 × 250 mm), resulting in an excessive peak broadening, which is why CD was additionally used as a proof of chirality for all compounds. CD spectra were obtained with a Jasco J‐710 Spectropolarimeter and 0.03 mM solution of the respective compound in MeCN.

##### (*R*)‐*N*,*N*′‐([1,1′‐Binaphthalene]‐2,2′‐diyl)diacetamide (2)

An oven‐dried 50 mL round‐bottom flask containing a magnetic stirrer bar was charged with **1(*R*)** (200 mg, 0.7 mmol, 1.0 equiv), which was dissolved in pyridine (20 mL). Acetic anhydride (0.182 mL, 1.9 mmol, 2.7 equiv) was added and the reaction stirred for 24 h at rt. The pyridine was removed by rotary evaporation. Then, the crude product was dissolved in EtOAc, washed three times with 0.1 M HCl and once with water. The organic phase was dried with Na_2_SO_4_, filtered, and concentrated. The solid crude material was then dissolved in a minimal amount of EtOAc and purified by column chromatography with EtOAc/petroleum ether 70:30 to obtain **2** (147 mg, 54%) as a yellow‐white waxy substance. *R*_f_ (EtOAc/petroleum ether 70:30) = 0.27. ^1^H NMR (DMSO‐*d*6, 400 MHz): *δ* 1.72 (s, 6H), 6.83 (d, 2H, *J* = 8.5 Hz), 7.24 (dt, 2H, *J* = 6.9, 1.0 Hz), 7.45 (dt, 2H, *J* = 7.9, 0.9 Hz), 7.87 (d, 2H, *J* = 8.7 Hz), 8.00 (d, 2H, *J* = 8.1 Hz), 8.05 (d, 2H, *J* = 8.9 Hz), 8.94 (s, 2H) ppm. ^13^C NMR (DMSO‐*d*6, 100 MHz): *δ* 22.9, 124.9, 125.2, 125.7, 126.4, 128.0, 128.5, 131.2, 132.5, 135.2, 169.2 ppm. HRMS calcd for C_24_H_21_N_2_O_2_ (M + H) 369.1598; found, 369.1601.

##### (*R*)‐*N*,*N*′‐([1,1′‐Binaphthalene]‐2,2′‐diyl)dimethanesulfonamide (3)

Under an argon atmosphere, an oven‐dried 100 mL three‐neck round‐bottom flask containing a magnetic stirrer bar was charged with **1(*R*)** (853 mg, 3 mmol, 1.0 equiv), which was dissolved in CH_2_Cl_2_ (24 mL) and pyridine (6 mL). Mesyl chloride (1 mL, 13.2 mmol, 4.4 equiv) was added and the reaction was stirred for 24 h at rt. The mixture was acidified with 1 M HCl (150 mL) and extracted with CH_2_Cl_2_ (3 × 150 mL). The organic phases were collected, dried with Na_2_SO_4_, filtered, and concentrated. The crude product was dried overnight under high vacuum. The solid crude material was then dissolved in a minimal amount of EtOAc, concentrated on celite, and purified by column chromatography with toluene → EtOAc/toluene 30:70 to obtain **3** (810 mg, 61%) as a white foam. *R*_f_ (EtOAc/heptane 40:60) = 0.38. ^1^H NMR (DMSO‐*d*6, 400 MHz): *δ* 2.89 ppm (s, 6H), 6.84 (d, 2H, *J* = 8.0 Hz), 7.29 (dt, 2H, *J* = 6.8, 1.2 Hz), 7.46 (dt, 2H, *J* = 6.8, 1.1 Hz), 7.89 (d, 2H, *J* = 9.0 Hz), 8.02 (d, 2H, *J* = 8.0 Hz), 8.14 (d, 2H, *J* = 9.0 Hz), 8.28 (s, 2H) ppm. ^13^C NMR (DMSO‐*d*6, 100 MHz): *δ* 41.2, 121.8, 124.0, 125.2, 125.4, 126.9, 128.1, 128.2, 128.9, 129.7, 130.9, 132.7, 134.8 ppm. HRMS calcd for C_22_H_21_N_2_O_4_S_2_ (M + H) 441.0937; found, 441.0937.

##### (*R*)‐*N*,*N*′‐([1,1′‐Binaphthalene]‐2,2′‐diyl)dicyanamide (4)

Under an argon atmosphere, an oven‐dried 100 mL three‐neck round‐bottom flask containing a magnetic stirrer bar was charged with **1(*R*)** (853 mg, 3 mmol, 1 equiv) and NaHCO_3_ (882 mg, 10.5 mmol, 3.5 equiv). Dry and degassed MeCN (20 mL) was added. BrCN (953 mg, 9 mmol, 3 equiv) was quickly dissolved in MeCN (10 mL) and injected under stirring into the flask. Afterward, the reaction was stirred at 50 °C (external temperature) for 3 days. The mixture was cooled to rt and quenched successively with water, 1 M NaOH, and H_2_O_2_ and stirred for 15 min. This waterous phase was extracted with EtOAc (3 × 250 mL). The organic phases were collected, dried with Na_2_SO_4_, filtered, and concentrated. Then, the crude material was dissolved in a minimal amount of CH_2_Cl_2_, concentrated on celite and purified by column chromatography with hexane → EtOAc/hexane 40:60 to obtain **4** (136 mg, 13%) as a brown solid. *R*_f_ (EtOAc/hexane 30:70) = 0.20. ^1^H NMR (DMSO‐*d*6, 400 MHz): *δ* 6.95 (d, 2H, *J* = 8.6 Hz), 7.26 (dt, 2H, *J* = 6.8, 1.2 Hz), 7.45–7.49 (m, 4H), 8.01 (d, 2H, *J* = 8.0 Hz), 8.06 (d, 2H, *J* = 8.8 Hz), 9.94 (s, 2H). ^13^C NMR (DMSO‐*d*6, 100 MHz): *δ* 115.3, 122.5, 123.7, 125.4, 126.1, 126.5, 128.3, 129.6, 131.1, 131.4, 140.6, 169.5 ppm. HRMS calcd for C_22_H_15_N_4_ (M + H) 335.1291; found, 335.1292.

##### *7H*‐Dibenzo[*c*,*g*]carbazole (5)

Under an argon atmosphere in an oven‐dried Schlenk tube containing a magnetic stirrer bar, **1(*R*)** (569 mg, 2.0 mmol, 1.0 equiv) was dissolved in 1,4‐dioxane (12 mL). 12 M aqueous HCl solution (930 µL) was added, followed by stirring for 26.5 h at reflux. The reaction mixture was cooled to rt and neutralized with a saturated aqueous NaHCO_3_ solution. The reaction mixture was diluted with water to 150 mL and extracted with CH_2_Cl_2_ (3 × 150 mL). The organic phases were combined, dried with Na_2_SO_4_, filtered, and concentrated. The crude product was purified by column chromatography with toluene to obtain **5** (214 mg, 40%) as white‐yellow crystals. *R*_f_ (EtOAc/hexane 30:70) = 0.56. ^1^H NMR (DMSO‐*d*6, 400 MHz): *δ* 7.52 (dt, 2H, *J* = 7.8, 0.7 Hz), 7.71 (dt, 2H, *J* = 8.3, 1.3 Hz), 7.84 (d, 2H, *J* = 8.7), 7.94 (d, 2H, *J* = 8.7 Hz), 8.11 (dd, 2H, *J* = 8.2, 1.0 Hz), 9.09 (d, 2H, *J* = 8.4 Hz), 12.34 (s, 1H) ppm. ^13^C NMR (DMSO‐*d*6, 100 MHz): *δ* 113.8, 116.3, 123.1, 124.4, 125.6, 126.5, 128.8, 129.5, 129.6, 136.8 ppm. HRMS calcd for C_20_H_14_N (M + H) 268.1121; found, 268.1123.

##### (*R*)‐*N* ^2^,*N* ^2^′‐Di(pyridin‐2‐yl)‐[1,1′‐binaphthalene]‐2,2′‐diamine (6)

Under an argon atmosphere, an oven‐dried Schlenk tube containing a magnetic stirrer bar was charged with Pd_2_(dba)_3_ (46 mg, 0.05 mmol, 0.05 equiv) and Xantphos (58 mg, 0.1 mmol, 0.1 equiv). Dry toluene (5 mL) was added and the reaction mixture stirred at rt for 15 min. Then **1(*R*)** (284 mg, 1.0 mmol, 1.0 equiv), 2‐bromopyridine (244 µL, 2.5 mmol, 2.5 equiv), and NaO^*t*^Bu (288 mg, 3 mmol, 3 equiv) were added and the reaction mixture stirred at 100 °C (external temperature) for 43 h. The mixture was cooled to rt and filtered over a pad of celite. The pad was washed with 100 mL EtOAc and the filtrate washed with water (2 × 100 mL), dried with Na_2_SO_4_, filtered, and concentrated. The crude product was dissolved in a minimal amount of EtOAc, concentrated on celite, and purified by column chromatography with EtOAc/hexane 30:70 to obtain **6** (110 mg, 25%) as an orange‐brown solid. *R*_f_ (EtOAc/hexane 50:50) = 0.23. ^1^H NMR (DMSO‐*d*6, 400 MHz): *δ* 6.62–6.65 (m, 4H), 6.96 (d, 2H, *J* = 8.4 Hz), 7.19 (dt, 2H, *J* = 6.8, 1.2 Hz), 7.35 (dt, 2H, *J* = 6.9, 1.0 Hz), 7.40 (dt, 2H, *J* = 7.2, 1.9 Hz), 7.85 (s, 2H), 7.92–8.03 (m, 8H) ppm. ^13^C NMR (DMSO‐*d*6, 100 MHz): *δ* 109.8, 114.4, 123.5, 124.3, 124.3, 124.8, 126.2, 128.0, 128.4, 130.4, 133.0, 137.4, 138.0, 147.4, 156.4 ppm. HRMS calcd for C_30_H_23_N_4_ (M + H) 439.1917; found, 439.1924.

##### (*R*)‐*N* ^2^‐(Pyrimidin‐2‐yl)‐[1,1′‐binaphthalene]‐2,2′‐diamine (7)

Under an argon atmosphere, an oven‐dried Schlenk tube containing a magnetic stirrer bar was charged with Pd_2_(dba)_3_ (27 mg, 0.03 mmol, 0.01 equiv) and Xantphos (26 mg, 0.045 mmol, 0.15 equiv). Dry toluene (5 mL) was added and the reaction mixture stirred at rt for 15 min. Then **1(*R*)** (853 mg, 3.0 mmol, 1.0 equiv), 2‐chloropyrimidine (413 mg, 3.6 mmol, 1.2 equiv), and NaO^*t*^Bu (288 mg, 3 mmol, 1 equiv) were added and the reaction mixture was stirred at 100 °C (external temperature) for 73 h. The mixture was cooled to rt and filtered over a pad of celite. The pad was washed with 150 mL EtOAc and the filtrate was washed with water (3 × 150 mL), dried with Na_2_SO_4_, filtered, and concentrated. The crude product was purified by column chromatography with EtOAc/toluene 20:80 to obtain **7** (253 mg, 23%) as a white solid. *R*_f_ (EtOAc/hexane 30:70) = 0.16. ^1^H NMR (DMSO‐*d*6, 400 MHz): *δ* 4.91 (s, 2H), 6.68 (d, 1H, *J* = 8.2 Hz), 6.83 (t, 1H, *J* = 4.8 Hz), 7.01 (d, 1H, *J* = 8.4 Hz), 7.07–7.15 (m, 2H), 7.22–7.29 (m, 3H), 7.39 (dt, 1H, *J* = 6.8, 1.1 Hz), 7.80 (d, 1H, *J* = 8.4 Hz), 7.86 (d, 1H, J = 8.8 Hz), 7.97 (d, 1H, *J* = 8.0 Hz), 8.07 (d, 1H, *J* = 9.1 Hz), 8.38 (d, 2H, *J* = 4.8 Hz), 8.78 (d, 1H, *J* = 9.1 Hz) ppm. ^13^C NMR (DMSO‐*d*6, 100 MHz): *δ* 108.4, 113.1, 118.6, 120.0, 120.4, 121.4, 122.6, 124.1, 124.9, 126.4, 126.6, 127.0. 128.1, 128.1, 128.3, 129.6, 130.1, 132.6, 133.6, 136.1, 144.6, 158.2, 159.5 ppm. HRMS calcd for C_24_H_19_N_4_ (M + H) 363.1604; found, 363.1615.

##### (*R*)‐*N* ^2^,*N* ^2^′‐Bis(5‐methoxypyrimidin‐2‐yl)‐[1,1′‐binaphthalene]‐2,2′‐diamine (8)

Under an argon atmosphere, an oven‐dried Schlenk tube containing a magnetic stirrer bar was charged with Pd_2_(dba)_3_ (46 mg, 0.05 mmol, 0.05 equiv) and Xantphos (58 mg, 0.1 mmol, 0.1 equiv). Dry toluene (5 mL) was added and the reaction mixture stirred at rt for 15 min. Then **1(*R*)** (284 mg, 1.0 mmol, 1.0 equiv), 2‐chloro‐5‐methoxypyrimidine (434 mg, 3.0 mmol, 3.0 equiv), and NaO^*t*^Bu (288 mg, 3.0 mmol, 3.0 equiv) were added and the reaction mixture was stirred at 100 °C (external temperature) for 23 h 10 min. The mixture was cooled to rt and filtered over a pad of celite. The pad was washed with EtOAc (100 mL) and the filtrate with water (100 mL). The aqueous phase was extracted with EtOAc (3 × 100 mL). The combined organic phases were dried with Na_2_SO_4_, filtered, and concentrated. The crude product was dissolved in a minimal amount of EtOAc, concentrated on celite, and purified by DCVC with hexane → EtOAc/hexane 50:50 to obtain **8** (169 mg, 34%) as a yellow solid. *R*_f_ (EtOAc/hexane 50:50) = 0.62. ^1^H NMR (DMSO‐*d*6, 400 MHz): *δ* 3.73 (s, 6H), 5.76 (s, 2H), 6.96 (d, 2H, *J* = 8.6 Hz), 7.21 (dt, 2H, *J* = 6.8, 1.2 Hz), 7.37 (dt, 2H, *J* = 6.9, 1.1 Hz), 7.70 (s, 2H), 7.96 (d, 2H, *J* = 8.0 Hz), 8.06 (d, 2H, *J* = 9.0 Hz), 8.11 (s, 4H), 8.27 (d, 2H, *J* = 9.0 Hz) ppm. ^13^C NMR (DMSO‐*d*6, 100 MHz): *δ* 54.9, 56.4, 122.1, 122.4, 124.4, 124.5, 126.5, 128.0, 128.7, 130.2, 132.5, 137.4, 144.6, 147.5, 155.2 ppm. HRMS calcd for C_30_H_25_N_6_O_2_ (M + H) 501.2034; found, 501.2042.

##### Diethyl [1,1′‐binaphthalene]‐2,2′‐diyl(R)‐dicarbamate (9)

Under an argon atmosphere in an oven‐dried three‐necked round‐bottom flask containing a magnetic stirrer bar, **1(*R*)** (1.42 g, 5.0 mmol, 1.0 equiv) was dissolved in dry CH_2_Cl_2_ (43 mL). Pyridine (3.2 mL, 39 mmol, 7.8 equiv) was added dropwise with a syringe. The reaction mixture was cooled to 2 °C (internal temperature) and freshly distilled ethyl chloroformate (1.05 mL, 11 mmol, 2.2 equiv) was added dropwise with a syringe. Then, the reaction mixture was stirred for 1 h at 2–4 °C (internal temperature) and another 30 min at rt. A 2 M aqueous KOH solution (27 mL) was added dropwise followed by stirring for 20 min at rt. The organic phase was separated and the aqueous phase extracted with CH_2_Cl_2_ (3 × 15 mL). The organic phases were combined, washed with 1 M aqueous HCl solution (2 × 40 mL) and brine (40 mL), dried with Na_2_SO_4_, filtered, and concentrated to obtain **9** (2.1 g, 98%) as a white foam. *R*_f_ (EtOAc/hexane 40:60) = 0.60. ^1^H NMR (DMSO‐*d*6, 400 MHz): *δ* 1.00 (t, 6H, *J* = 7.1 Hz), 3.84–3.97 (dddd, 4H), 6.86 (d, 2H, *J* = 8.5 Hz), 7.26 (dt, 2H, *J* = 6.9, 1.3 Hz), 7.45 (dt, 2H, *J* = 6.9, 1.0 Hz), 7.87 (d, 2H, *J* = 8.9 Hz), 8.00 (d, 2H, *J* = 8.1 Hz), 8.09 (d, 4H, *J* = 8.6 Hz) ppm. ^13^C NMR (DMSO‐*d*6, 100 MHz): *δ* 14.2, 60.3, 124.0, 124.8, 125.3, 125.6, 126.6, 128.0, 128.9, 131.1, 132.1, 135.0, 154.4 ppm. HRMS calcd for C_26_H_25_N_2_O_4_ (M + H) 429.1809; found, 429.1806.

##### (*R*)‐*N* ^2^,*N* ^2^′‐Dimethyl‐[1,1′‐binaphthalene]‐2,2′‐diamine (10)

Under an argon atmosphere, dry and degassed THF (25 mL) was injected into an oven‐dried 250 mL three‐neck round‐bottom flask. The THF was cooled down to ≈2 °C (internal temperature) with an ice‐water bath. Under an increased argon flow LiAlH_4_ (1.67 g, 44 mmol, 11 equiv) was added portion wise over 15 min. The resulting suspension was stirred for 10 min. **9** (1.71 g, 4 mmol, 1 equiv) was dissolved in THF (8.5 mL) in an oven‐dried flask and added to the reaction mixture dropwise over 15 min, and the flask was rinsed with THF (2 × 2 mL), which was injected as well. The ice‐water bath was replaced by an oil bath and the reaction mixture stirred under reflux for 17 h. The mixture was cooled to rt and purified with the Fieser workup. Diethylether (130 mL) was added and the mixture was cooled for 10 min in an ice‐water bath. Consecutively, water (1.7 mL), 3 M NaOH (1.7 mL), and more water (5.1 mL) were added and this mixture stirred for another 10 min. Celite (3.5 g) was added and the resulting slurry was filtered using a glass frit. The filter cake was rinsed with EtOAc and the filtrate dried with Na_2_SO_4_, filtered, and concentrated under high vacuum to afford **10** (1.20 g, 96%) as a brown solid. *R*_f_ (EtOAc/hexane 17:83) = 0.47. ^1^H NMR (DMSO‐*d*6, 400 MHz): *δ* 2.73 (s, 6H), 4.16 (s, 2H), 6.70 (dd, 2H, *J* = 8.5, 1.7, 0.7 Hz), 7.09 (dddd, 4H, *J* = 14.1, 11.8, 6.7, 1.6 Hz), 7.26 (d, 2H, *J* = 9.0), 7.79 (dd, 2H, *J* = 7.2, 2.0 Hz), 7.92 (d, 2H, *J* = 8.9 Hz) ppm. ^13^C NMR (DMSO‐*d*6, 100 MHz): *δ* 30.3, 110.7, 113.6, 120.9, 123.0, 126.1, 126.9, 128.1, 129.2, 133.4, 145.5 ppm. HRMS calcd for C_22_H_21_N_2_ (M + H) 313.1699; found, 313.1698.

##### (*R*)‐2′‐Amino‐[1,1′‐binaphthalene]‐2‐ol (11(*R*))

**1(*R*)** (1 g, 3.52 mmol, 1 equiv) was dissolved in 1,4‐dioxane (230 mL), 12 M HCl (47 mL), and water (65 mL). To this solution, benzoyl peroxide (with 25% water) (3.07 g, 9.5 mmol, 2.7 equiv) was added portion wise. After stirring at 85 °C (external temperature) for 6 h, the reaction mixture was cooled to rt, neutralized with 2 M NaOH until the pH was ≈8, and then concentrated by rotary evaporation. The crude material was transferred to a separatory funnel with EtOAc (300 mL) and water (300 mL) and extracted. The organic phase was washed with water (2 × 300 mL), dried with Na_2_SO_4_, filtered, and concentrated. The residue was purified by column chromatography with EtOAc/toluene 5:95 to afford **11(*R*)** (300 mg, 30%) as a beige solid. *R*_f_ (EtOAc/hexane 25:75) = 0.30. ^1^H NMR (DMSO‐*d*6, 400 MHz): *δ* 4.54 (s, 2H), 6.74–6.77 (m, 1H), 6.94 (d, 1H, *J* = 8.4 Hz), 7.05–7.11 (m, 2H), 7.19 (dt, 2H, *J* = 7.2, 1.4 Hz), 7.25 (dt, 2H, *J* = 6.8, 1.2 Hz), 7.36 (d, 1H, *J* = 8.9 Hz), 7.72–7.24 (m, 2H), 7.88 (t, 2H, *J* = 8.8 Hz), 9.28 (s, 1H) ppm. ^13^C NMR (DMSO‐*d*6, 100 MHz): *δ* 111.2, 114.9, 118.4, 118.8, 120.7, 122.5, 123.4, 124.1, 125.7, 126.1, 127.0, 127.8, 128.0, 128.1, 128.4, 129.1, 133.6, 134.0, 143.9, 153.3 ppm. HRMS calcd for C_20_H_16_NO (M + H) 286.1226; found, 286.1220.

##### (*S*)‐2′‐Amino‐[1,1′‐binaphthalene]‐2‐ol (11(*S*))

**11(*S*)** was prepared in accordance with **11(*R*)** to afford a beige solid (269 mg, 27%). *R*_f_ (EtOAc/hexane 30:70) = 0.50. ^1^H NMR (DMSO‐*d*6, 400 MHz): *δ* 4.54 (s, 2H), 6.74–6.77 (m, 1H), 6.94 (d, 1H, *J* = 8.4 Hz), 7.05–7.11 (m, 2H), 7.19 (dt, 2H, *J* = 7.9, Hz), 7.25 (dt, 2H, *J* = 7.0, 1.2 Hz), 7.36 (d, 1H, *J* = 9.0 Hz), 7.72–7.74 (m, 2H), 7.88 (t, 2H, *J* = 8.9 Hz), 9.28 (s, 1H) ppm. ^13^C NMR (DMSO‐*d*6, 100 MHz): *δ* 111.2, 114.9, 118.4, 118.8, 120.8, 122.5, 123.5, 124.1, 125.7, 126.1, 127.0, 127.8, 128.1, 128.1, 128.5, 129.1, 133.6, 134.0, 143.9, 153.3 ppm. HRMS calcd for C_20_H_16_NO (M + H) 286.1226; found 286.1220.

##### (*R*)‐3,5‐Dihydro‐4*H*‐dinaphtho[2,1‐*d*:1′,2′‐*f*[1,3] diazepin‐4‐imine (12(*R*))

Under an argon atmosphere, an oven‐dried Schlenk tube containing a magnetic stirrer bar was charged with **1(*R*)** (853 mg, 3.0 mmol, 1.0 equiv), which was dissolved in dry MeCN (7.5 mL). The reaction mixture was cooled down to 0 °C (internal temperature) with an ice‐water bath. Now, 5 mL of a 1 M BrCN solution in MeCN was prepared in a dry flask, from which 3 mL were injected dropwise into the Schlenk tube. A reflux condenser was added and the reaction mixture was stirred at reflux for 22.5 h. The reaction mixture was cooled to rt and concentrated. The solid crude material was dissolved in MeOH/CH_2_Cl_2_ 10:90 and purified with a silica plug. The filtrate was dried with Na_2_SO_4_, filtered, and concentrated to afford **12(*R*)** (772 mg, 83%) as a light brown solid. *R*_f_ (MeOH/CH_2_Cl_2_ 10:90) = 0.10. ^1^H NMR (DMSO‐*d*6, 400 MHz): *δ* 6.96 (d, 2H, *J* = 8.5 Hz), 7.31 (dt, 2H, *J* = 6.9, 1.2 Hz), 7.44 (d, 2H, *J* = 8.8 Hz), 7.52 (dt, 2H, *J* = 7.0, 0.9 Hz), 8.06 (d, 2H, *J* = 8.0 Hz), 8.15 (d, 2H, *J* = 8.8 Hz), 8.44 (br s, 1H), 10.28 (br s, 2H) ppm. ^13^C NMR (DMSO‐*d*6, 100 MHz): *δ* 121.9, 123.9, 125.9, 126.1, 126.8, 128.4, 130.1, 131.4, 131.5, 139.4, 165.6 ppm. HRMS calcd for C_21_H_16_N_3_ (M + H) 310.1339; found, 310.1340.

##### (*S*)‐3,5‐Dihydro‐4*H*‐dinaphtho[2,1‐*d*:1′,2′‐*f*[1,3] diazepin‐4‐imine (12(*S*))

**12(*S*)** was prepared in accordance with **12(*R*)** using 3 mmol of **1(*S*)** to afford a light brown solid (477 mg, 51%). *R*_f_ (MeOH/CH_2_Cl_2_ 10:90) = 0.14. ^1^H NMR (DMSO‐*d*6, 400 MHz): *δ* 6.97 (d, 2H, *J* = 8.5 Hz), 7.30 (dt, 2H, *J* = 6.9, 1.3 Hz), 7.48–7.54 (m, 4H), 8.07 (d, 2H, *J* = 8.0 Hz), 8.16 (d, 2H, *J* = 8.7 Hz), 8.71 (br s, 1H), 10.18 (br s, 2H) ppm. ^13^C NMR (DMSO‐*d*6, 100 MHz): *δ* 121.8, 123.9, 125.8, 126.1, 126.8, 128.4, 130.1, 131.4, 131.5, 139.5, 165.8 ppm. HRMS calcd for C_21_H_16_N_3_ (M + H) 310.1339; found, 310.1344.

##### (*R*)‐[1,1′‐Binaphthalene]‐2,2′‐dicarbonitrile (14(*R*))

Under an argon atmosphere, an oven‐dried Schlenk tube containing a magnetic stirrer bar was charged with **13(*R*)** (11 g, 20 mmol, 1 equiv), NiCl_2_(dppe) (1.09 g, 2 mmol, 0.1 equiv), DPPE (800 mg, 2 mmol, 0.1 equiv), Zn(CN)_2_ (4.70 g, 40 mmol, 2 equiv), and zinc powder (393 mg, 6 mmol, 0.3 equiv). Dry and degassed DMF (30 mL) was added and the reaction stirred at 145 °C (external temperature) for 4 d 20 h. The mixture was cooled to rt and filtered over a pad of celite and after that over a pad of silica gel 60 (0.04–0.063 µM). The filtrate was concentrated by rotary evaporation and then dried under high vacuum. The crude material was dissolved in EtOAc (250 mL) and washed with water (2 × 250 mL). The organic phase was dried with Na_2_SO_4_, filtered, and concentrated. Then, the crude material was dissolved in a minimal amount of EtOAc, concentrated on celite, and purified by column chromatography with EtOAc/hexane 25:75 to afford **14(*R*)** (5.02 g, 80%) as a white crystalline solid. *R*_f_ (EtOAc/hexane 30:70) = 0.46. ^1^H NMR (DMSO‐*d*6, 400 MHz): *δ* 7.11 (d, 2H, *J* = 8.5 Hz), 7.56 (dt, 2H, *J* = 7.0, 1.2 Hz), 7.80 (dt, 2H, *J* = 7.0, 1.0 Hz), 8.12 (d, 2H, *J* = 8.6 Hz), 8.27 (d, 2H, *J* = 8.2 Hz), 8.42 (d, 2H, J = 8.5 Hz) ppm. ^13^C NMR (DMSO‐*d*6, 100 MHz): *δ* 110.6, 117.5, 125.8, 126.7, 128.9, 129.0, 129.6, 130.8, 131.0, 134.6, 140.0 ppm. HRMS calcd for C_22_H_13_N_2_ (M + H) 305.1073; found, 305.1071.

##### (*S*)‐[1,1′‐Binaphthalene]‐2,2′‐dicarbonitrile (14(*S*))

**14(*S*)** was prepared in accordance with **14(*R*)** using 15 mmol of **13(*S*)** to afford a white crystalline solid (3.54 g, 77%). *R*_f_ (EtOAc/hexane 25:75) = 0.31. ^1^H NMR (DMSO‐*d*6, 400 MHz): *δ* 7.12 (d, 2H, *J* = 8.4 Hz), 7.56 (dt, 2H, *J* = 6.9, 1.3 Hz), 7.79 (dt, 2H, *J* = 6.9, 1.0 Hz), 8.11 (d, 2H, *J* = 8.6 Hz), 8.27 (d, 2H, *J* = 8.2 Hz), 8.41 (d, 2H, *J* = 8.5 Hz) ppm. ^13^C NMR (DMSO‐*d*6, 100 MHz): *δ* 110.6, 117.4, 125.8, 126.7, 128.9, 129.0, 129.6, 130.8, 131.0, 134.6, 140.0 ppm. HRMS calcd for C_22_H_13_N_2_ (M + H) 305.1073; found, 305.1078.

##### (*R*)‐4,5‐Dihydro‐3*H*‐dinaphtho[2,1‐*c*:1′,2′‐*e*]azepin‐3‐imine (15(*R*))

Under an argon atmosphere, dry and degassed THF (75 mL) was injected into an oven‐dried 500 mL three‐neck round‐bottom flask. The THF was cooled down to ≈2 °C (internal temperature) with an ice‐water bath. Under an increased argon flow LiAlH_4_ (854 mg, 22.5 mmol, 5 equiv) was added portion wise over 6 min. The resulting suspension was stirred for 10 min. **14(*R*)** (1.37 g, 4.5 mmol, 1 equiv) was dissolved in THF (20 mL) in an oven‐dried flask and added to the reaction mixture dropwise over 30 min; the flask was rinsed with THF (2 × 2.5 mL), which was injected as well. The reaction mixture now was green. The ice‐water bath was removed and the reaction mixture was stirred for 3 h at rt, which resulted in the mixture turning red. The reaction mixture was purified with the Fieser workup. Diethylether (115 mL) was added and the mixture was cooled for 10 min in an ice‐water bath. Consecutively, water (1.5 mL), 3 M NaOH (1.5 mL), and more water (4 mL) were added and this mixture was stirred for another 10 min. Celite (5 g) was added and the resulting slurry was filtered using a glass frit. The filter cake was rinsed with EtOAc and the filtrate was dried with Na_2_SO_4_, filtered, and concentrated. The crude product was purified by column chromatography with 25%NH_4_OH/MeOH/CH_2_Cl_2_ 1:9:90 to afford **15(*R*)** (824 mg, 59%) as a white‐yellow solid. *R*_f_ (25%NH_4_OH/MeOH/CH_2_Cl_2_ 1:9:90) = 0.09. ^1^H NMR (DMSO‐*d*6, 400 MHz): *δ* 3.67 (d, 1H, *J* = 11.8 Hz), 4.25 (d, 1H, *J* = 11.8 Hz), 5.76 (s, 1H), 6.38 (br s, 1H), 7.11 (d, 1H, *J* = 8.2 Hz), 7.24 (dt, 1H, *J* = 6.8, 1.3 Hz), 7.29–7.34 (m, 2H), 7.43 (dt, 1H, *J* = 6.7, 1.1 Hz), 7.57 (dt, 1H, *J* = 5.8, 2.1 Hz), 7.67 (d, 1H, *J* = 8.3 Hz), 7.90 (d, 1H, *J* = 8.6 Hz), 8.01 (dd, 2H, *J* = 8.0, 2.2 Hz), 8.09 (d, 1H, *J* = 8.2 Hz), 8.14 (d, 1H, *J* = 8.6 Hz) ppm. ^13^C NMR (DMSO‐*d*6, 100 MHz): *δ* 51.2, 124.6, 124.9, 125.6, 125.9, 126.3, 126.4, 126.7, 127.8, 128.2, 128.3, 128.4, 128.5, 129.9, 130.5, 131.2, 132.0, 132.2, 132.8, 134.5, 144.1, 159.6 ppm. HRMS calcd for C_22_H_17_N_2_ (M + H) 309.1386; found, 309.1381.

##### (*S*)‐4,5‐Dihydro‐3*H*‐dinaphtho[2,1‐*c*:1′,2′‐*e*]azepin‐3‐imine (15(*S*))

**15(*S*)** was prepared in accordance with **15(*R*)** using 2 mmol of **14(*S*)** to afford a white‐yellow solid (432 mg, 70%). *R*_f_ (MeOH/CH_2_Cl_2_ 10:90) = 0.06. ^1^H NMR (DMSO‐*d*6, 400 MHz): *δ* 3.65 (d, 1H, *J* = 11.6 Hz), 4.24 (d, 1H, *J* = 11.7 Hz), 5.76 (s, 1H), 6.23 (br s, 1H), 7.11 (d, 1H, *J* = 8.4 Hz), 7.23 (dt, 1H, *J* = 6.7, 1.2 Hz), 7.29–7.34 (m, 2H), 7.43 (dt, 1H, *J* = 6.8, 1.0 Hz), 7.56 (dt, 1H, *J* = 5.7, 2.3 Hz), 7.66 (d, 1H, *J* = 8.3 Hz), 7.89 (d, 1H, *J* = 8.5 Hz), 8.00 (d, 2H, *J* = 8.0 Hz), 8.08 (d, 1H, *J* = 8.3 Hz), 8.13 (d, 1H, *J* = 8.5 Hz) ppm. ^13^C NMR (DMSO‐*d*6, 100 MHz): *δ* 51.2, 54.9, 124.6, 124.9, 125.6, 125.9, 126.3, 126.4, 126.7, 127.8, 128.2, 128.3, 128.4, 128.5, 129.9, 130.5, 131.2, 132.0, 132.2, 132.7, 134.5, 144.1, 159.6 ppm. HRMS calcd for C_22_H_17_N_2_ (M + H) 309.1386; found, 309.1384.

##### (*R*)‐[1,1′‐Binaphthalene]‐2,2′‐diyldimethanamine (16(*R*))

Under an argon atmosphere, an oven‐dried Schlenk tube containing a magnetic stirrer bar was charged with KBH_4_ (2.59 g, 48 mmol, 16 equiv), Raney Ni (705 mg), and dry EtOH (15 mL). Under stirring, **14(*R*)** (913 mg, 3 mmol, 1 equiv) was added and the resulting mixture was stirred for 22.5 h at 50 °C (external temperature). Then, the reaction mixture was filtered over a pad of celite and the pad was rinsed with EtOH. Next the filtrate was concentrated and the residue dissolved in CH_2_Cl_2_ (100 mL), which was washed with water (3 × 100 mL). After that, the CH_2_Cl_2_ was removed in vacuo and the crude product dissolved in EtOAc (50 mL). This solution was extracted with 1 M HCl (3 × 50 mL). The collected 1 M HCl was neutralized with NaOH and the resulting 200 mL was extracted with diethylether (3 × 200 mL). The organic phase was dried with Na_2_SO_4_, filtered, and concentrated. The crude product was purified by column chromatography with CH_2_Cl_2_ → 25%NH_4_OH/MeOH/CH_2_Cl_2_ 1:9:90 to afford **16(*R*)** (429 mg, 46%) as a yellow solid. *R*_f_ (25%NH_4_OH/MeOH/CH_2_Cl_2_ 1.5:13.5:85) = 0.1. ^1^H NMR (DMSO‐*d*6, 400 MHz): *δ* 3.27 (d, 2H, *J* = 14.1 Hz), 3.37 (d, 2H, *J* = 14.2 Hz), 6.88 (d, 2H, *J* = 8.5 Hz), 7.25 (dt, 2H, *J* = 6.8, 1.2 Hz), 7.45 (dt, 2H, *J* = 6.8, 1.1 Hz), 7.88 (d, 2H, *J* = 8.5 Hz), 8.00 (d, 2H, *J* = 8.0 Hz), 8.07 (d, 2H, *J* = 8.7 Hz) ppm. ^13^C NMR (DMSO‐*d*6, 100 MHz): *δ* 43.2, 125.1, 125.4, 126.3, 126.5, 127.8, 128.0, 132.1, 132.2, 132.6, 139.3 ppm. HRMS calcd for C_22_H_21_N_2_ (M + H) 313.1699; found, 313.1704.

##### (*S*)‐[1,1′‐Binaphthalene]‐2,2′‐diyldimethanamine (16(*S*))

**16(*S*)** was prepared in accordance with **16(*R*)** to afford a yellow solid (218 mg, 23%). *R*_f_ (25%NH_4_OH/MeOH/CH_2_Cl_2_ 1.5:13.5:85) = 0.1. ^1^H NMR (DMSO‐*d*6, 400 MHz): *δ* 3.25 (d, 2H, *J* = 14.0 Hz), 3.40 (d, 2H, *J* = 14.1 Hz), 6.88 (d, 2H, *J* = 8.5 Hz), 7.26 (dt, 2H, *J* = 8.1, 1.0 Hz), 7.46 (t, 2H, *J* = 7.0 Hz), 7.88 (d, 2H, *J* = 8.8 Hz), 8.01 (d, 2H, *J* = 8.0 Hz), 8.07 (d, 2H, *J* = 8.5 Hz) ppm. ^13^C NMR (C*D*Cl_3_, 100 MHz): *δ* 44.3, 126.0, 126.2, 126.7, 128.3, 128.9, 133.0, 133.2, 133.9, 138.7 ppm. HRMS calcd for C_22_H_21_N_2_ (M + H) 313.1699; found, 313.1709.

##### (R)‐[1,1′‐Binaphthalene]‐2,2′‐dicarboxamide (17)

**14(*R*)** (625 mg, 2 mmol, 1 equiv) was dissolved in *i*‐PrOH (100 mL) at 70 °C (external temperature). Now finely crushed NaOH (8 g, 200 mmol, 100 equiv) was added and the mixture stirred for 19.5 h at 70 °C (external temperature). The reaction mixture was concentrated, dissolved in CH_2_Cl_2_ (250 mL), and washed with water (2 × 250 mL). The organic phase was dried with Na_2_SO_4_, filtered, and concentrated. The crude product was purified by column chromatography with EtOAc/toluene 90:10 to obtain **17** (439 mg, 63%) as a white powder. The product was further purified with preparative HPLC (H_2_O/MeCN 30:70) utilizing a Dionex A5890 pump, UVD 170s detector and STH 585 column oven with a VP 250/21 Nucleodur PolarTec, 5 µm column. *R*_f_ (MeOH/CH_2_Cl_2_ 5:95) = 0.33. ^1^H NMR (DMSO‐*d*6, 400 MHz): *δ* 7.00 (d, 2H, *J* = 8.6 Hz), 7.25 (s, 2H), 7.29 (dt, 2H, *J* = 6.8, 1.2 Hz), 7.52 (dt, 2H, *J* = 6.9, 1.0 Hz), 7.71 (d, 2H, *J* = 8.4 Hz), 8.03 (d, 2H, *J* = 8.2 Hz), 8.11 (d, 2H, *J* = 8.4 Hz), 8.38 (s, 2H) ppm. ^13^C NMR (DMSO‐*d*6, 100 MHz): *δ* 124.3, 126.1, 126.7, 126.8. 128.0, 128.2, 131.7, 132.4, 133.5, 135.4, 171.5 ppm. HRMS calcd for C_22_H_16_N_2_NaO_2_ (M + Na) 363.1104; found, 363.1109.

##### Cell Culture

All cancer cell lines were obtained from the German Collection of Microorganisms and Cell Cultures (DSMZ) and cultured in RPMI 1640. The 16HBE14o‐ cell line was kindly provided by the research group of Prof. Dr. Ulrike Garscha and grown in MEM. Both media were supplemented with 10% FBS and 1% penicillin‐streptomycin. All cell lines were cultivated at 37 °C and 5% CO_2_ in 25 cm^2^ or 75 cm^2^ cell culture flasks. The cells were passaged and seeded at ≈80% confluence. For detachment the adherent cells were incubated for 2 min with trypsin‐EDTA.

##### Crystal Violet Assay

Cells were suspended in medium to achieve a concentration of 10,000 or 2500 (LCLC‐103H) cells mL^−^^1^. One hundred microliters of this suspension was added to each well of 96‐well plates. The cells were then incubated for 24 h. One hundred microliters of the respective compound dilution was applied to each well. For the DMSO solvent control (0.1%), rows 1 and 8 of each plate were utilized. The T_0_ plate was left untreated. All plates (except for the T_0_ plate) were then incubated for 24 or 96 h. The same day, the T_0_ plate's media was taken out, and 100 µL of a 1% glutaraldehyde solution in PBS was added to the cells to fix them for 20 min. The fixation solution was removed and 100 µL of PBS was added. Until staining, the T_0_ plate was stored at 4 °C. After 24 or 96 h, the treated cells were fixed in the same way. After removing PBS from every plate, 100 µL of a 0.02% crystal violet solution in distilled water was applied to each well. After 30 min, the crystal violet solution was removed and the plates were placed in a tub full of water for 15 min. Upon removing the water, 150 µL of 70% EtOH was pipetted into each well. Following 2 h of shaking on a microplate shaker, the plates were measured at *λ* = 570 nm using the software Magellan and a TECAN Sunrise absorbance microplate reader. T/C corr (%) values were calculated using Microsoft Excel and GI_50_ values using GraphPad Prism.

##### Cell Cycle Analysis

A2780 cells and SISO cells (1 × 10^6^ in 2 mL media) were seeded into a 6‐well plate. Following a 24 h incubation at 37 °C with 5% CO_2_, the medium was removed and a dilution of **1(*R*)**, **1(*S*)** or DMSO (0.1%) in 3 mL medium was administered. The GI_90_ of **1(*R*)** was used as concentration for both compounds (2.58 µM for A2780 and 2.64 µM for SISO). After 24 h, the medium was removed and the cells incubated with 1 mL trypsin‐EDTA for 5 min. The enzyme reaction was stopped with 1 mL medium and the cells were transferred into 2 mL micro reaction tubes. After centrifuging for 5 min at 500 g, the supernatant was removed. The cells were resuspended in 1 mL medium and centrifuged again; this step was repeated once more. The supernatant was removed again and the cells were fixed with 500 µL ice‐cold 70% EtOH. The samples were either stored on ice for 30 min or at −20 °C till measurement. After centrifuging at 4000 rpm and 4 °C for 10 min, the supernatant was removed once again and the cells treated with 500 µL propidium iodide staining solution (to 3 mL PBS were added 30 µL of a 2.5 mg mL^−1^ propidium iodide solution in distilled water and 30 µL of a 10 mg mL^−1^ ribonuclease A solution in distilled water). The cell suspension was measured with the MACSQuant Analyzer 10 by using the PI channel (*λ*_ex_ = 488 nm, *λ*_em_ = 655–730 nm). The cell population in the FSC and SSC representation was gated to exclude debris and doublets. In a second gating step, the area and height of the PI signal were used to further exclude doublets. Finally, the height of the PI signal was plotted against the cell count to generate cell cycle histograms.

##### Apoptosis Analysis

The Annexin V‐FITC Kit from Miltenyi Biotec (#130‐092‐052) was used to conduct the apoptosis analysis. A2780 and SISO cells (2.5 × 10^5^) were seeded on coverslips placed into 6‐well plates. After 24 h incubation at 37 °C and 5% CO_2_, the medium (2 mL) was removed and the cells were treated with DMSO (0.1%), **1(*R*)**, **1(*S*)**, or Camptothecin (5.00 µM) in medium (3 mL) for 48 h. After treatment, the cells were washed with PBS (2 × 1 mL) and Binding Buffer (1 × 1 mL). Now, the cells were stained with Annexin FITC (25 µL in 500 µL Binding Buffer) for 15 min in the dark. The cells were washed again with 1 mL Binding Buffer and afterward stained with PI (5 µL in 500 µL Binding Buffer) for 15 min in the dark. The staining solution was removed and the coverslips were mounted onto microscopy slides and sealed with nail polish. Images were acquired immediately with the Leica DMi8 fluorescence microscope and the software LAS X.

##### Immunocytochemistry and Fluorescence Microscopy

SISO, MCF‐7, 16HBE14o‐ cells (5 × 10^5^), or LCLC‐103H cells (2.5 × 10^5^) were seeded on coverslips placed into 6‐well plates. After 24 h incubation at 37 °C and 5% CO_2_, the medium (2 mL) was removed and cells treated with a dilution of **1(*R*)**, **1(*S*)**, DMSO (0.1%), paclitaxel, or colchicine in medium (3 mL). After treatment, the cells were washed with PBS (3 × 1 mL), treated with pre‐extraction buffer (0.3% Triton X‐100 with PEM‐Buffer (80 mM PIPES sodium salt, 5 mM EGTA, 2 mM MgCl_2_ adjusted to pH 6.8 with 1 M NaOH)) for 30 s, and afterward immediately fixed for 5 min at −20 °C with MeOH (1 mL). The cells were washed again with PBS (3 × 1 mL), blocked with 500 µL Blocking Buffer (0.3% Triton X‐100 and 5% goat serum in PBS) for 1 h at 37 °C and incubated with 100 µL of the primary antibody diluted in Antibody Dilution Buffer (0.3% Triton X‐100 and 1% BSA in PBS) at 4 °C overnight. The cells were washed with PBS (3 × 1 mL) and incubated for 2 h at rt in the dark with 100 µL of the secondary antibody diluted in Antibody Dilution Buffer. After washing with PBS (3 × 1 mL), the cells were treated with a DAPI solution (1 mL, 0.5 µg mL^−1^ in PBS) for 20 min at rt in the dark. Finally, the coverslips were washed with PBS (2 × 1 mL) and sterile, filtered water (1 × 1 mL), mounted onto microscopy slides, and sealed with nail polish. Images were acquired with the Leica DMi8 fluorescence microscope and the software LAS X. For each antibody staining represented in the figures, all images were taken on the same day. The experiments were conducted at least three times for DMSO, **1(*R*)**, and **1(*S*)**, and at least twice for colchicine and paclitaxel. The only image processing performed was brightness adjustment of individual channels in LAS X. All images of the same staining type were subjected to the same brightness adjustment, with the exception of those acquired from gamma‐tubulin or centrin staining, where each image was individually adjusted (without changing the trend) to allow the visualization of all structures.

Antibodies used were as follows: Alexa Fluor 488 conjugate (#4412, CST, 1:500), Alexa Fluor 594 conjugate (#8890, CST, 1:500), alpha‐tubulin (#11224‐1‐AP, Proteintech, 1:100), acetyl alpha‐tubulin (#5335, CST, 1:100), centrin (#ZMS1054, Sigma–Aldrich, 1:100), detyrosinated alpha‐tubulin (#abx375026, Abbexa, 1:100), EB1 (#17717‐1‐AP, Proteintech, 1:200), Eg5 (#23333‐1‐AP, Proteintech, 1:200), gamma‐tubulin (#CL594‐66320, Proteintech, 1:100), KIF1C (#12760‐1‐AP, Proteintech, 1:100), stathmin 1 (#11157‐1‐AP, Proteintech, 1:100).

##### Tubulin Polymerization Assay

To measure the effects of BINAM on the polymerization of tubulin, we used a commercial fluorescence‐based Tubulin Polymerization Assay Kit (#BK011P, Cytoskeleton, Inc.). The assay was performed at 37 °C according to the manufacturer's standard protocol; conditions were 2 mg mL^−1^ tubulin, PEM‐Buffer (80 mM PIPES pH 6.9, 0.5 mM EGTA, 2.0 mM MgCl_2_), 1.0 mM GTP, and 15% glycerol. Ten‐fold stock solutions of DMSO, **1(*R*)**, **1(*S*)**, paclitaxel, and colchicine (the final DMSO‐concentration after dilution was 0.5%) were prepared. Next, tubulin, PEM‐Buffer, GTP, and glycerol (tubulin reaction mix) were combined. 5 µL of the respective 10x stock solution was pipetted into a black flat‐bottom 96‐well plate. To each well, 50 µL of the tubulin reaction mix was added and the fluorescence was measured every minute for 1 h. Excitation was set to *λ* = 360 nm and emission to *λ* = 450 nm on a Tecan Infinite M200 Pro. The software used for measurement was Tecan i‐control.

##### Kinase Screening

Kinase Screening was performed at Reaction Biology (Freiburg, Germany) by using the ^33^PanQinase Assay.^[^ ^90^ ^]^

## Conflict of Interest

The authors declare no conflict of interest.

## Author Contributions

**Malte Eichelbaum**: conceptualization (equal); data curation (lead); formal analysis (lead); investigation (lead); methodology (lead); validation (lead); visualization (Lead); writing—original draft (lead), writing—review & editing (equal). **Patrick Bednarski**: conceptualization: (equal); funding acquisition (lead); project administration (lead); resources (lead); software (lead); supervision (lead); writing—review & editing (equal). All authors have given approval to the final version of the manuscript.

## Supporting information

Supplementary Material

## Data Availability

The data that support the findings of this study are available from the corresponding author upon reasonable request.
