# Supplementary material for: Cytotoxicity of Atropisomeric [1,1′‐Binaphthalene]‐2,2′‐Diamines (BINAM) and Analogs in Human Cancer Cells: Enantioselectivity, Structure–Activity Relationships, and Mechanism
Source: ChemMedChem. 2025 Aug 27;20(19):e202500426. doi: 10.1002/cmdc.202500426 (PMC12503911; doi:10.1002/cmdc.202500426)

Supporting Information

Cytotoxicity of Atropisomeric [1,1'-Binaphthalene]-2,2'-diamines (BINAM) and Analogues in Human Cancer Cells: Enantioselectivity, Structure-activity Relationships and Mechanism

Malte Eichelbaum and Patrick J. Bednarski

Institut für Pharmazie, Universität Greifswald, Friedrich-Ludwig-Jahn-Straße 17, 17489 Greifswald

Contents of SI

Dihedral angles of active binaphthyls....S3

HPLC chromatograms....S4

CD spectra....S26

NMR spectra....S33

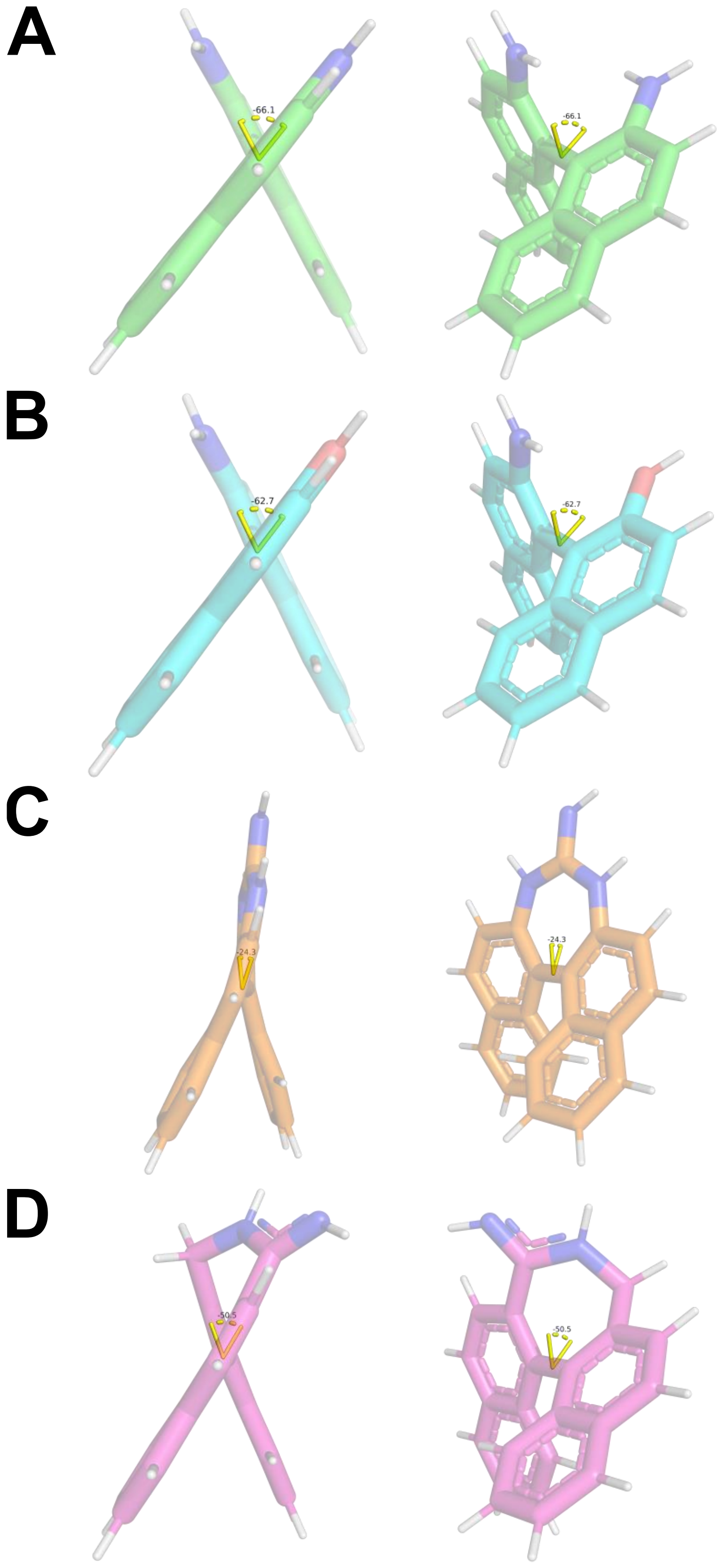

Figure S1. Three-dimensional representation of the active binaphthyls and their torsion angles between 2 and 2′ after optimization using the UFF in Avogadro. (A) Compound **1(*R*)**: 66,1 °. (B) Compound **11(*R*)**: 62,7 °. (C) Compound **12(*R*)**: 24,3 °. (D) Compound **15(*R*)**: 50,5 °

HPLC chromatograms

Compound 2:

Column: Nucleodur® PolarTec (5 µm, 4.6×250 mm)
Eluent: 25 mM Phosphate Buffer pH 3/MeCN 50:50
Flowrate: 0.7 mL/min

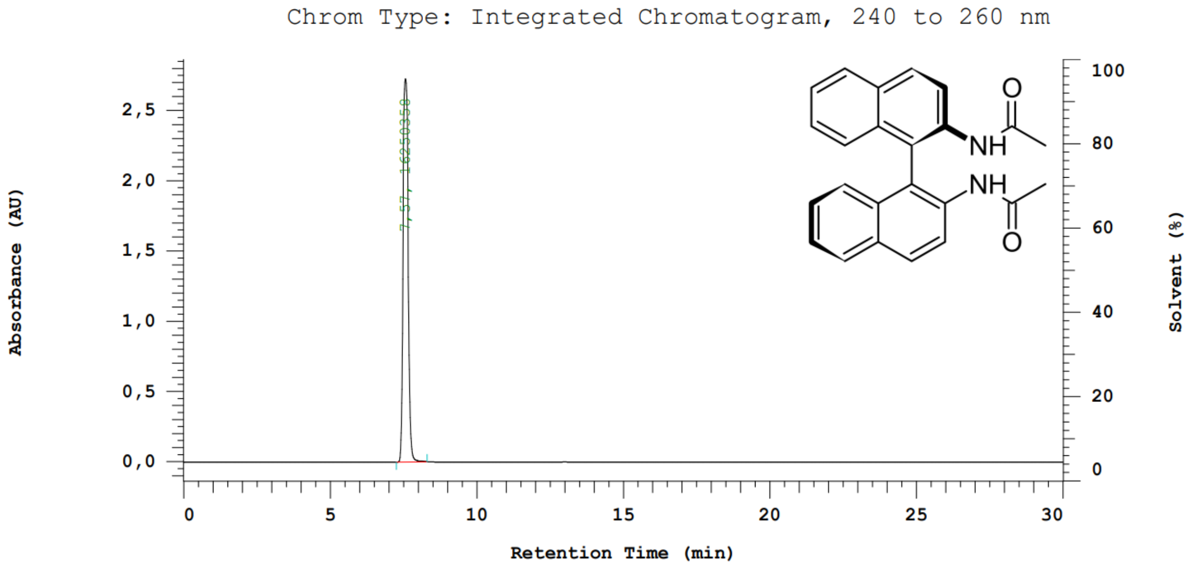

No.	RT	Area	Area %	BC	R-Factor	Purity	Height
1	7,57	16250358	100,000	BB		0,9999	1363588
		16250358	100,000				1363588

Column: Chiralcel® OD-H (5 µm, 4.6×250 mm)
Eluent: *i*-PrOH/Hexane 10:90
Flowrate: 1 mL/min
Wavelength: 280 nm

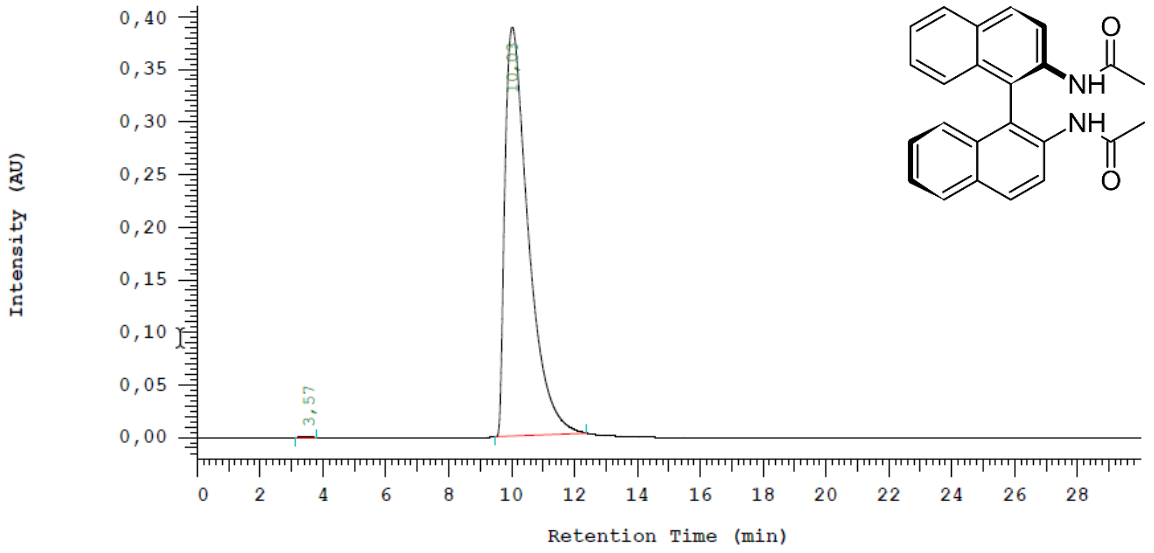

No.	RT	Area	Area %	BC	R-Factor
1	3,57	5627	0,056	BB	
2	10,03	10006484	99,944	BB	
		10012111	100,000		

Compound 3:

Column: Nucleodur® PolarTec (5 µm, 4.6×250 mm)
Eluent: 25 mM Phosphate Buffer pH 3/MeCN 50:50
Flowrate: 0.7 mL/min

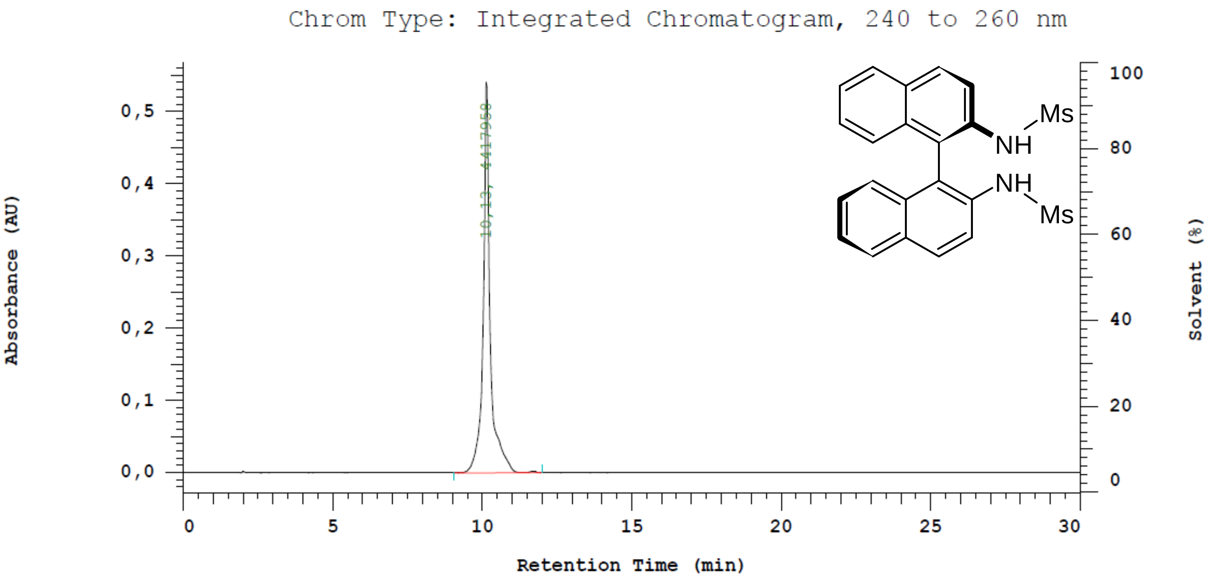

No.	RT	Area	Area %	BC	R-Factor	Purity	Height
1	10,13	4417958	100,000	BB		0,9999	270105
		4417958	100,000				270105

Column: Chiralcel® OD-H (5 µm, 4.6×250 mm)
Eluent: *i*-PrOH/Hexane/TEA 30:70:0.1
Flowrate: 1 mL/min
Wavelength: 280 nm

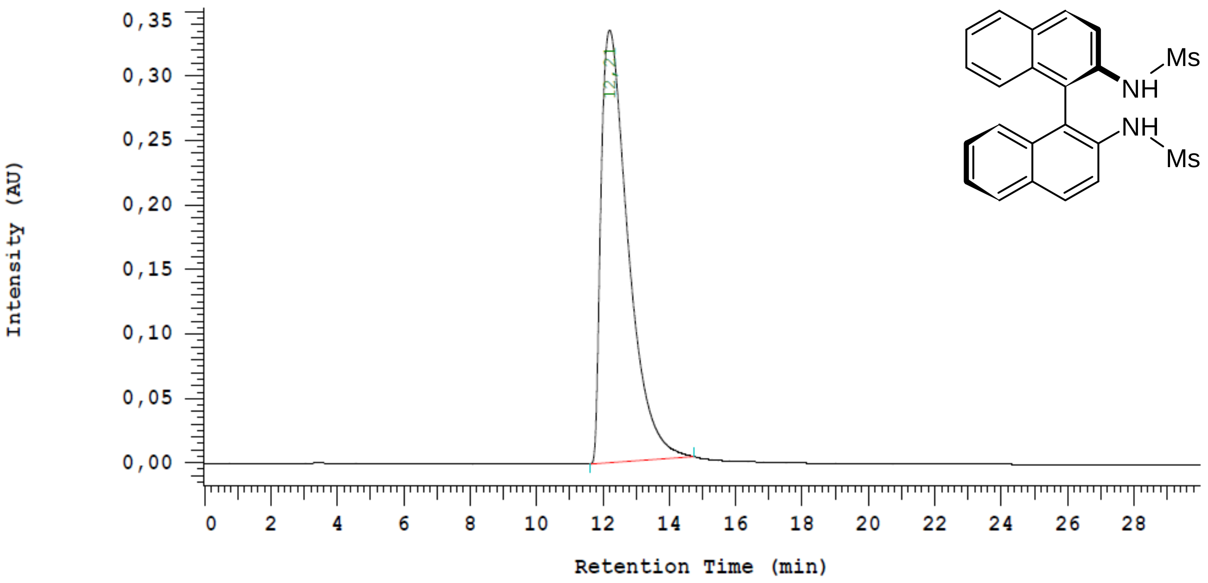

No.	RT	Area	Area %	BC	R-Factor
1	12,21	9494281	100,000	BB	
		9494281	100,000		

Compound 4:
Column: Nucleodur® PolarTec (5 µm, 4.6×250 mm)
Eluent: 25 mM Phosphate Buffer pH 3/MeCN 40:60
Flowrate: 0.7 mL/min

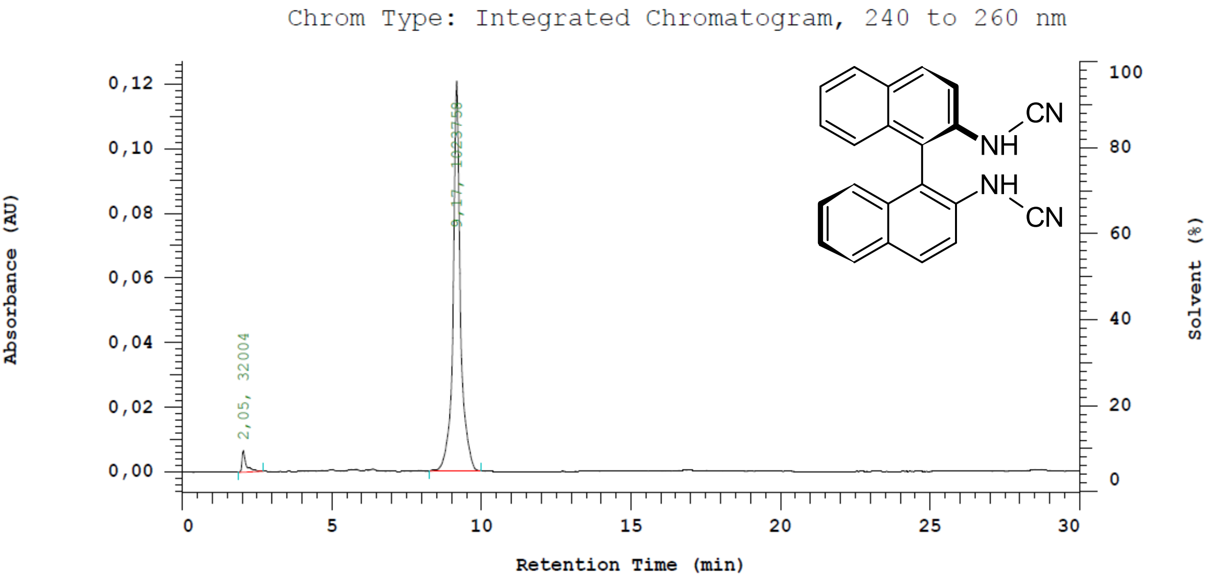

No.	RT	Area	Area %	BC	R-Factor	Purity	Height
1	2,05	32004	3,031	BB		0,9962	3342
2	9,17	1023758	96,969	BB		0,9999	60173
		1055762	100,000				63515

Column: Chiralcel® OD-H (5 µm, 4.6×250 mm)
Eluent: *i*-PrOH/Hexane 20:80
Flowrate: 1 mL/min
Wavelength: 280 nm

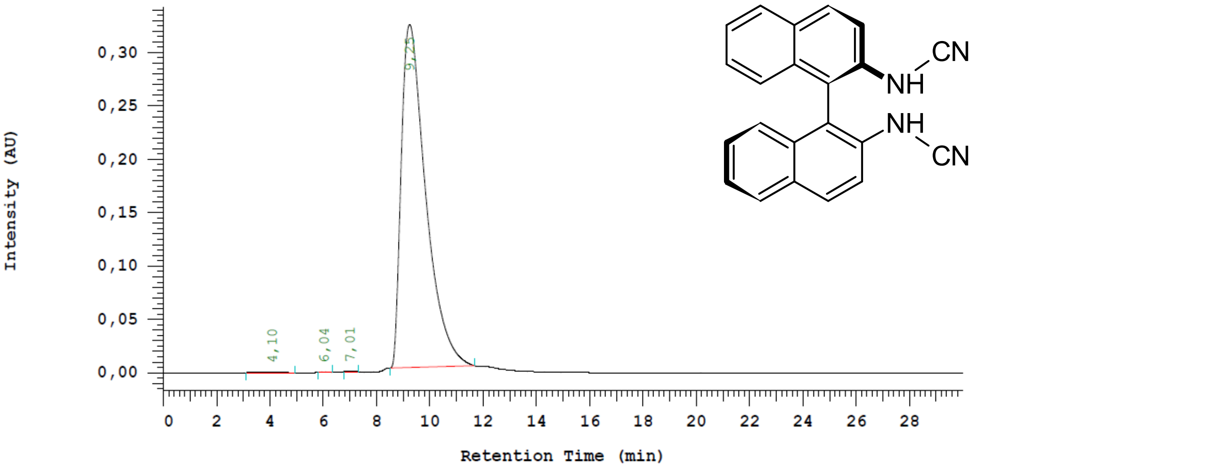

No.	RT	Area	Area %	BC	R-Factor
1	4,10	13505	0,132	BB	
2	6,04	2783	0,027	BB	
3	7,01	6858	0,067	BB	
4	9,25	10183864	99,773	BB	
		10207010	100,000		

Compound 5:
Column: Nucleodur® PolarTec (5 µm, 4.6×250 mm)
Eluent: 25 mM Phosphate Buffer pH 3/MeCN 20:80
Flowrate: 0.7 mL/min

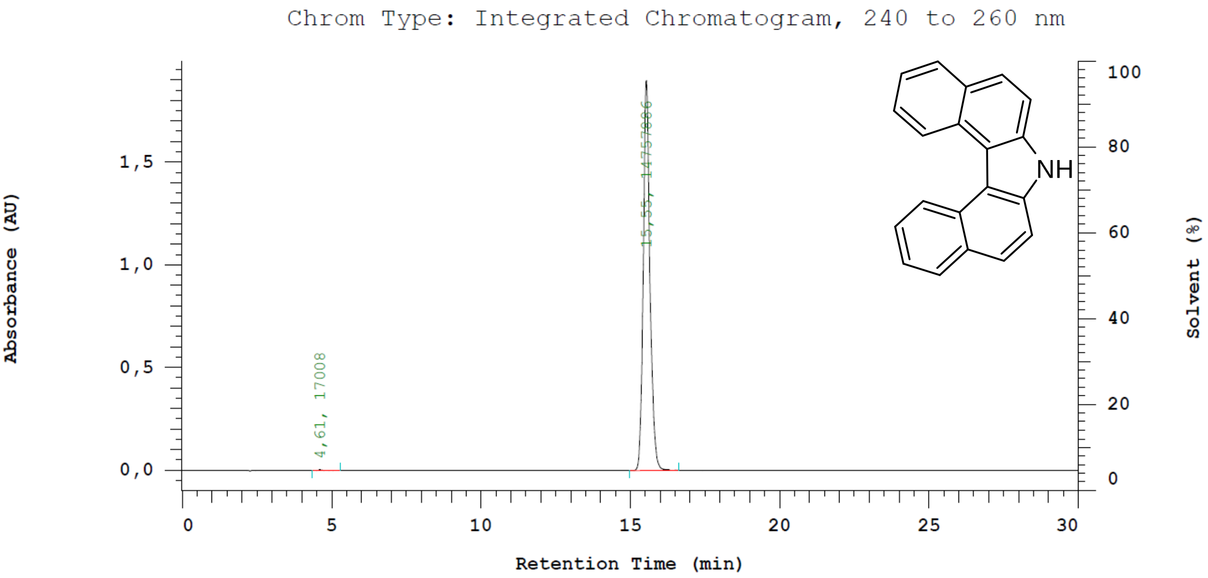

No.	RT	Area	Area %	BC	R-Factor	Purity	Height
1	4,61	17008	0,115	BB		0,6104	2037
2	15,55	14757886	99,885	BB		0,9999	948382
		14774894	100,000				950419

Compound 6:
Column: Nucleodur® PolarTec (5 µm, 4.6×250 mm)
Eluent: 25 mM Phosphate Buffer pH 3/MeCN 30:70
Flowrate: 0.7 mL/min

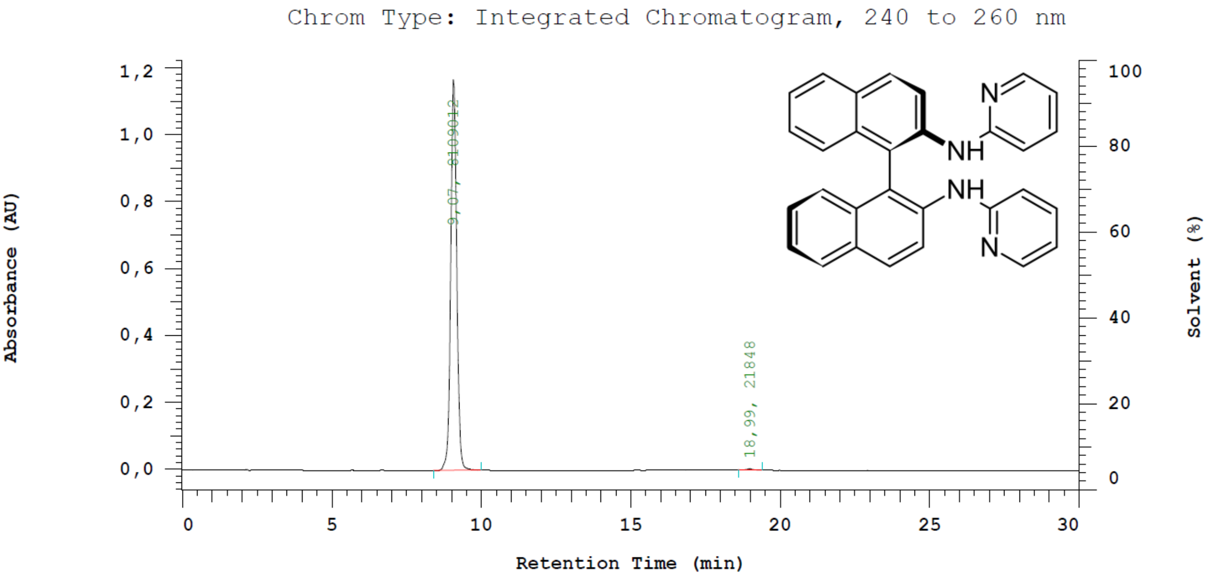

No.	RT	Area	Area %	BC	R-Factor	Purity	Height
1	9,07	8109012	99,731	BB		0,9995	582999
2	18,99	21848	0,269	BB		0,6532	1899
		8130860	100,000				584898

Column: Chiralcel® OD-H (5 µm, 4.6×250 mm)
Eluent: *i*-PrOH/Hexane 10:90
Flowrate: 1 mL/min
Wavelength: 280 nm

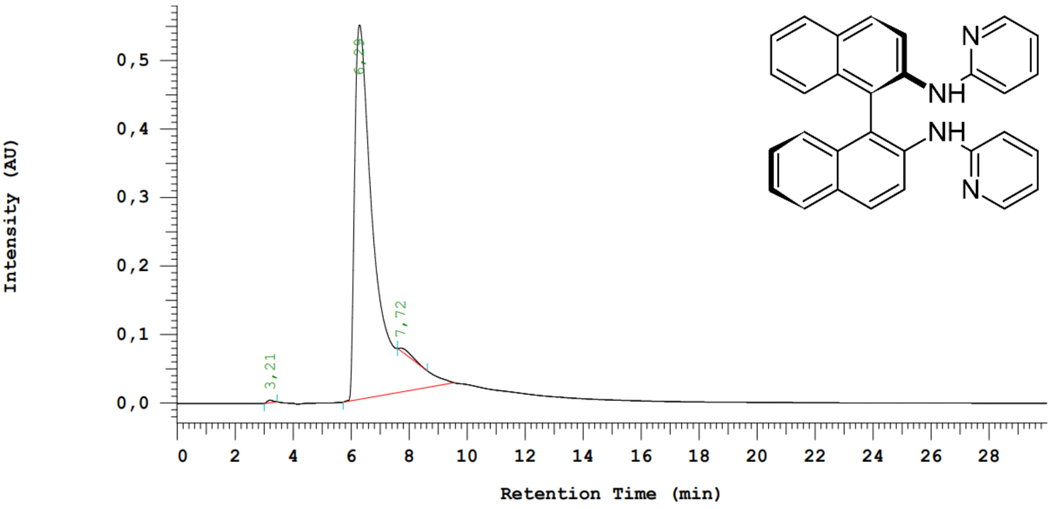

No.	RT	Area	Area %	BC	R-Factor
1	3,21	24507	0,183	BB	
2	6,29	13304950	99,084	BV	
3	7,72	98437	0,733	TBB	
		13427894	100,000		

Compound 7:

Column: Nucleodur® PolarTec (5 µm, 4.6×250 mm)
Eluent: 25 mM Phosphate Buffer pH 3/MeCN 30:70
Flowrate: 0.7 mL/min

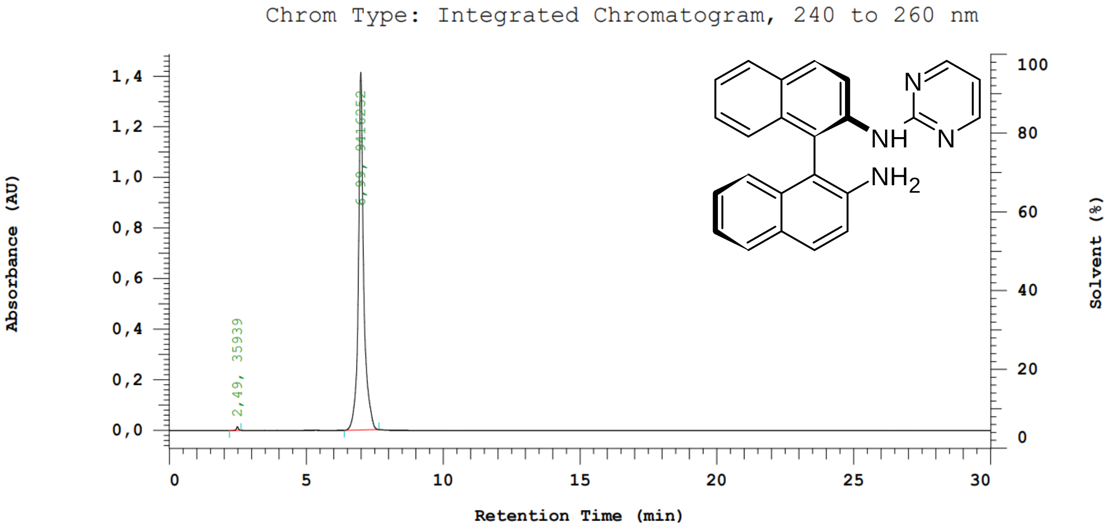

No.	RT	Area	Area %	BC	R-Factor	Purity	Height
1	2,49	35939	0,380	BB		0,9651	7408
2	6,99	9416252	99,620	BB		1,0000	706822
		9452191	100,000				714230

Column: Chiralcel® OD-H (5 µm, 4.6×250 mm)
Eluent: *i*-PrOH/Hexane 10:90
Flowrate: 1 mL/min
Wavelength: 280 nm

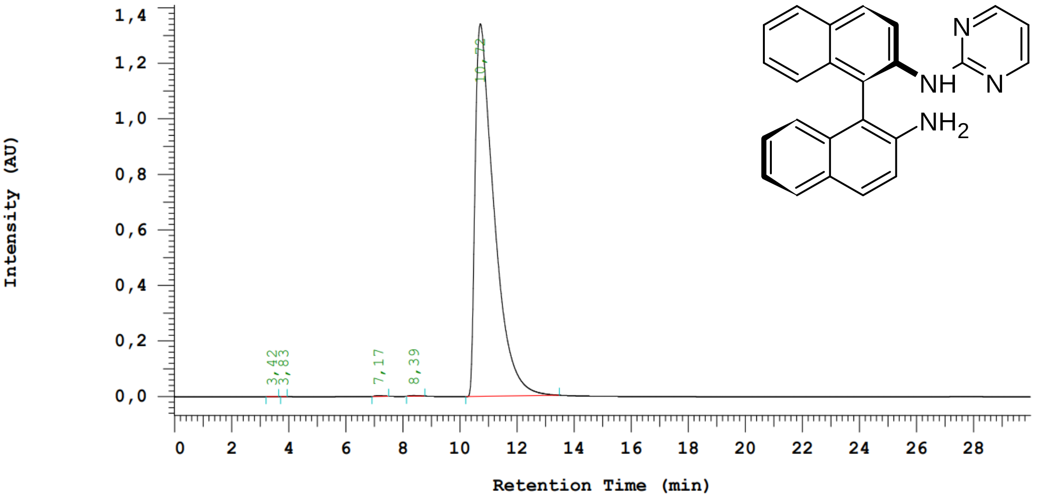

No.	RT	Area	Area %	BC	R-Factor
1	3,42	4281	0,014	BB	
2	3,83	1625	0,005	BB	
3	7,17	23516	0,076	BB	
4	8,39	25133	0,081	BB	
5	10,72	30826720	99,823	BB	
		30881275	100,000		

Compound 8:

Column: Nucleodur® PolarTec (5 µm, 4.6×250 mm)
Eluent: 25 mM Phosphate Buffer pH 3/MeCN 30:70
Flowrate: 0.7 mL/min

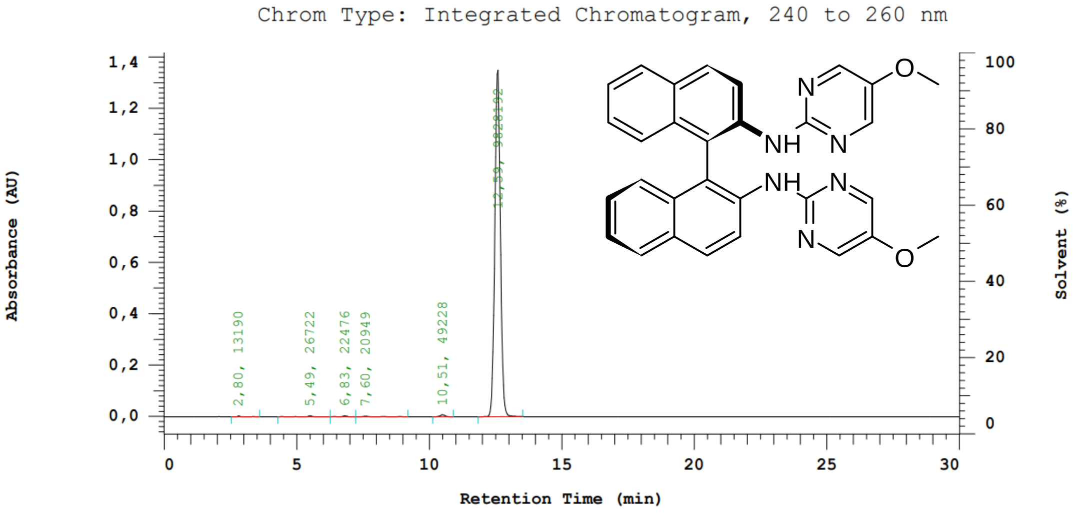

No.	RT	Area	Area %	BC	R-Factor	Purity	Height
1	2,80	13190	0,132	BB		0,0317	1879
2	5,49	26722	0,268	BB		0,9819	2004
3	6,83	22476	0,226	BB		0,7851	1881
4	7,60	20949	0,210	BB		0,9793	1018
5	10,51	49228	0,494	BB		0,9986	3848
6	12,59	9828192	98,669	BB		0,9996	674700
		9960757	100,000				685330

Column: Chiralcel® OD-H (5 µm, 4.6×250 mm)
Eluent: *i*-PrOH/Hexane 10:90
Flowrate: 1 mL/min
Wavelength: 280 nm

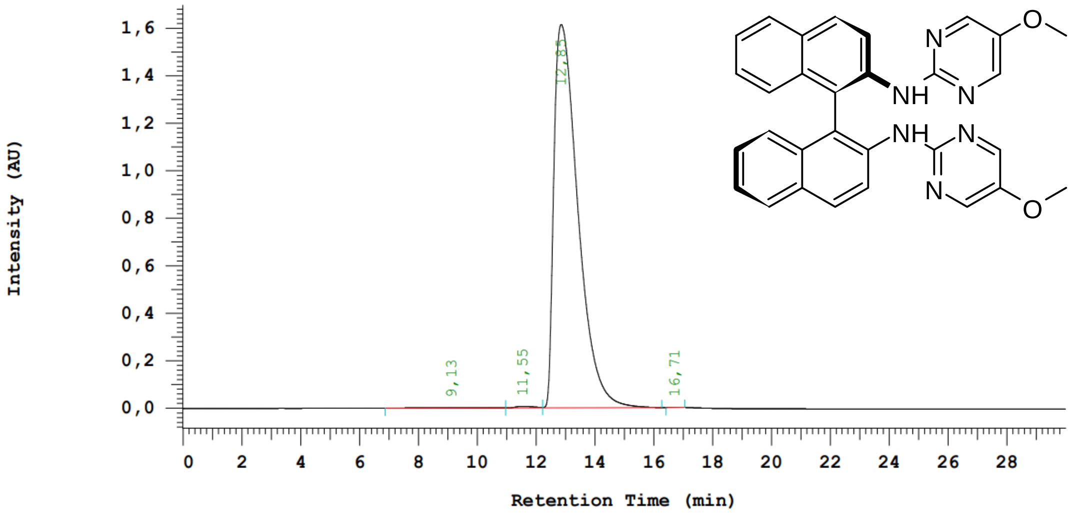

No.	RT	Area	Area %	BC	R-Factor
1	9,13	47607	0,104	BB	
2	11,55	142716	0,311	BV	
3	12,85	45636873	99,558	VB	
4	16,71	12440	0,027	BB	
		45839636	100,000		

Compound 9:

Column: Nucleodur® PolarTec (5 µm, 4.6×250 mm)
Eluent: 25 mM Phosphate Buffer pH 3/MeCN 30:70
Flowrate: 0.7 mL/min

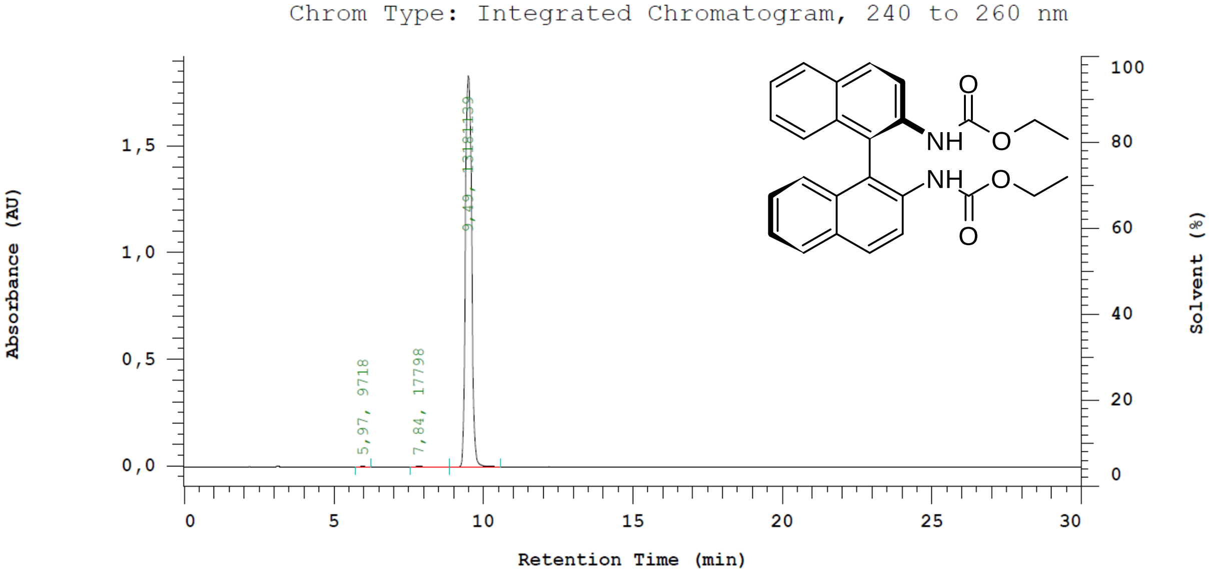

No.	RT	Area	Area %	BC	R-Factor	Purity	Height
1	5,97	9718	0,074	BB		0,9976	1135
2	7,84	17798	0,135	BB		0,9828	1643
3	9,49	13181139	99,792	BB		0,9997	915514
		13208655	100,000				918292

Column: Chiralcel® OD-H (5 µm, 4.6×250 mm)
Eluent: *i*-PrOH/Hexane 5:95
Flowrate: 1 mL/min
Wavelength: 280 nm

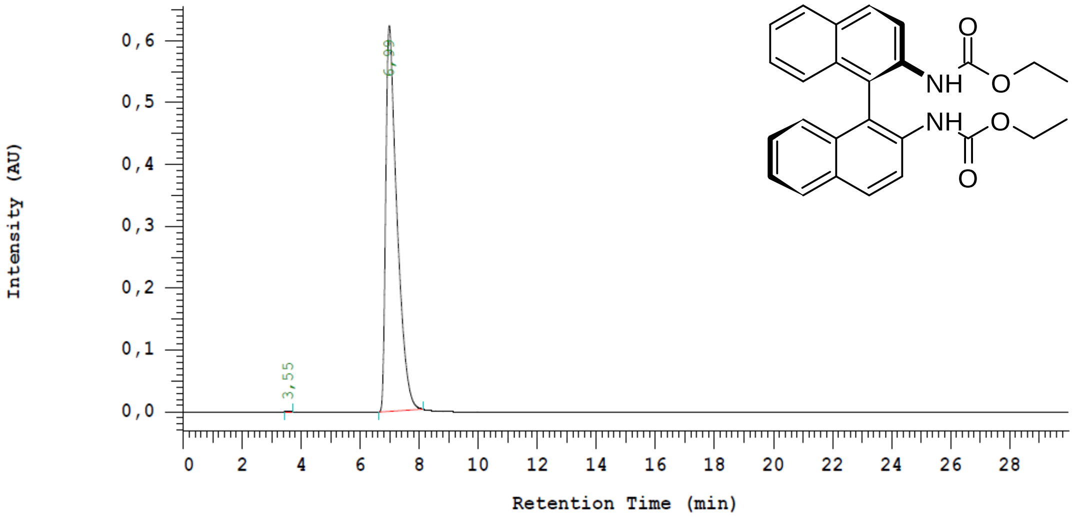

No.	RT	Area	Area %	BC	R-Factor
1	3,55	3700	0,046	BB	
2	6,99	8128172	99,954	BB	
		8131872	100,000		

Compound 10:
Column: Nucleodur® PolarTec (5 µm, 4.6×250 mm)
Eluent: 25 mM Phosphate Buffer pH 3/MeCN 30:70
Flowrate: 0.7 mL/min

Chrom Type: Integrated Chromatogram, 240 to 260 nm

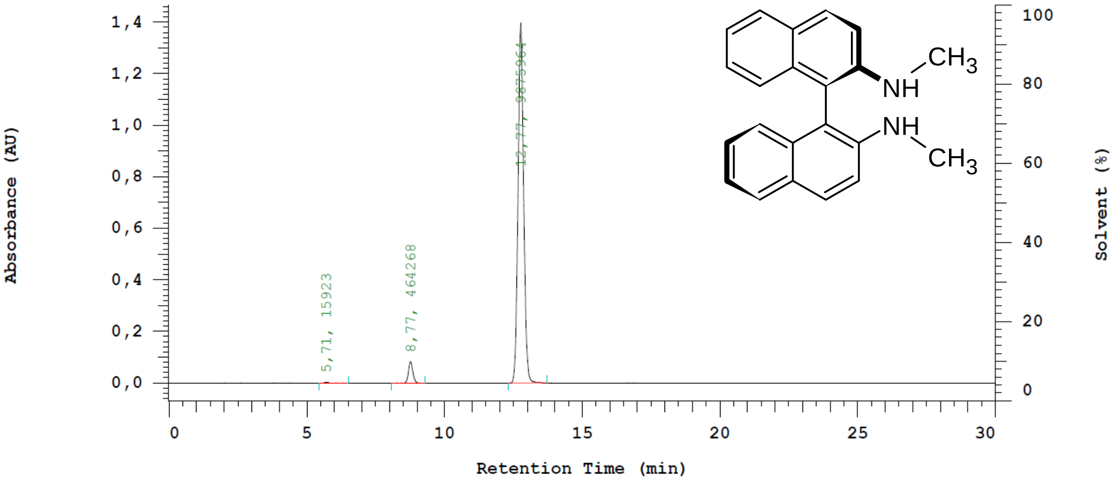

No.	RT	Area	Area %	BC	R-Factor	Purity	Height
1	5,71	15923	0,154	BB		0,9302	1695
2	8,77	464268	4,483	BB		0,9996	41370
3	12,77	9875964	95,363	BB		0,9998	698645
		10356155	100,000				741710

Column: Chiralcel® OD-H (5 µm, 4.6×250 mm)
Eluent: *i*-PrOH/Hexane 5:95
Flowrate: 1 mL/min
Wavelength: 280 nm

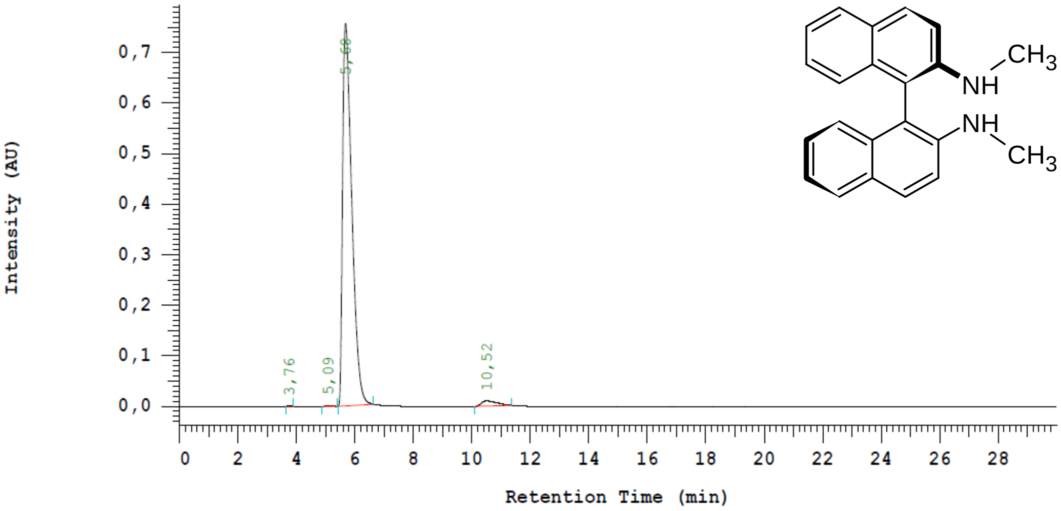

No.	RT	Area	Area %	BC	R-Factor
1	3,76	3342	0,040	BB	
2	5,09	13567	0,163	BB	
3	5,68	8106276	97,590	BB	
4	10,52	183305	2,207	BB	
		8306490	100,000		

Compound 11(*R*):

Column: Nucleodur® PolarTec (5 µm, 4.6×250 mm)

Eluent: 25 mM Phosphate Buffer pH 3/MeCN 35:65

Flowrate: 0.7 mL/min

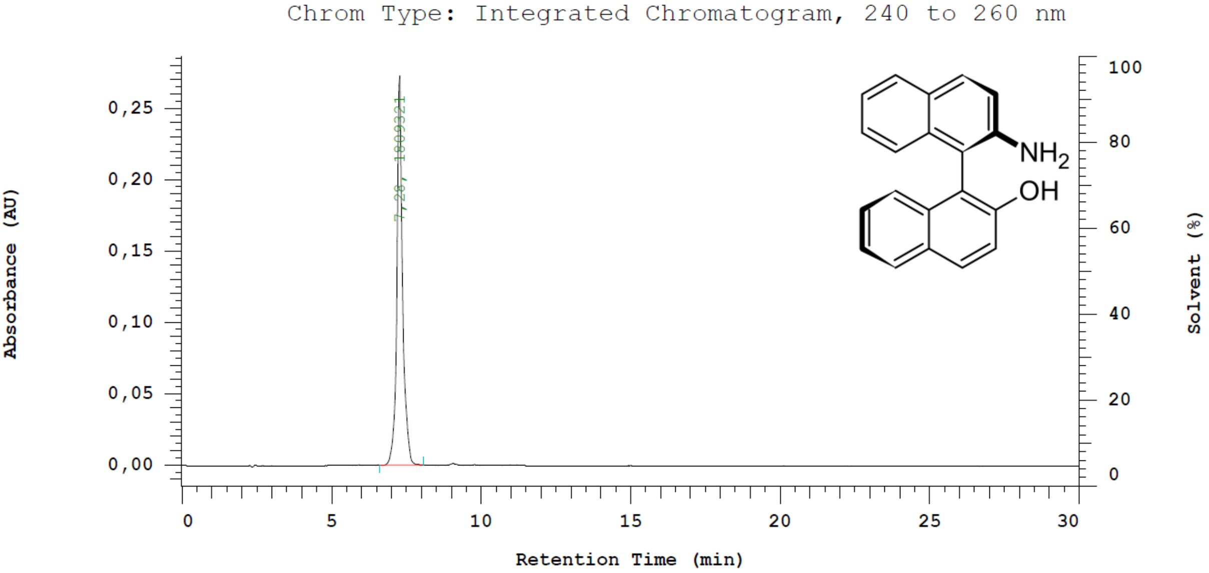

No.	RT	Area	Area %	BC	R-Factor	Purity	Height
1	7,28	1809321	100,000	BB		0,9998	136340
		1809321	100,000				136340

Column: Chiralcel® OD-H (5 µm, 4.6×250 mm)

Eluent: *i*-PrOH/Hexane 10:90

Flowrate: 1 mL/min

Wavelength: 280 nm

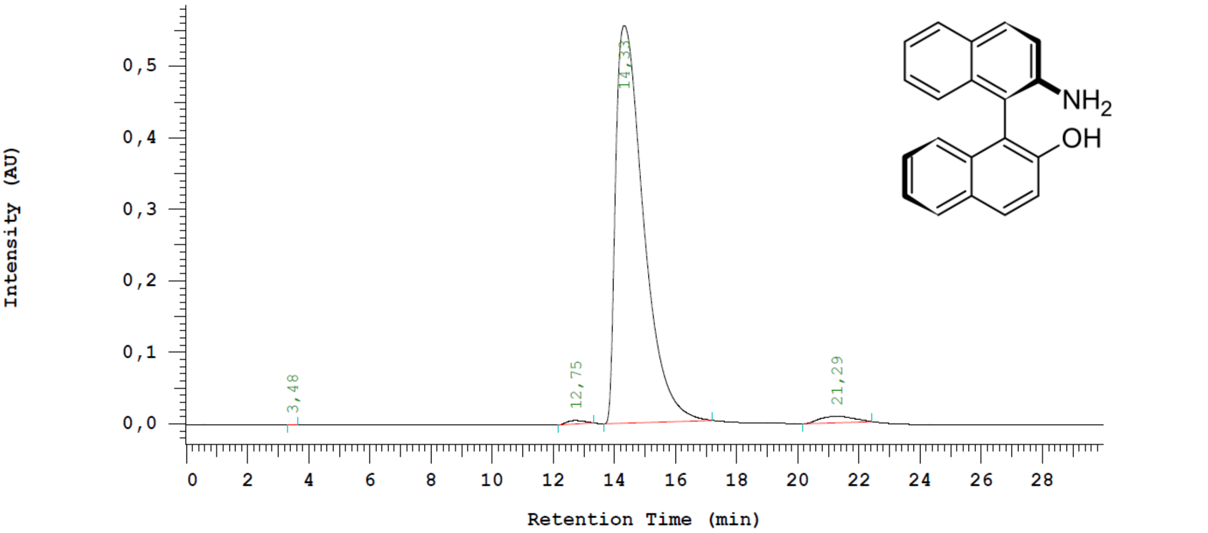

No.	RT	Area	Area %	BC	R-Factor
1	3,48	1579	0,009	BB	
2	12,75	91949	0,511	BB	
3	14,33	17570556	97,574	BB	
4	21,29	343404	1,907	BB	
		18007488	100,000		

Compound 11(*S*):
Column: Nucleodur® PolarTec (5 µm, 4.6×250 mm)
Eluent: 25 mM Phosphate Buffer pH 3/MeCN 35:65
Flowrate: 0.7 mL/min

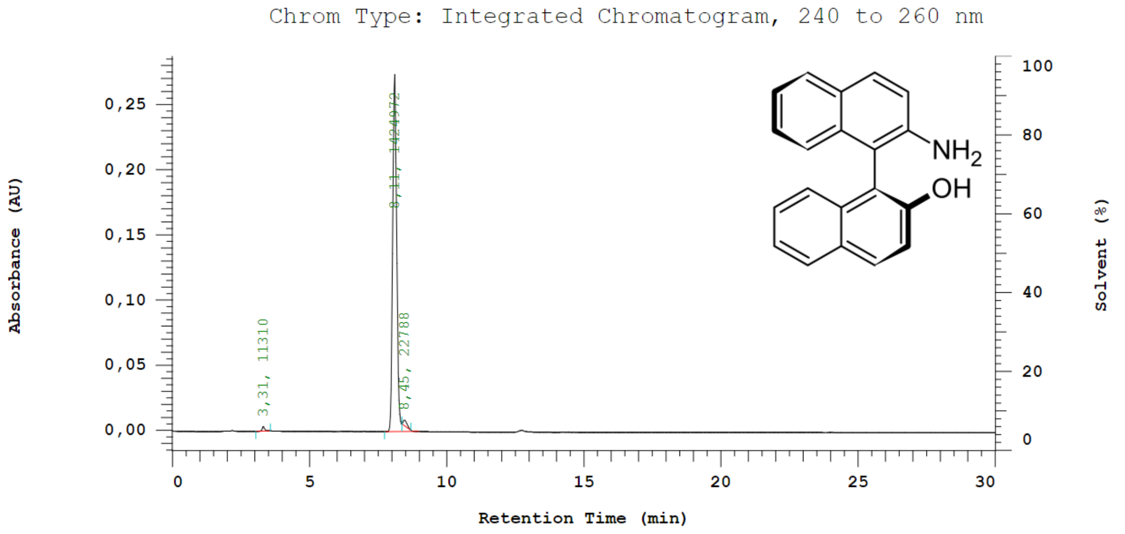

No.	RT	Area	Area %	BC	R-Factor	Purity	Height
1	3,31	11310	0,775	BB		0,9747	1870
2	8,11	1424972	97,663	BV		0,9993	137040
3	8,45	22788	1,562	TBB		0,9204	2096
		1459070	100,000				141006

Column: Chiralcel® OD-H (5 µm, 4.6×250 mm)
Eluent: *i*-PrOH/Hexane 10:90
Flowrate: 1 mL/min
Wavelength: 280 nm

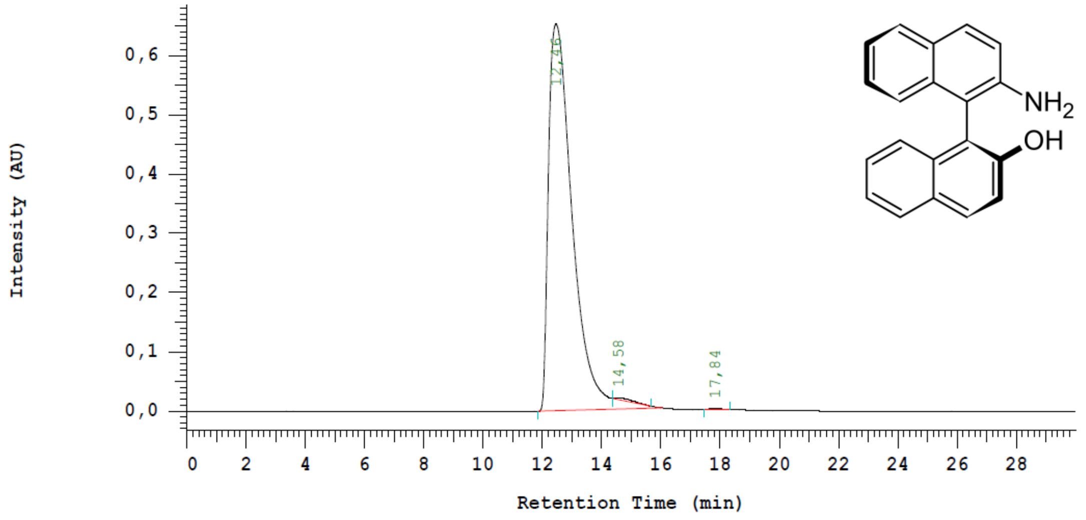

No.	RT	Area	Area %	BC	R-Factor
1	12,46	18320795	99,502	BV	
2	14,58	75825	0,412	TBB	
3	17,84	15801	0,086	BB	
		18412421	100,000		

Manually prepared mixture of compound 11(*R*) and 11(*S*):
Column: Chiralcel® OD-H (5 µm, 4.6×250 mm)
Eluent: *i*-PrOH/Hexane 10:90
Flowrate: 1 mL/min
Wavelength: 280 nm

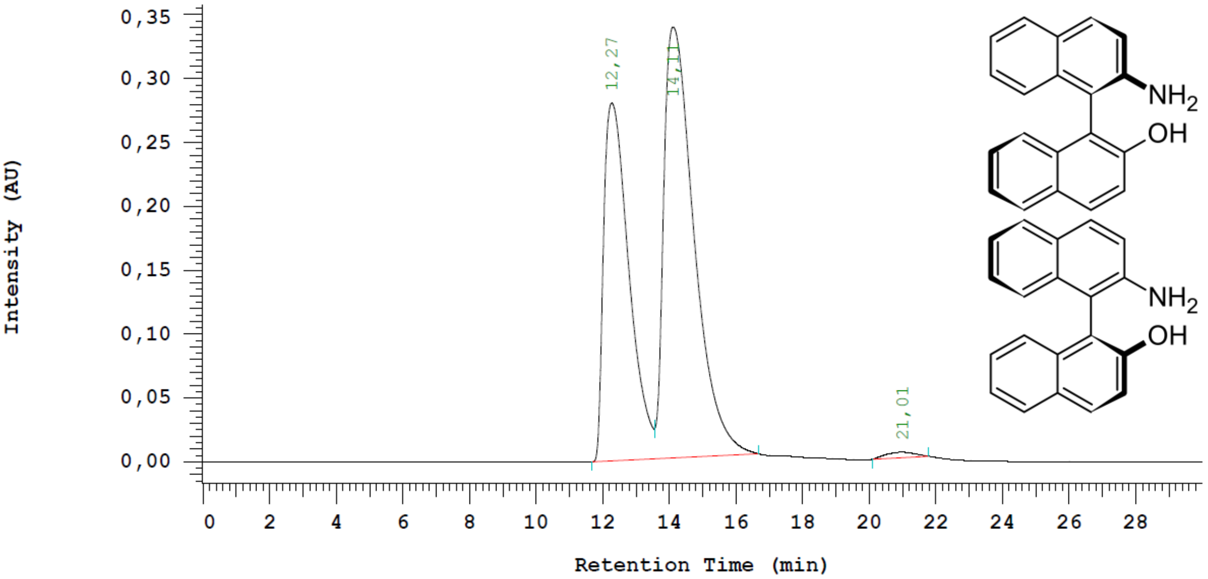

No.	RT	Area	Area %	BC	R-Factor
1	12,27	7349733	40,472	BV	
2	14,11	10678373	58,802	VB	
3	21,01	131871	0,726	BB	
		18159977	100,000		

Compound 12(*R*):

Column: Nucleodur® C18 HTec (5 µm, 4.6×250 mm)
Eluent: 25 mM Acetate Buffer pH 5/MeCN 60:40
Flowrate: 0.7 mL/min

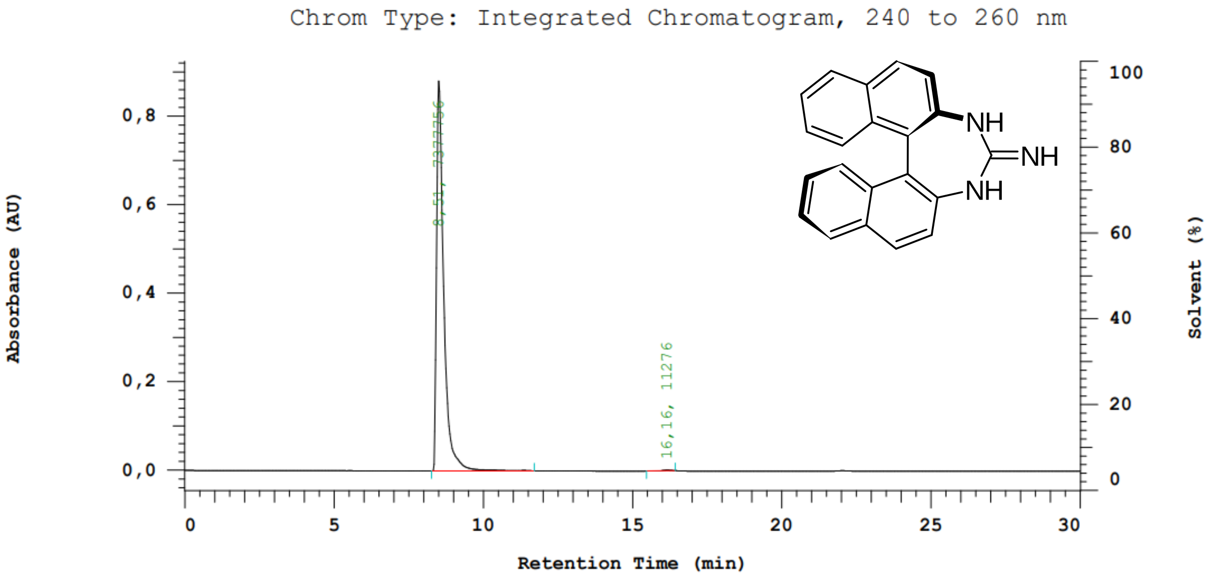

No.	RT	Area	Area %	BC	R-Factor	Purity	Height
1	8,51	7377756	99,847	BB		0,9997	440379
2	16,16	11276	0,153	BB		0,7593	725
		7389032	100,000				441104

On the column Chiralcel® OD-H (5 µm, 4.6×250 mm) compound **12(*R*)** caused extreme band broadening.

Compound 12(*S*):

Column: Nucleodur® C18 HTec (5 µm, 4.6×250 mm)
Eluent: 25 mM Acetate Buffer pH 5/MeCN 70:30
Flowrate: 0.7 mL/min

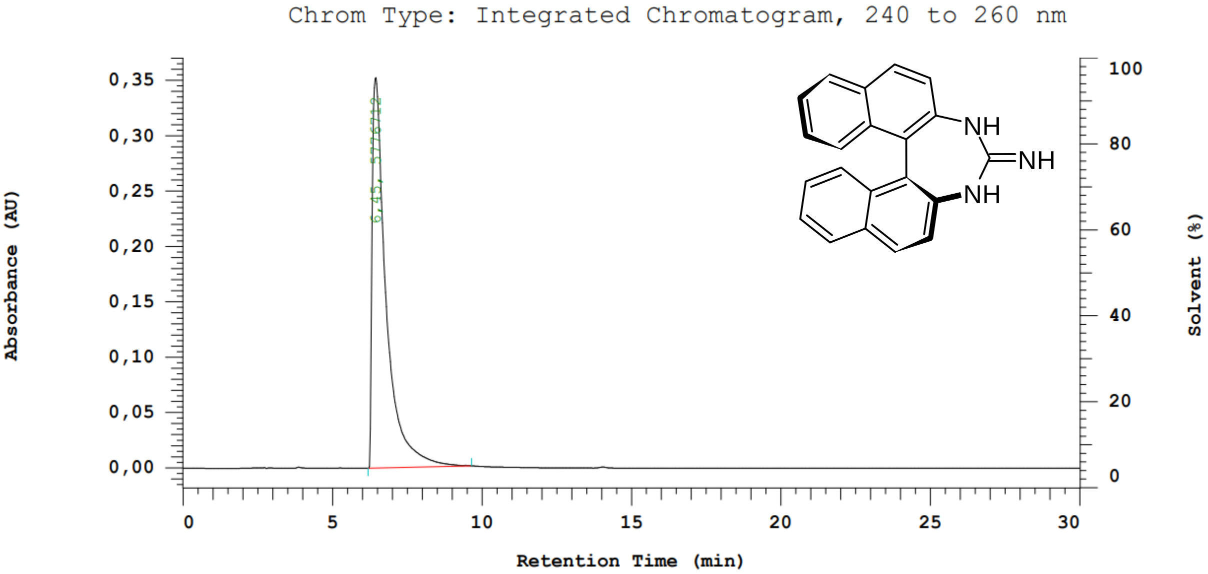

No.	RT	Area	Area %	BC	R-Factor	Purity	Height
1	6,45	5776712	100,000	BB		0,9997	176249
		5776712	100,000				176249

On the column Chiralcel® OD-H (5 µm, 4.6×250 mm) compound **12(*S*)** caused extreme band broadening.

Compound 14(*R*):
Column: Nucleodur® PolarTec (5 µm, 4.6×250 mm)
Eluent: 25 mM Phosphate Buffer pH 3/MeCN 40:60
Flowrate: 0.7 mL/min

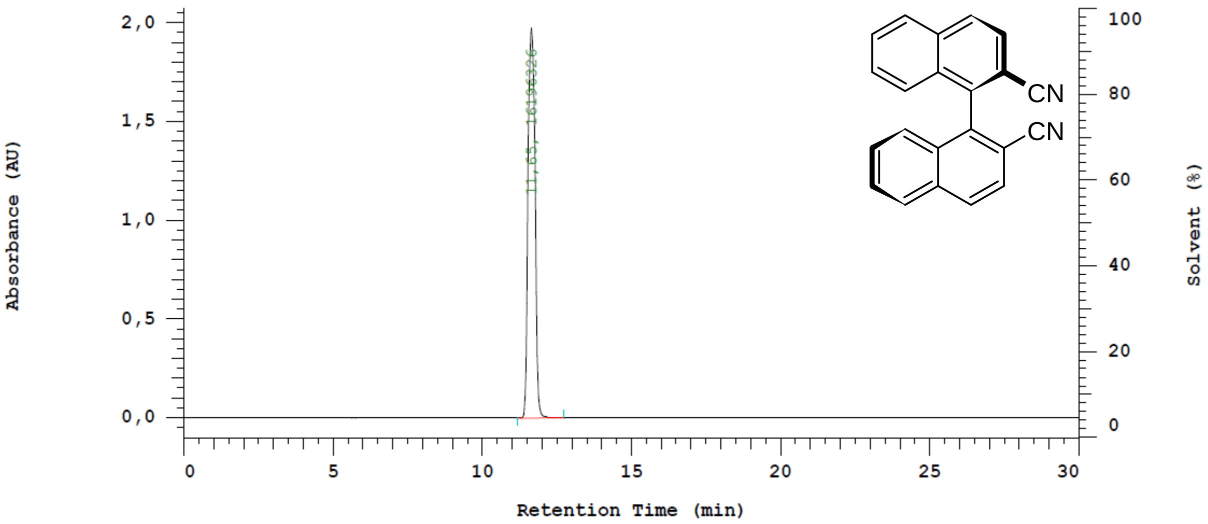

No.	RT	Area	Area %	BC	R-Factor	Purity	Height
1	11,65	16196326	100,000	BB		0,9995	986512
		16196326	100,000				986512

Column: Chiralcel® OD-H (5 µm, 4.6×250 mm)
Eluent: *i*-PrOH/Hexane 10:90
Flowrate: 1 mL/min
Wavelength: 280 nm

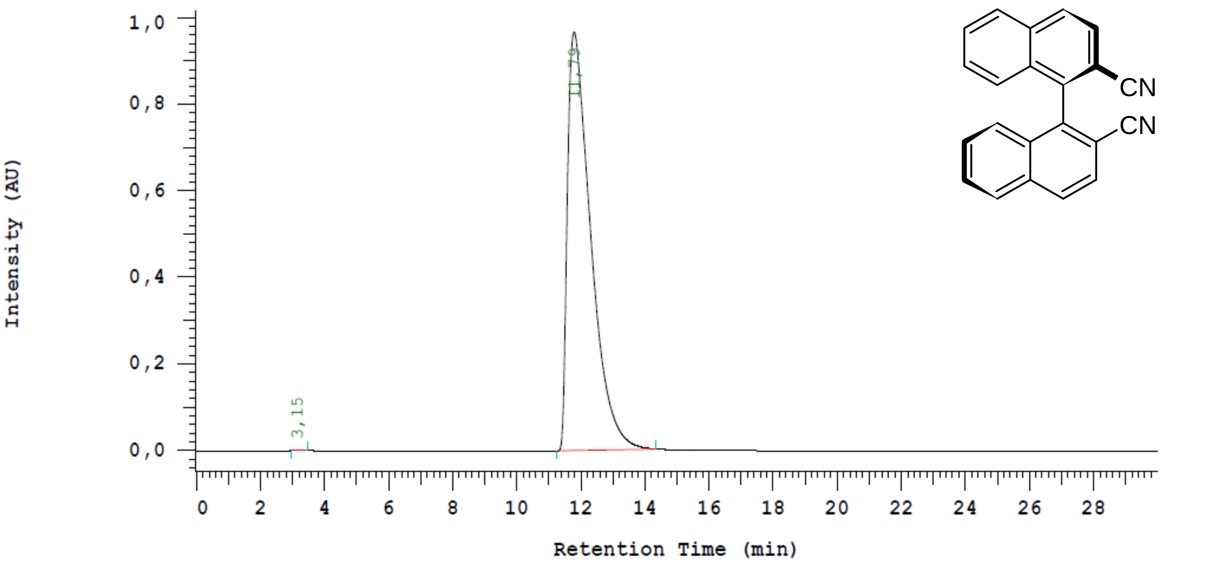

No.	RT	Area	Area %	BC	R-Factor
1	3,15	14319	0,059	BB	
2	11,79	24270552	99,941	BB	
		24284871	100,000		

Compound 14(*S*):
Column: Nucleodur® PolarTec (5 µm, 4.6×250 mm)
Eluent: 25 mM Phosphate Buffer pH 3/MeCN 30:70
Flowrate: 0.7 mL/min

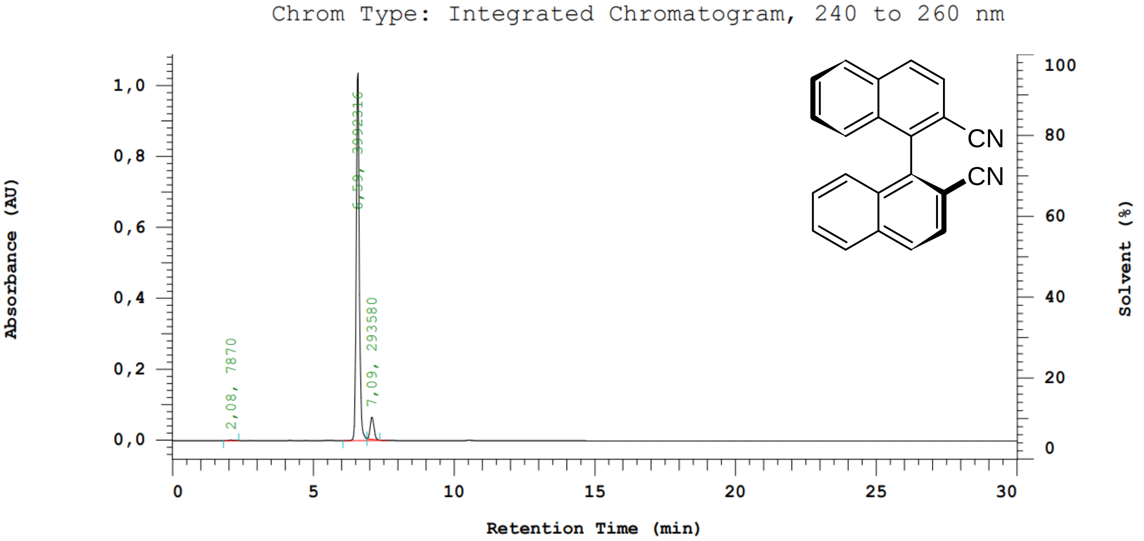

No.	RT	Area	Area %	BC	R-Factor	Purity	Height
1	2,08	7870	0,183	BB		0,1521	974
2	6,59	3992316	92,979	BV		0,9979	517943
3	7,09	293580	6,837	TBB		0,9995	31500
		4293766	100,000				550417

Column: Chiralcel® OD-H (5 µm, 4.6×250 mm)
Eluent: *i*-PrOH/Hexane 10:90
Flowrate: 1 mL/min
Wavelength: 280 nm

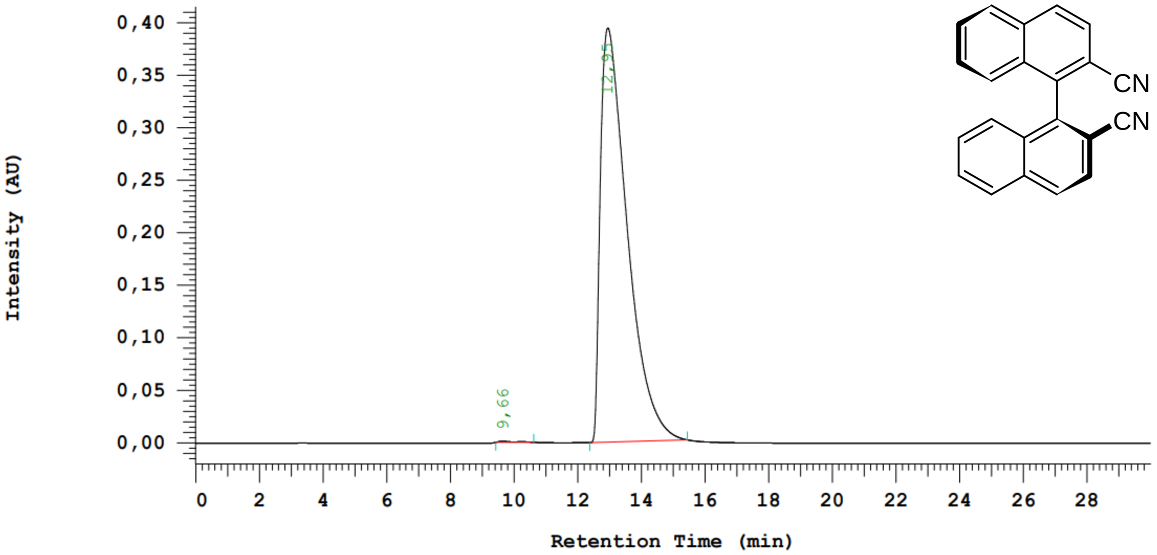

No.	RT	Area	Area %	BC	R-Factor
1	9,66	18300	0,158	BB	
2	12,95	11549182	99,842	BB	
		11567482	100,000		

Manually prepared mixture of compound 14(*R*) and 14(*S*):
Column: Chiralcel® OD-H (5 µm, 4.6×250 mm)
Eluent: *i*-PrOH/Hexane 10:90
Flowrate: 1 mL/min
Wavelength: 280 nm

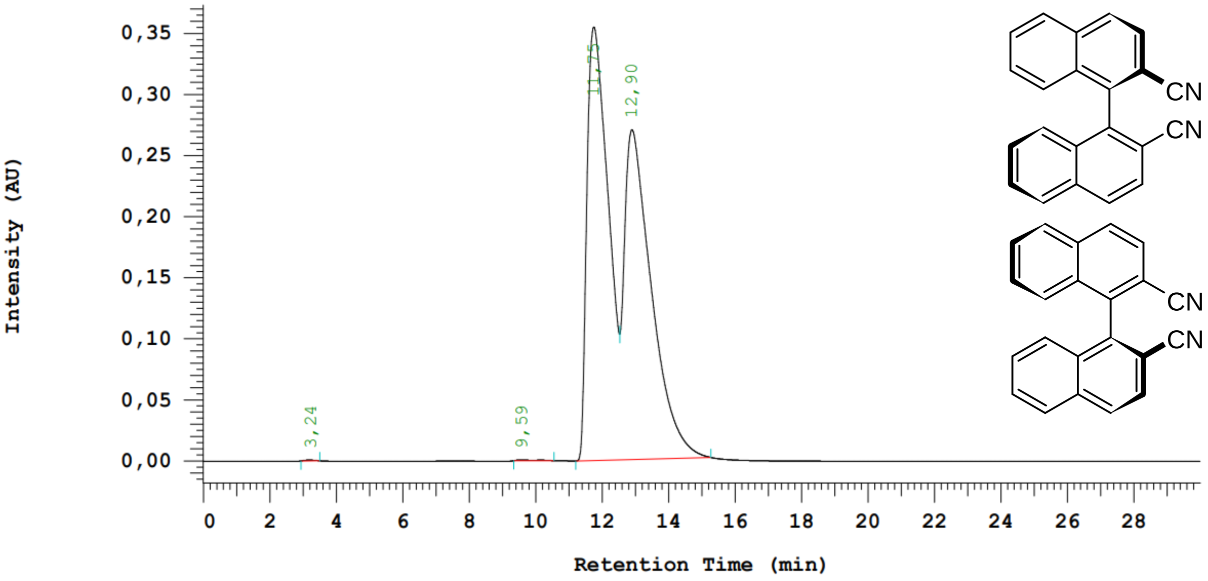

No.	RT	Area	Area %	BC	R-Factor
1	3,24	6534	0,042	BB	
2	9,59	12303	0,079	BB	
3	11,75	7846973	50,195	BV	
4	12,90	7767287	49,685	VB	
		15633097	100,000		

Compound 15(*R*):

Column: Nucleodur® C18 HTec (5 µm, 4.6×250 mm)
Eluent: 25 mM Acetate Buffer pH 5/MeCN 60:40
Flowrate: 0.7 mL/min

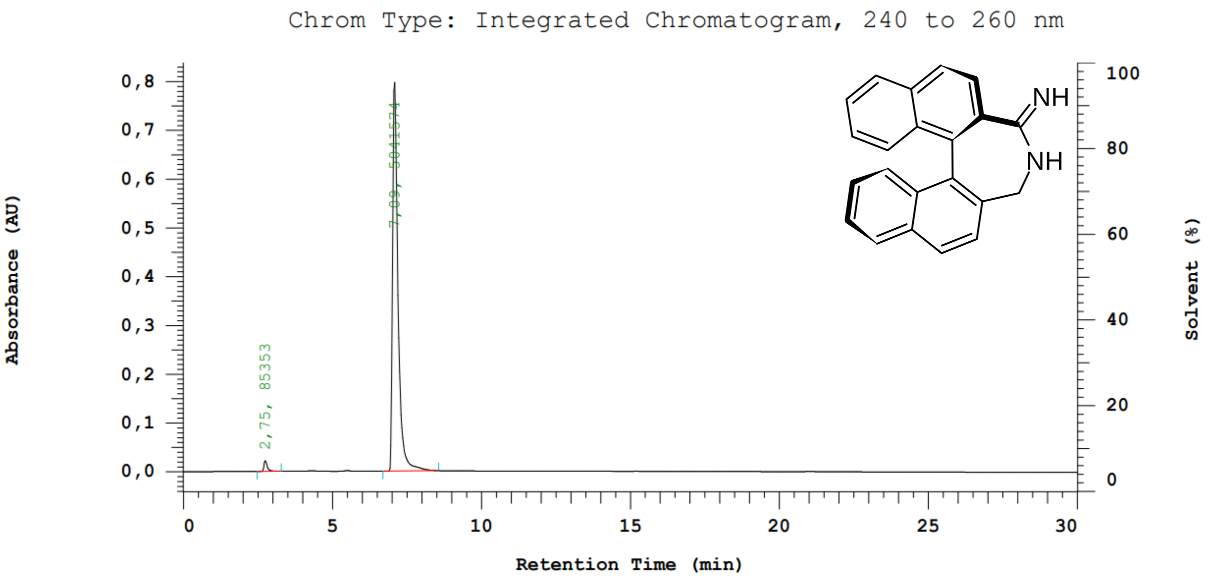

No.	RT	Area	Area %	BC	R-Factor	Purity	Height
1	2,75	85353	1,665	BB		0,9990	11039
2	7,09	5041574	98,335	BB		0,9992	398175
		5126927	100,000				409214

On the column Chiralcel® OD-H (5 µm, 4.6×250 mm) compound **15(*R*)** caused extreme band broadening.

Compound 15(*S*):

Column: Nucleodur® C18 HTec (5 µm, 4.6×250 mm)
Eluent: 25 mM Acetate Buffer pH 5/MeCN 70:30
Flowrate: 0.7 mL/min

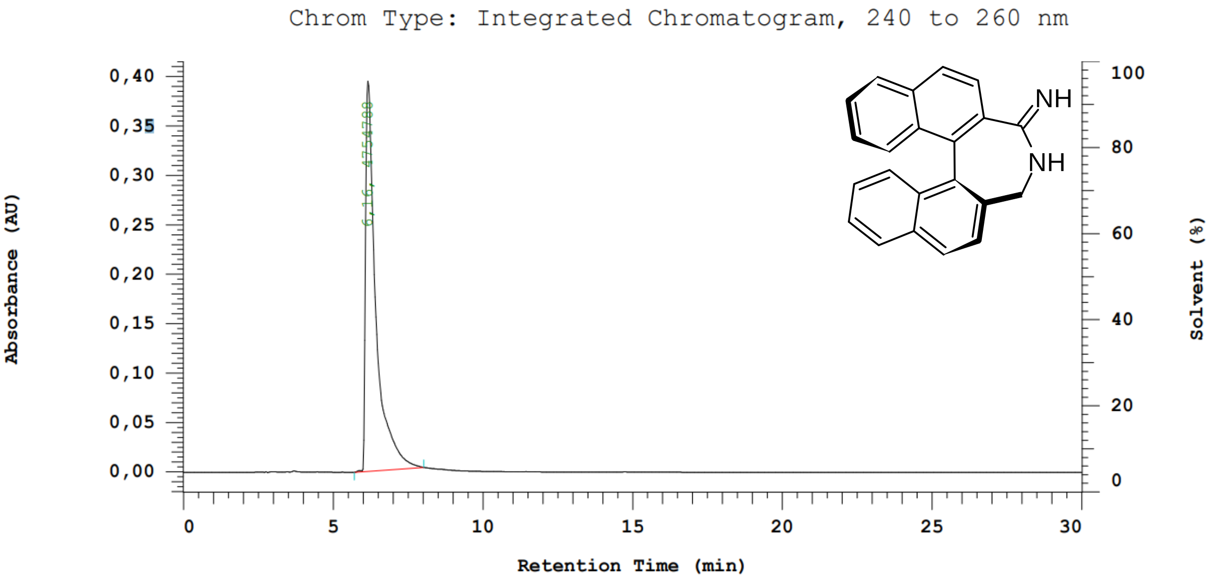

No.	RT	Area	Area %	BC	R-Factor	Purity	Height
1	6,16	4754788	100,000	BB		0,9997	197308
		4754788	100,000				197308

On the column Chiralcel® OD-H (5 µm, 4.6×250 mm) compound **15(*S*)** caused extreme band broadening.

Compound 16(*R*):

Column: Nucleodur® C18 HTec (5 µm, 4.6×250 mm)
Eluent:
0 → 1 min: 25 mM Acetate Buffer pH 5/MeCN 90:10
1 → 10 min: gradient to 25 mM Acetate Buffer pH 5/MeCN 10:90
10 → 20 min: 25 mM Acetate Buffer pH 5/MeCN 10:90
20 → 25 min: gradient to 25 mM Acetate Buffer pH 5/MeCN 90:10
25 → 30 min: 25 mM Acetate Buffer pH 5/MeCN 90:10
Flowrate: 0.7 mL/min

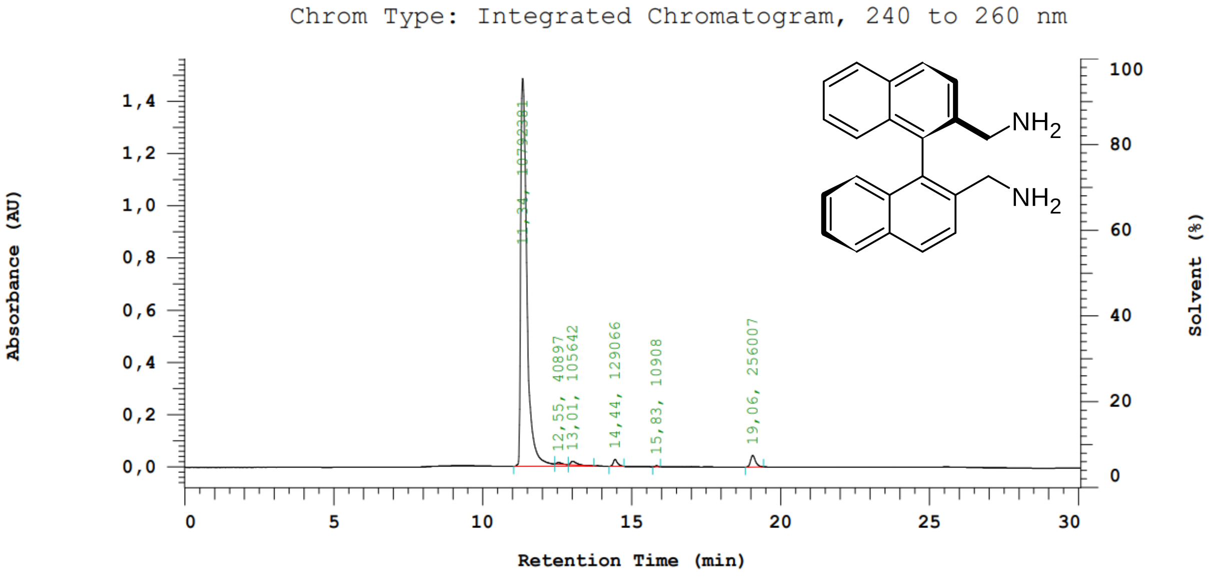

No.	RT	Area	Area %	BC	R-Factor	Purity	Height
1	11,34	10792381	95,214	BV		0,9995	742043
2	12,55	40897	0,361	TBB		0,9905	3312
3	13,01	105642	0,932	TBB		0,9823	6960
4	14,44	129066	1,139	BB		0,9860	13206
5	15,83	10908	0,096	BB		1,0000	1792
6	19,06	256007	2,259	BB		1,0000	22427
		11334901	100,000				789740

On the column Chiralcel® OD-H (5 µm, 4.6×250 mm) compound **16(*R*)** caused extreme band broadening.

Compound 16(*S*):

Column: Nucleodur® C18 HTec (5 µm, 4.6×250 mm)
Eluent:
0 → 1 min: 25 mM Acetate Buffer pH 5/MeCN 90:10
1 → 10 min: gradient to 25 mM Acetate Buffer pH 5/MeCN 10:90
10 → 20 min: 25 mM Acetate Buffer pH 5/MeCN 10:90
20 → 25 min: gradient to 25 mM Acetate Buffer pH 5/MeCN 90:10
25 → 30 min: 25 mM Acetate Buffer pH 5/MeCN 90:10
Flowrate: 0.7 mL/min

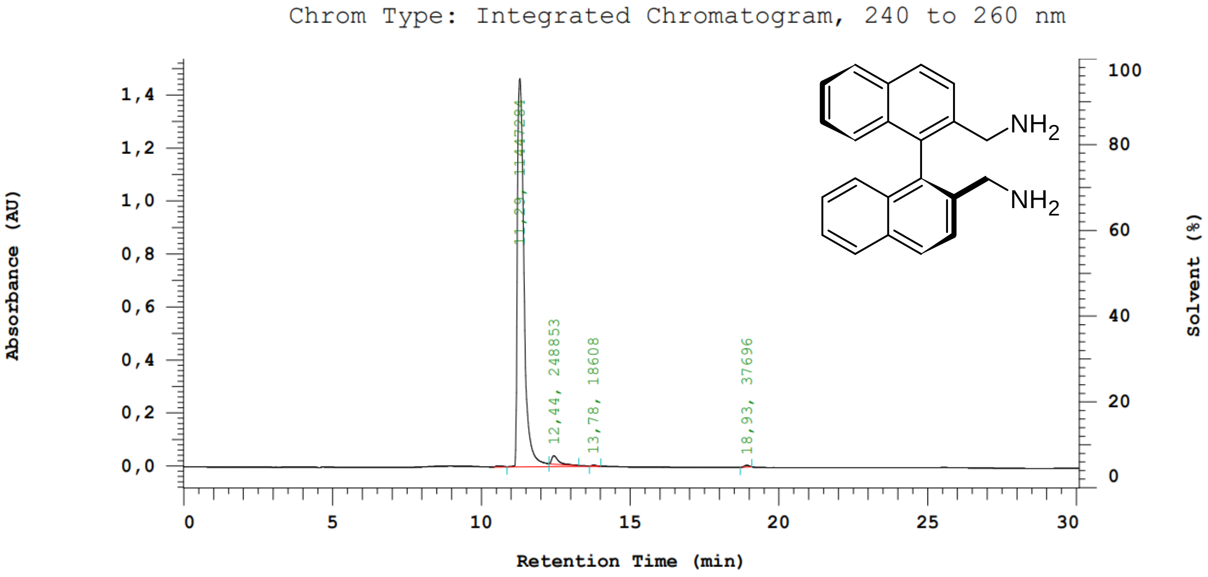

No.	RT	Area	Area %	BC	R-Factor	Purity	Height
1	11,29	11447284	97,403	BV		0,9998	732401
2	12,44	248853	2,117	TBB		0,9734	15707
3	13,78	18608	0,158	TBB		0,9916	1849
4	18,93	37696	0,321	BB		0,9999	3309
		11752441	100,000				753266

On the column Chiralcel® OD-H (5 µm, 4.6×250 mm) compound **16(*S*)** caused extreme band broadening.

Compound 17:
Column: Nucleodur® PolarTec (5 µm, 4.6×250 mm)
Eluent: 25 mM Phosphate Buffer pH 3/MeCN 70:30
Flowrate: 0.7 mL/min

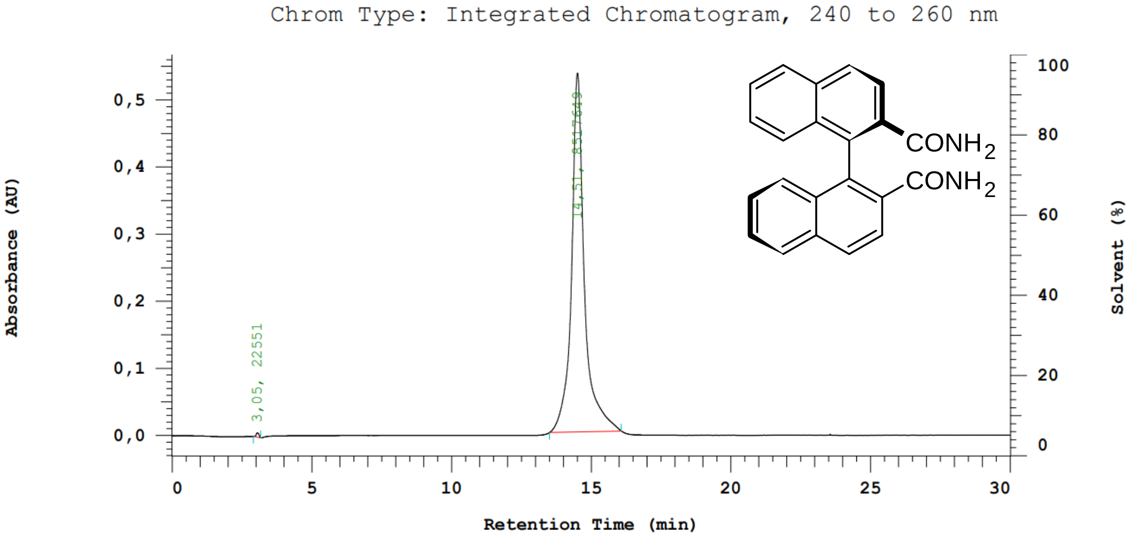

No.	RT	Area	Area %	BC	R-Factor	Purity	Height
1	3,05	22551	0,264	BB		0,9298	3219
2	14,51	8517649	99,736	BB		1,0000	267223
		8540200	100,000				270442

Column: Chiralcel® OD-H (5 µm, 4.6×250 mm)
Eluent: *i*-PrOH/Hexane 20:80
Flowrate: 1 mL/min
Wavelength: 280 nm

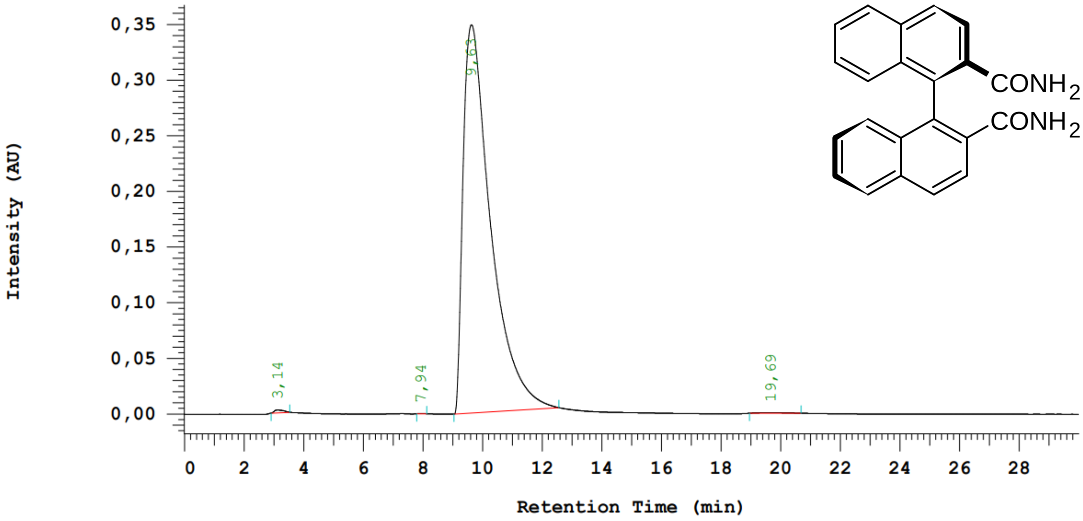

No.	RT	Area	Area %	BC	R-Factor
1	3,14	27201	0,242	BB	
2	7,94	1425	0,013	BB	
3	9,63	11174251	99,589	BB	
4	19,69	17444	0,155	BB	
		11220321	100,000		

CD spectra

Compound 2:

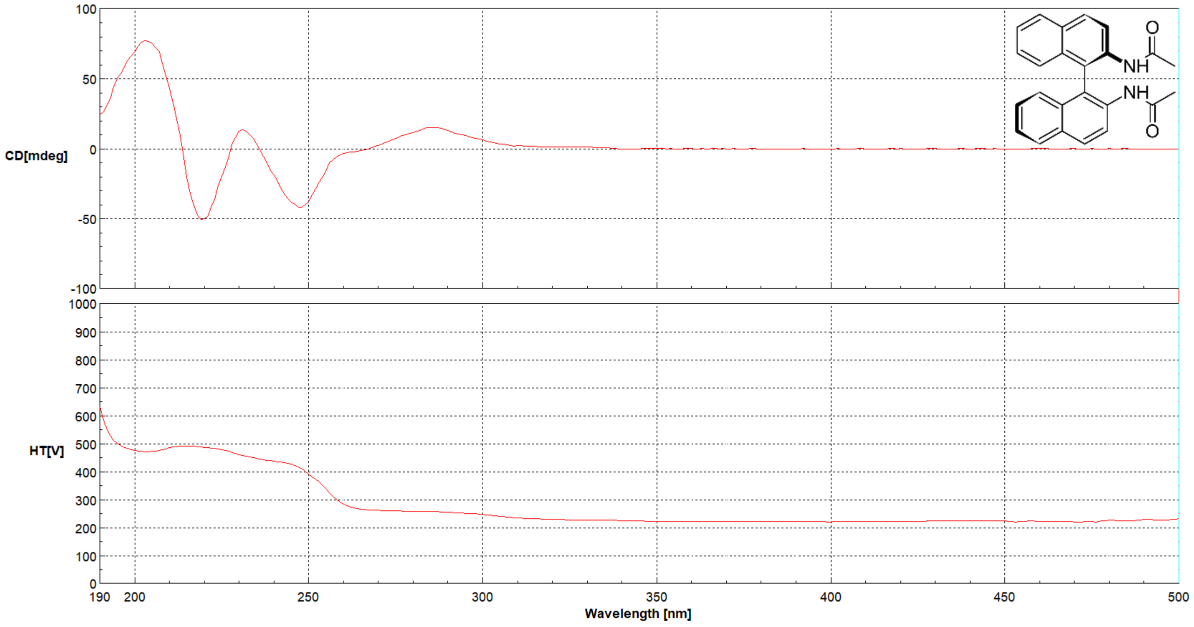

Compound 3:

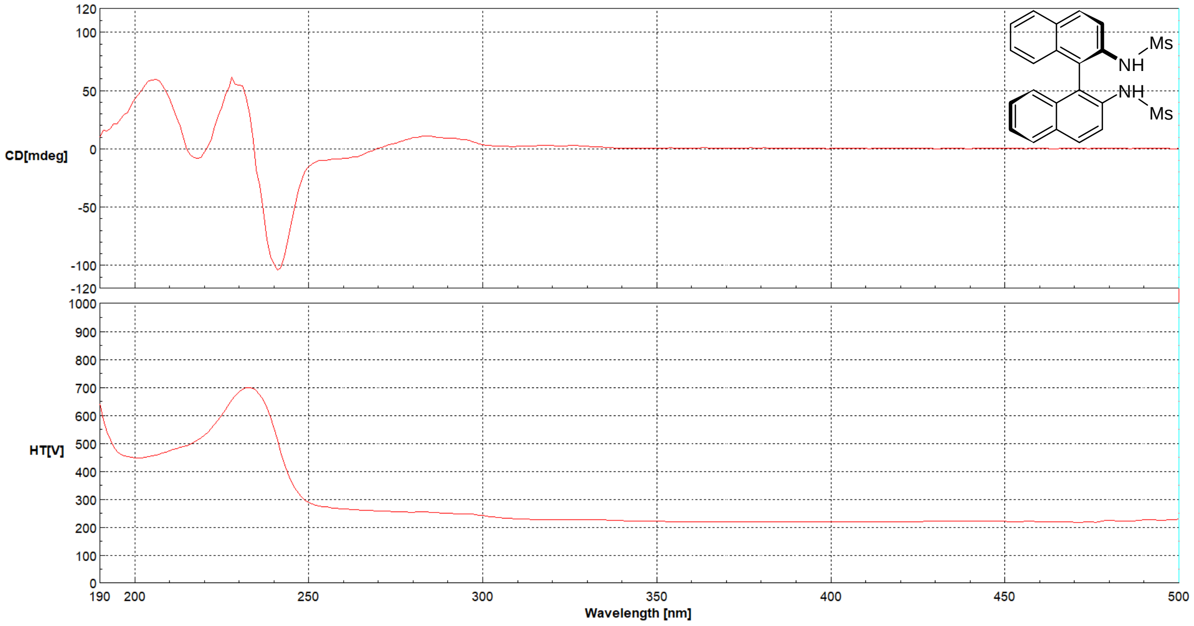

Compound 4:

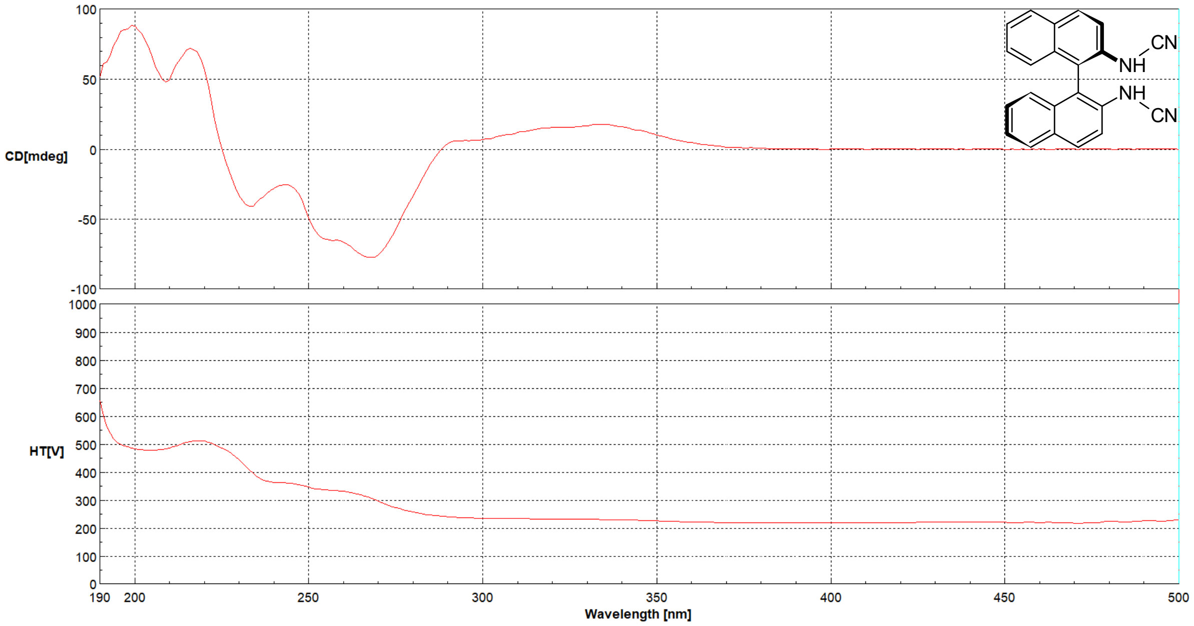

Compound 6:

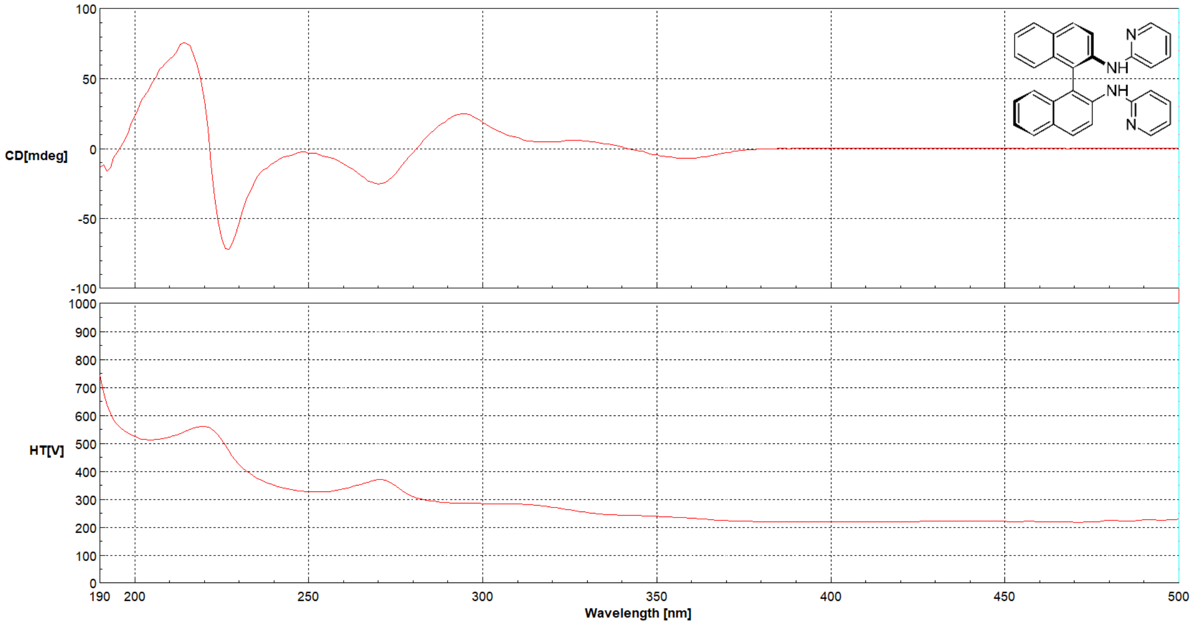

Compound 7:

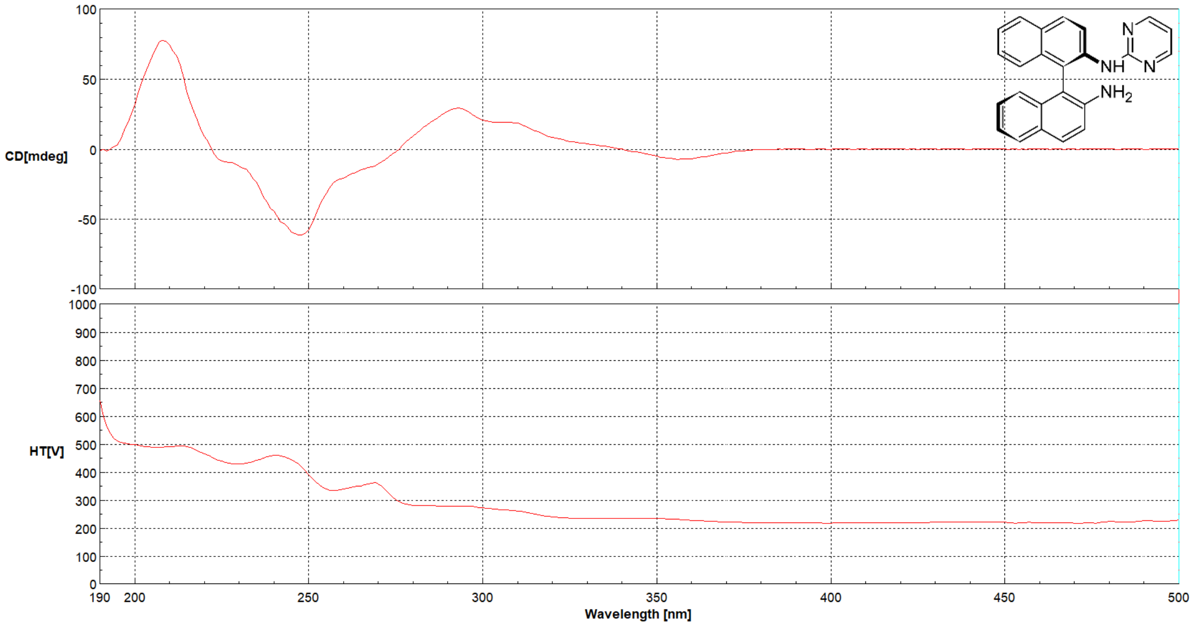

Compound 8:

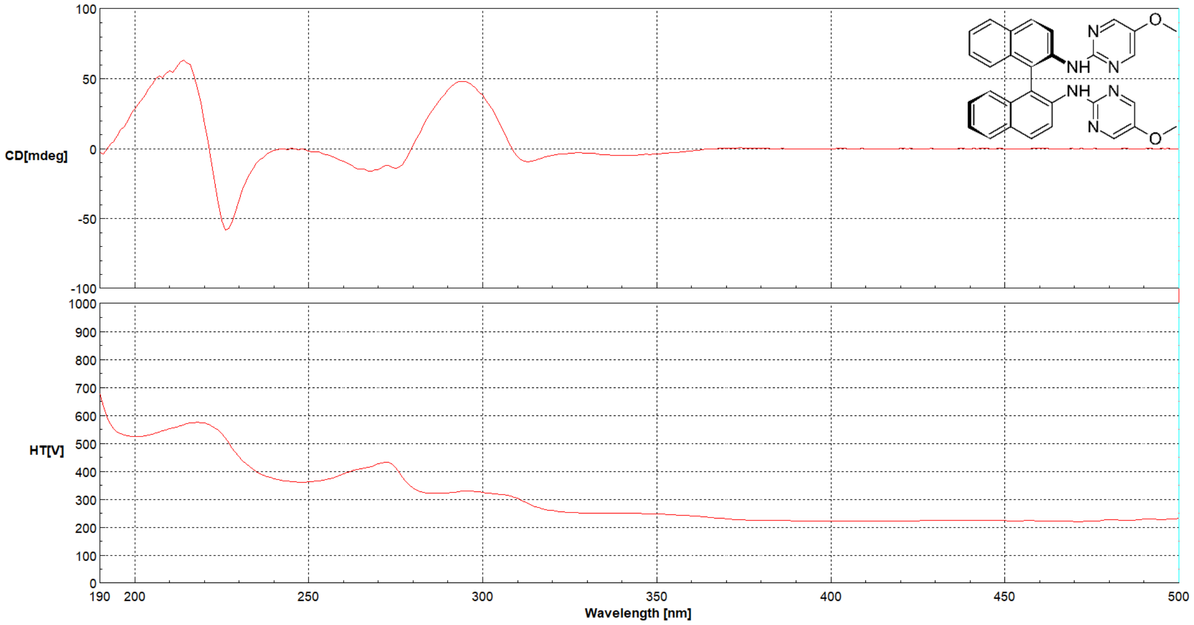

Compound 9:

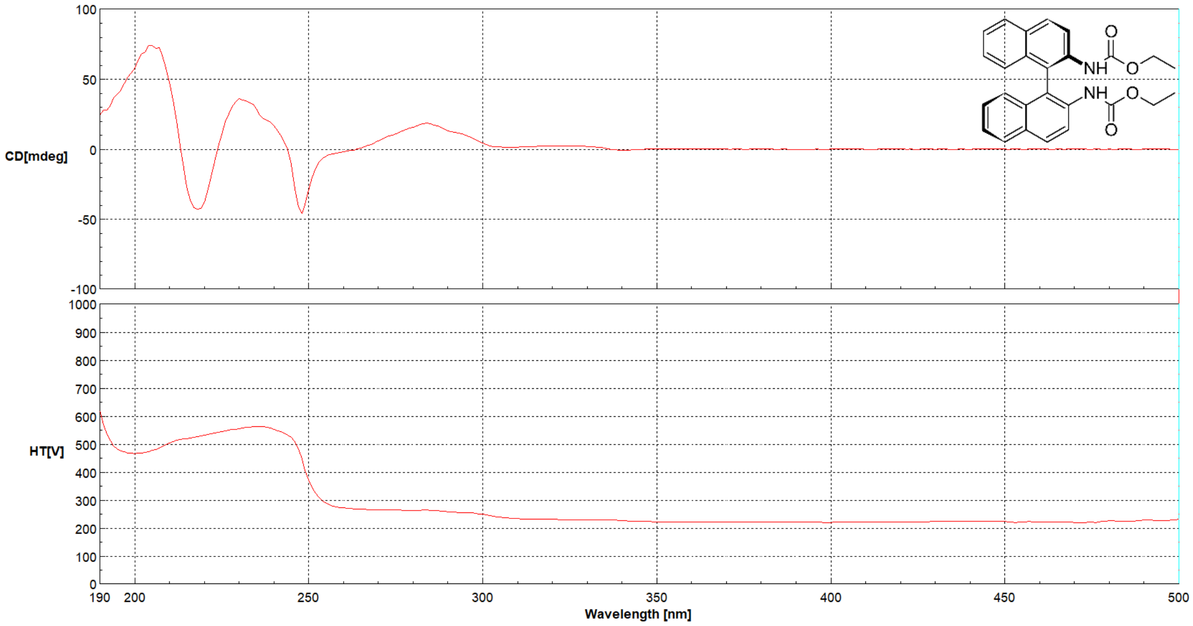

Compound 10:

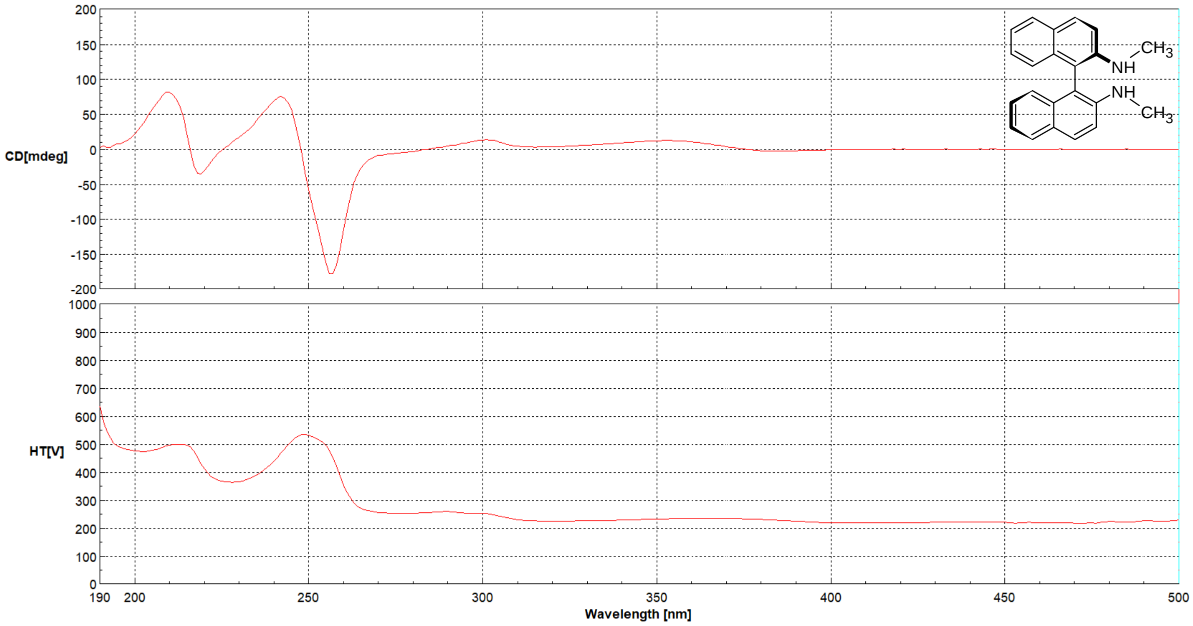

Compound 11(*R*) and 11(*S*):

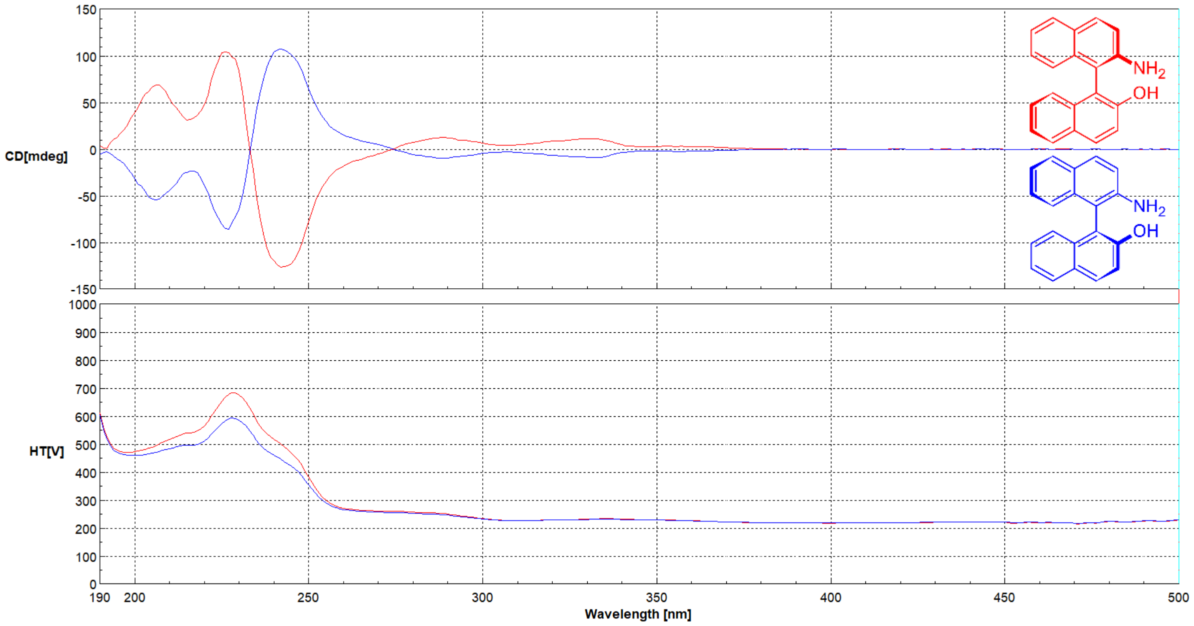

Compound 12(*R*) and 12(*S*):

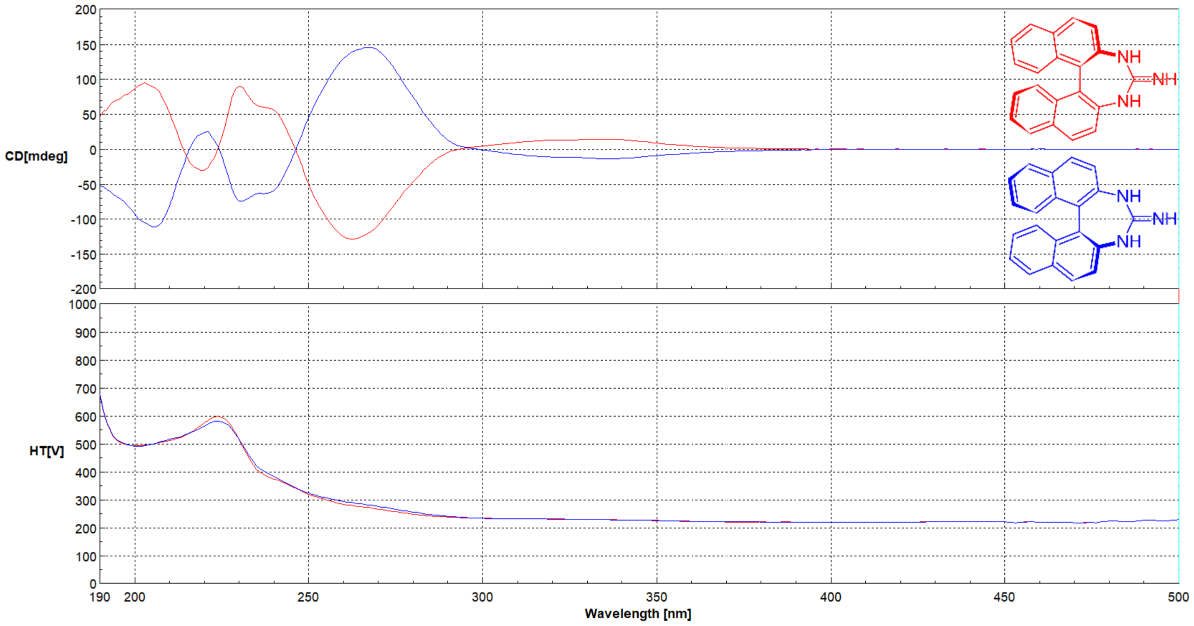

Compound 14(*R*) and 14(*S*):

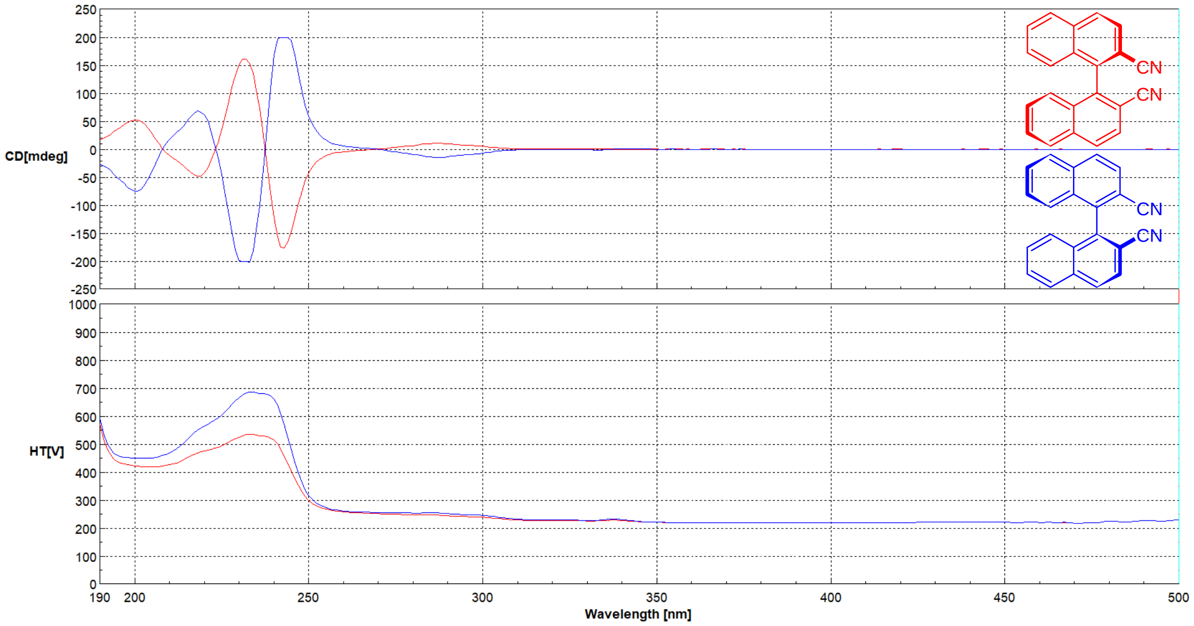

Compound 15(*R*) and 15(*S*):

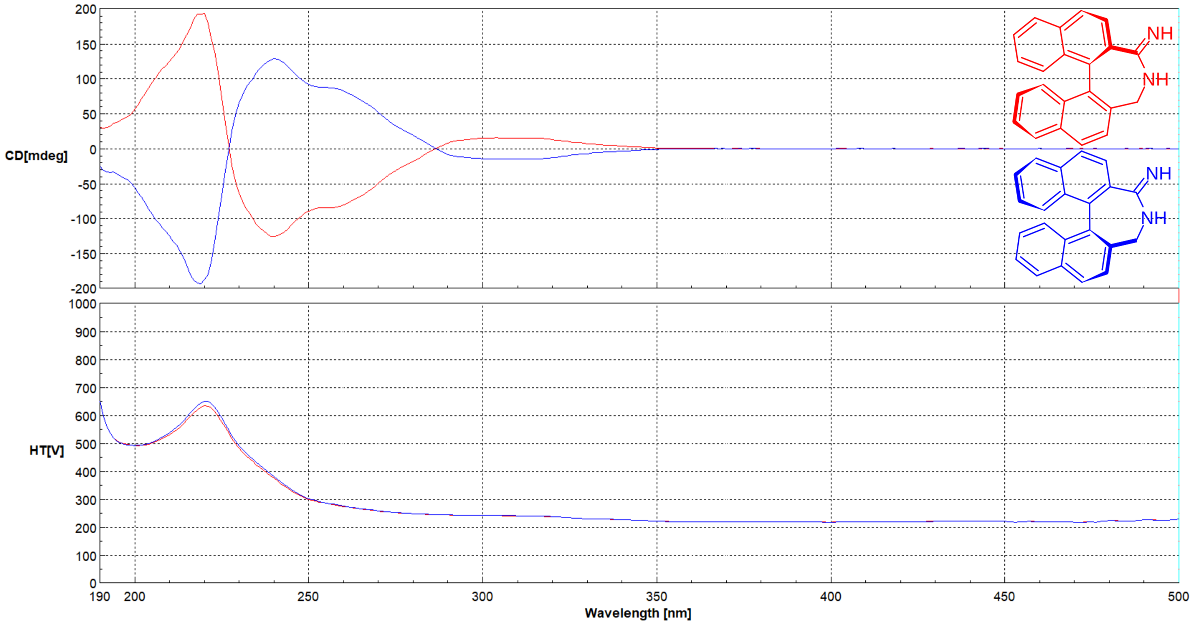

Compound 16(*R*) and 16(*S*):

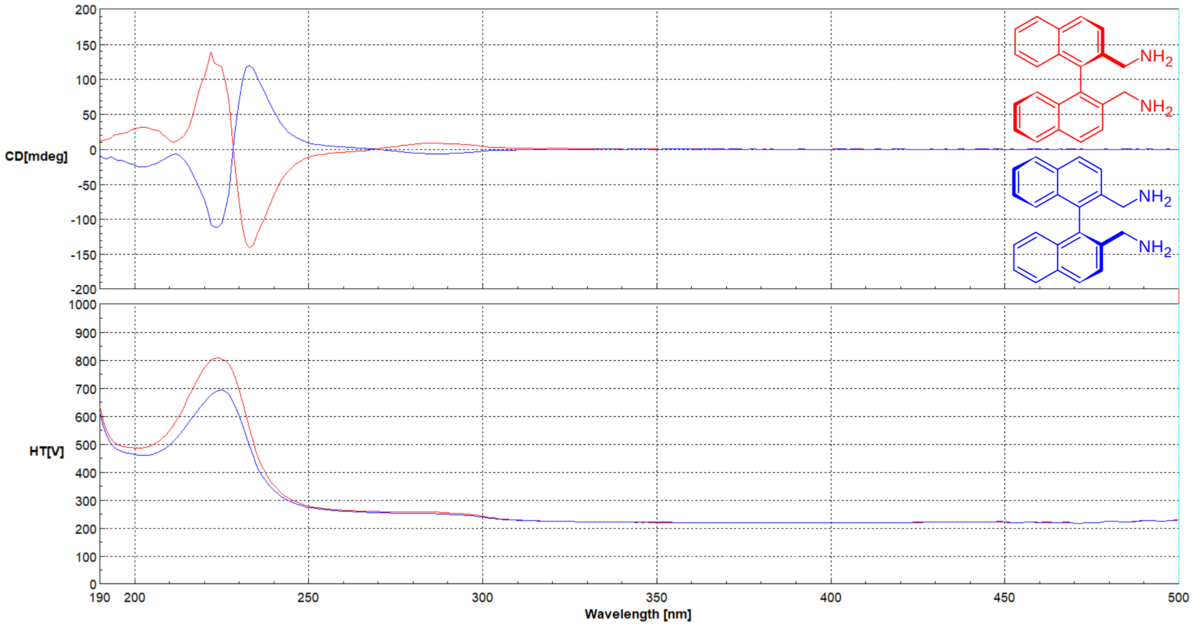

Compound 17:

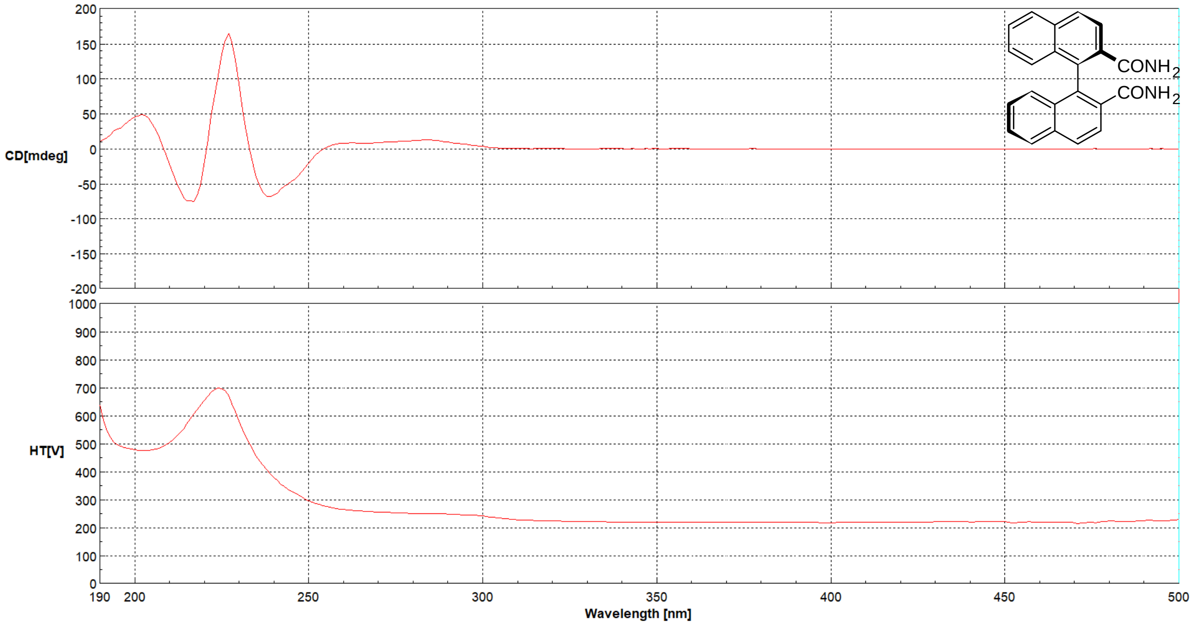

NMR spectra

Compound 2 - ^{1}H-NMR (DMSO-*d6*)

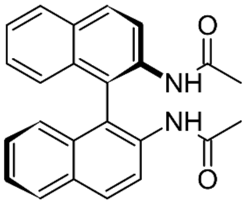

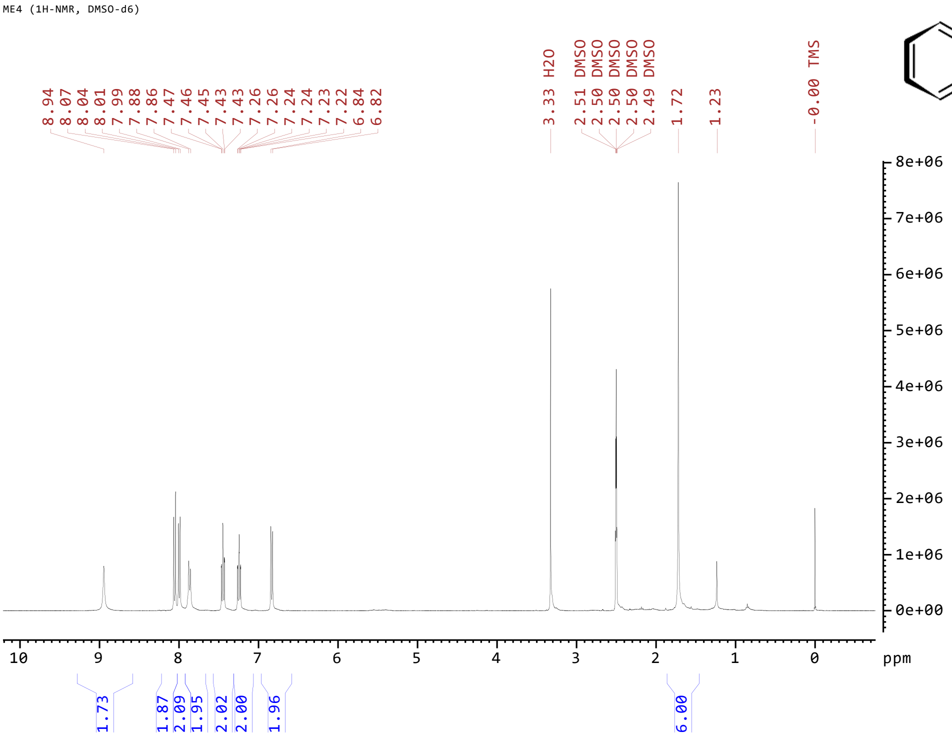

Compound 2 - ^{13}C-NMR (DMSO-*d6*)

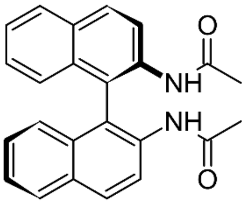

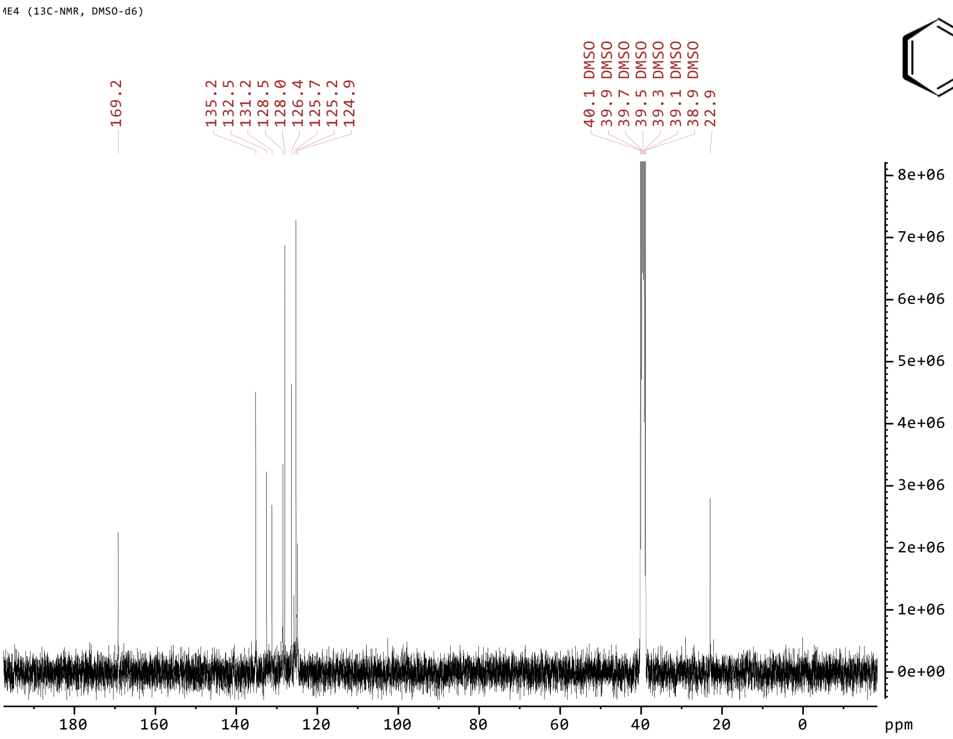

Compound 3 - ^{1}H-NMR (DMSO-*d6*)

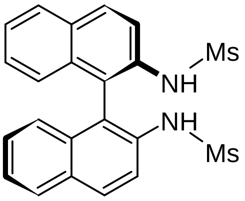

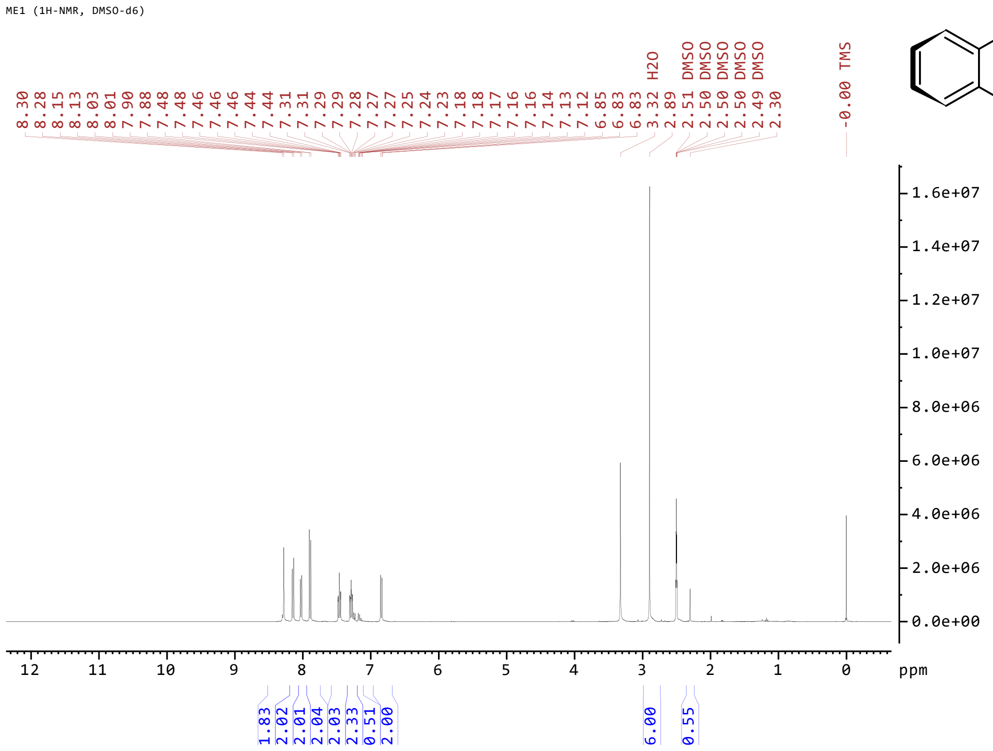

Compound 3 - ^{13}C-NMR (DMSO-*d6*)

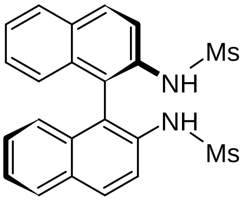

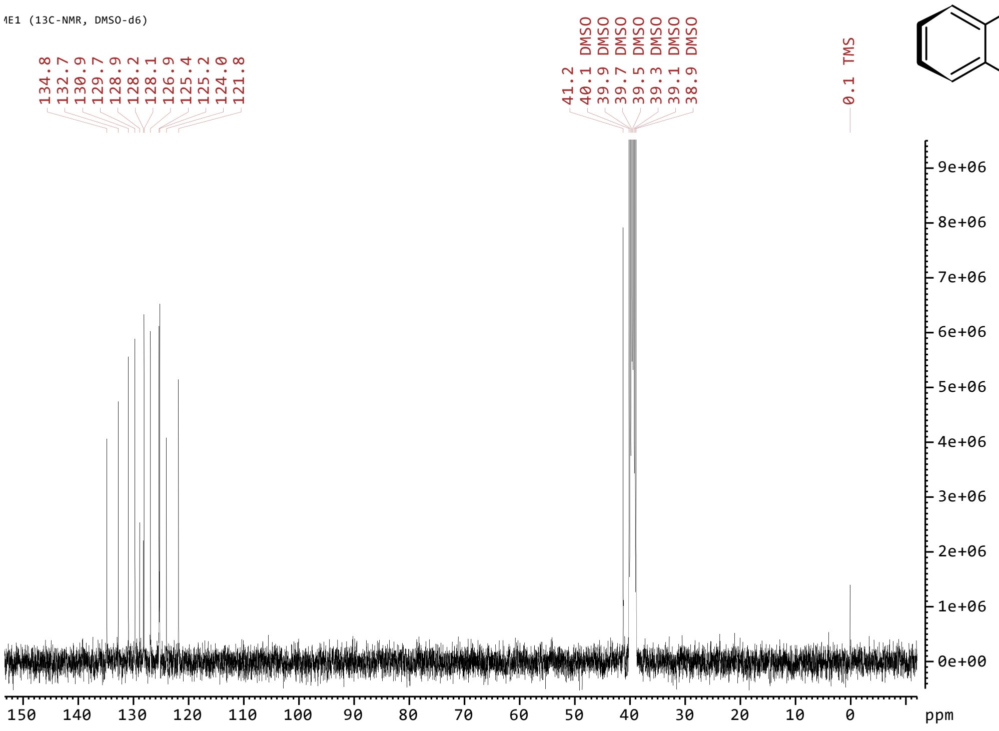

Compound 4 - ^{1}H-NMR (DMSO-*d6*)

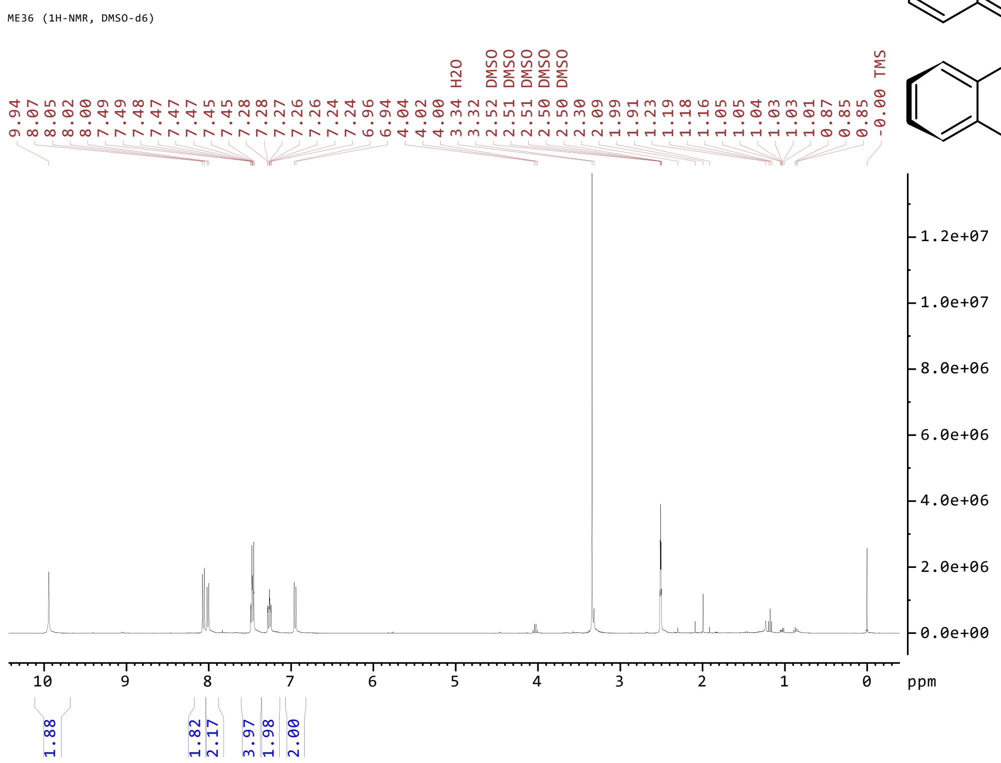

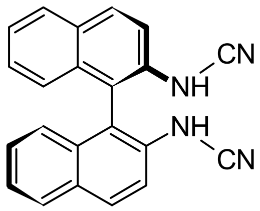

Compound 4 - ^{13}C-NMR (DMSO-*d6*)

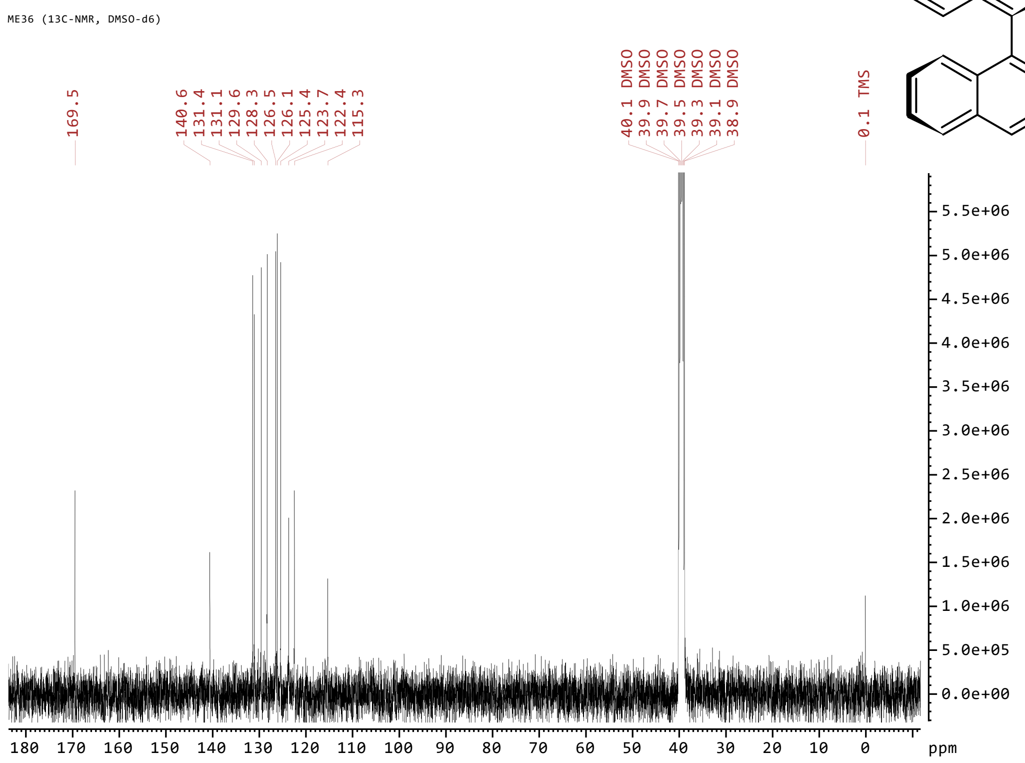

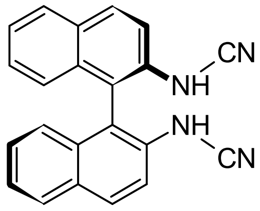

Compound 5 - ^{1}H-NMR (DMSO-*d6*)

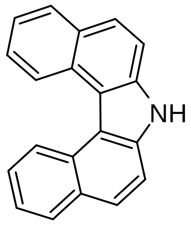

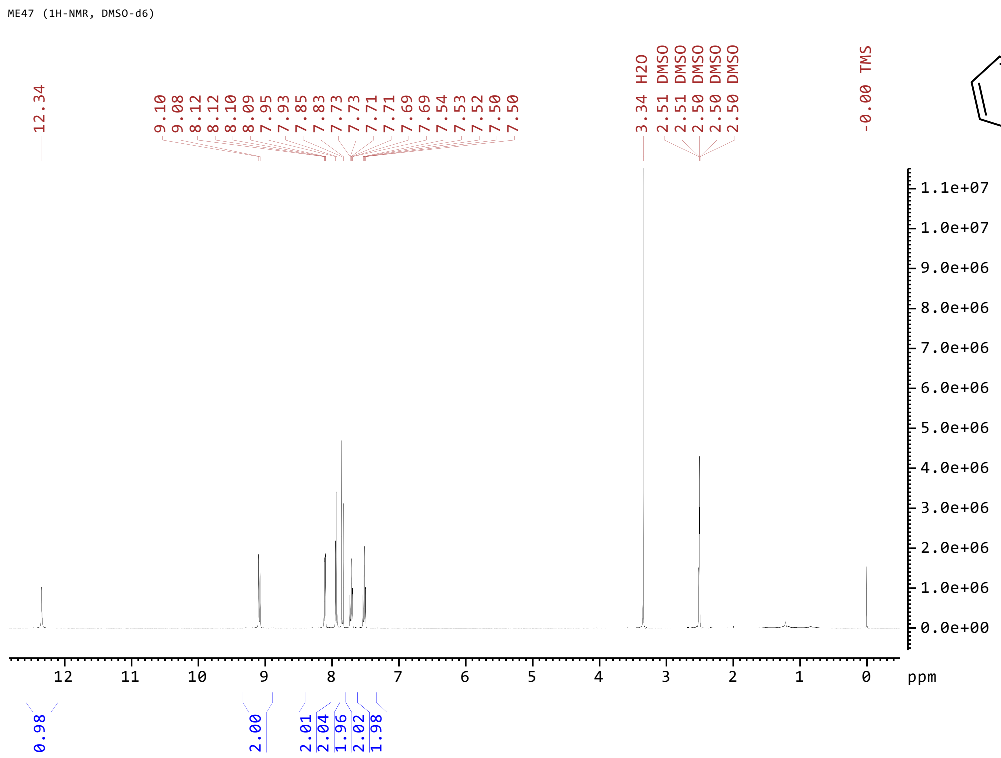

Compound 5 - ^{13}C-NMR (DMSO-*d6*)

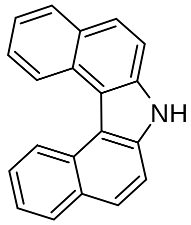

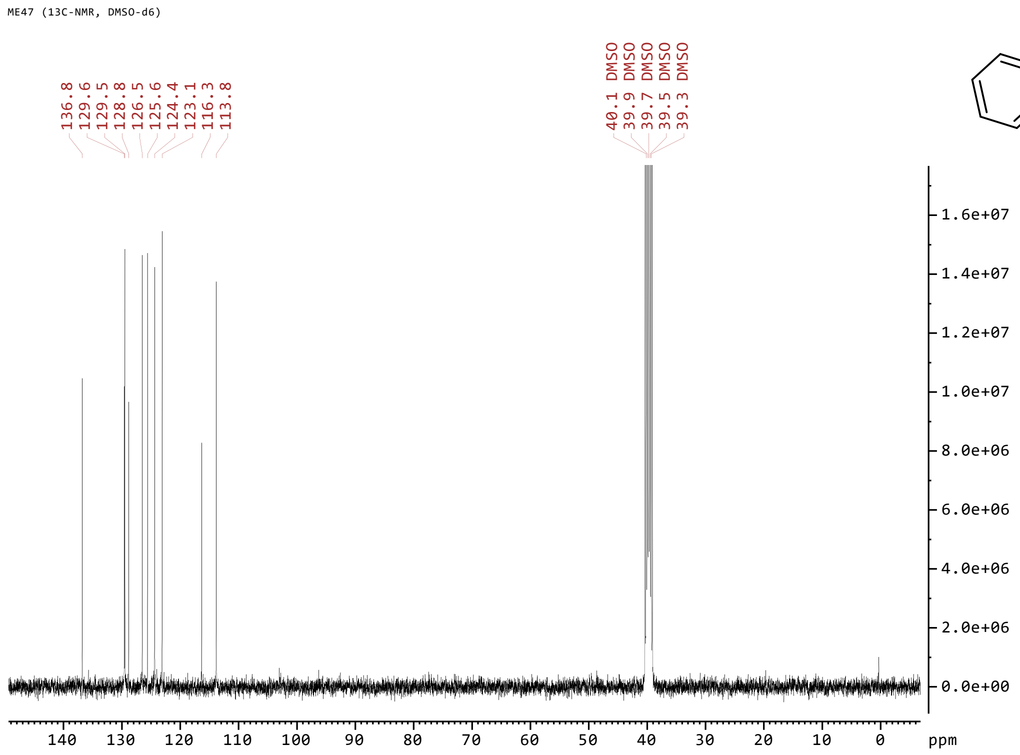

Compound 6 - ^{1}H-NMR (DMSO-*d*6)

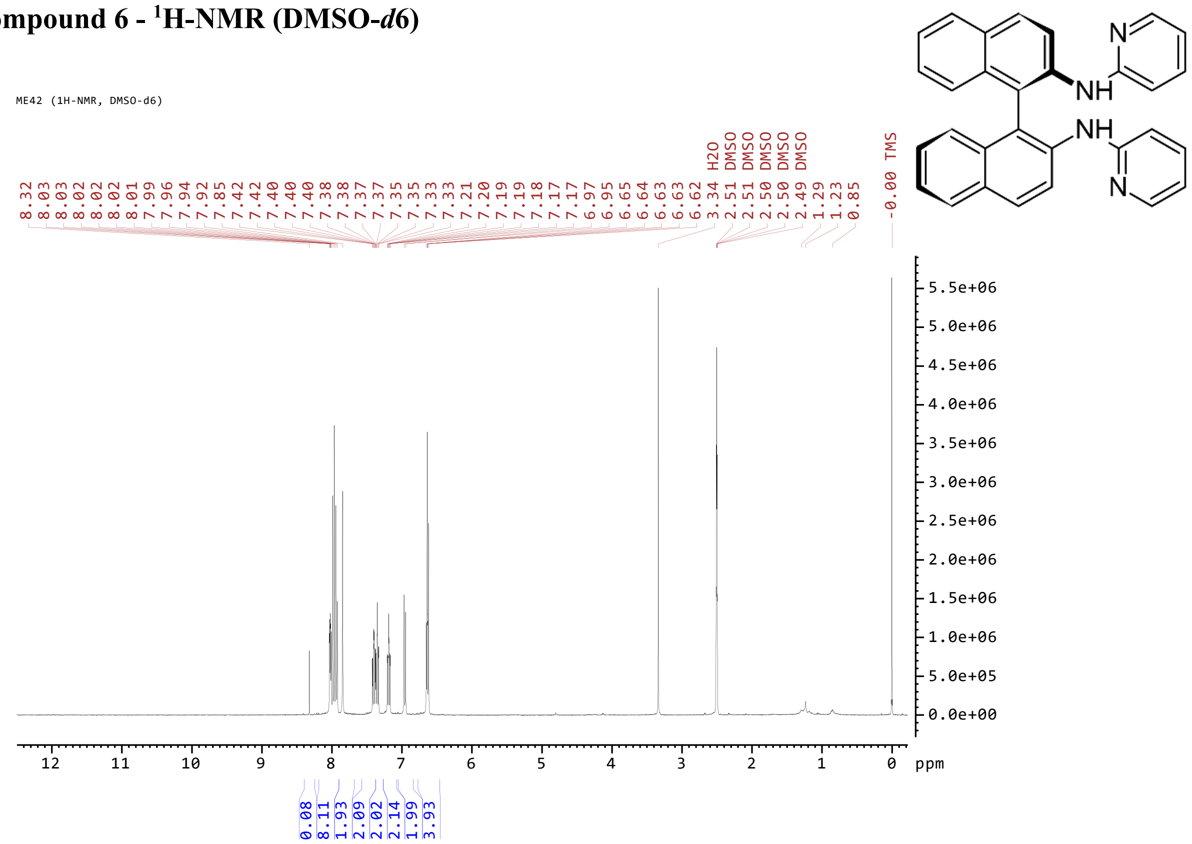

Compound 6 - ^{13}C-NMR (DMSO-*d*6)

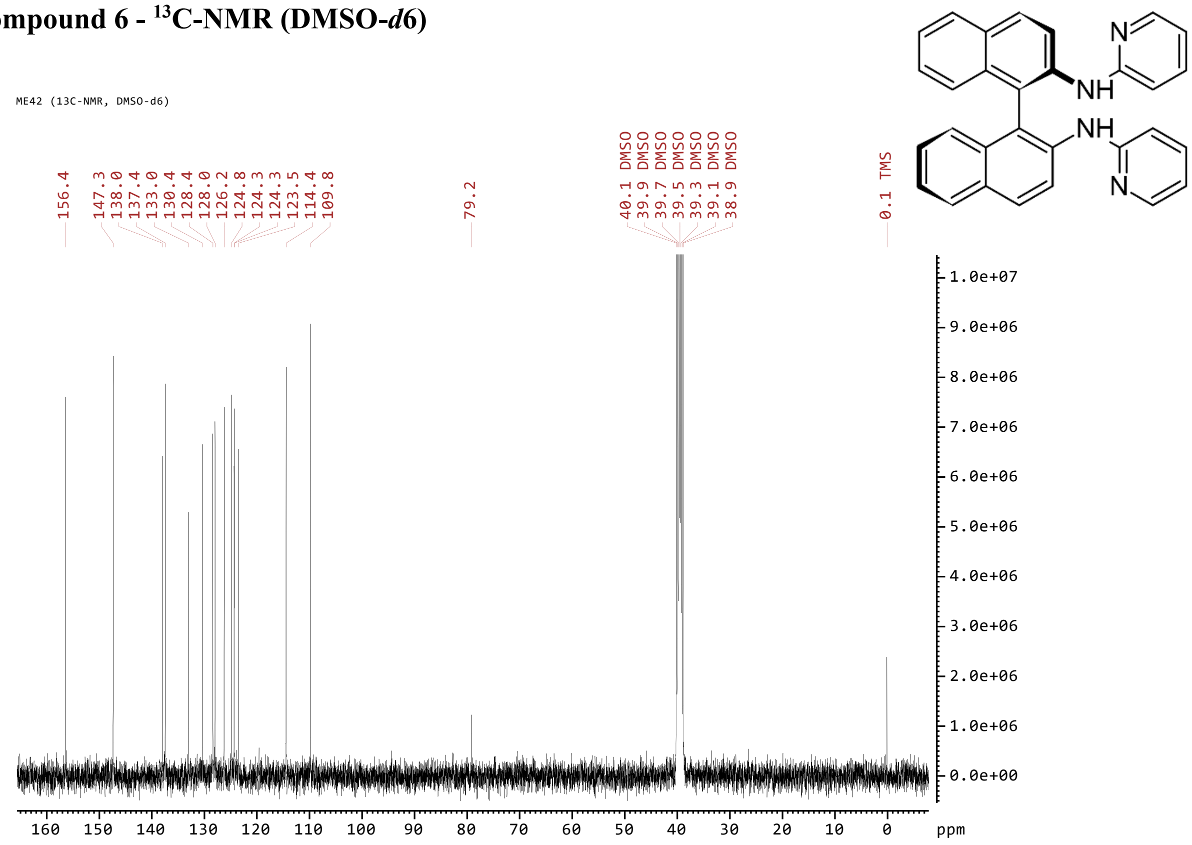

Compound 7 - ^{1}H-NMR (DMSO-*d*6)

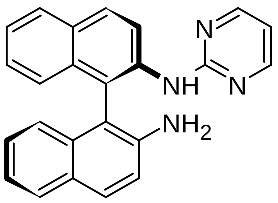

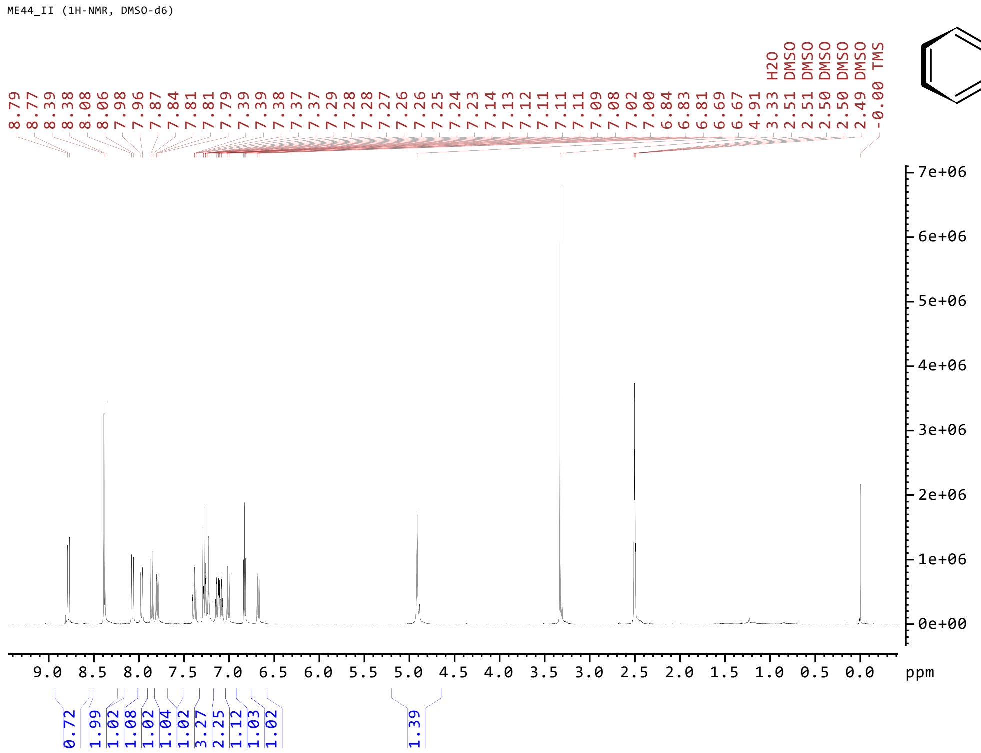

Compound 7 - ^{13}C-NMR (DMSO-*d*6)

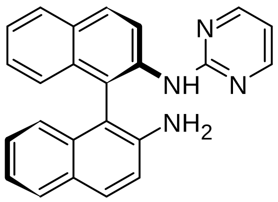

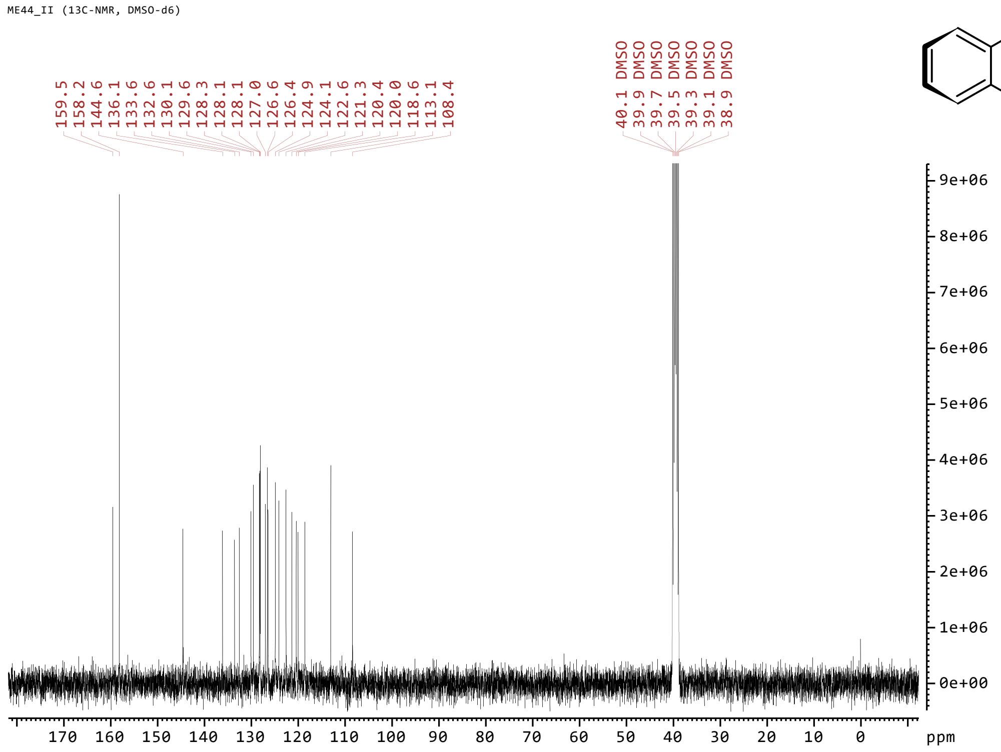

Compound 8 - ^{1}H-NMR (DMSO-*d*6)

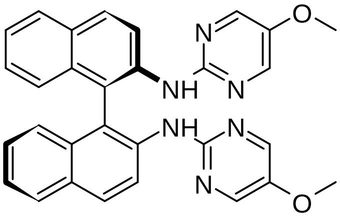

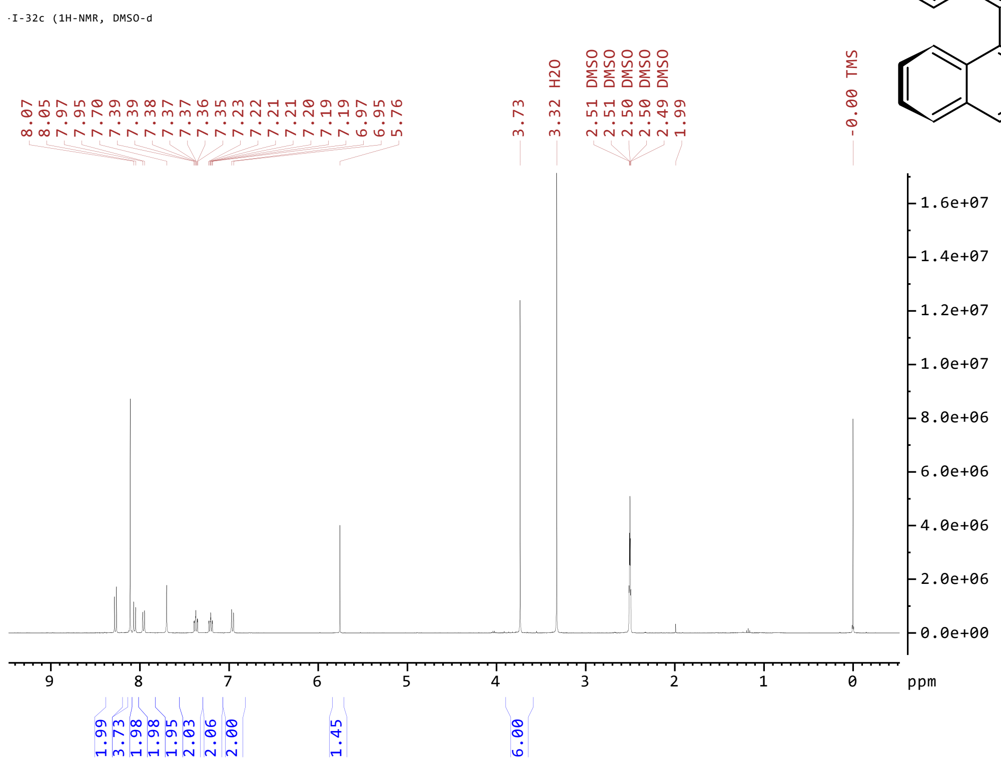

Compound 8 - ^{13}C-NMR (DMSO-*d*6)

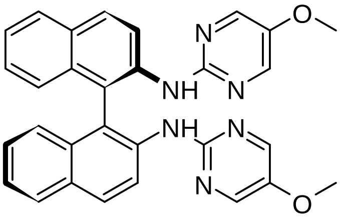

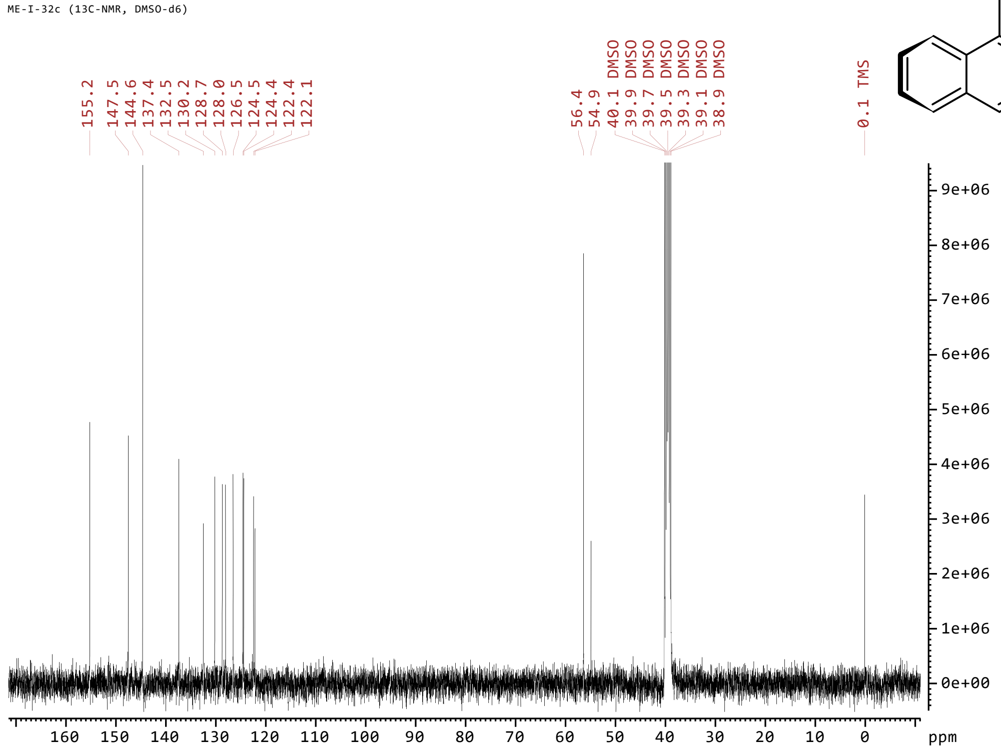

Compound 9 - ¹H-NMR (DMSO-*d6*)

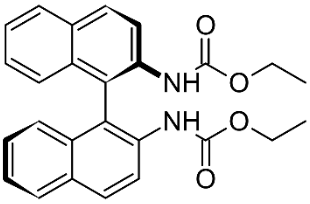

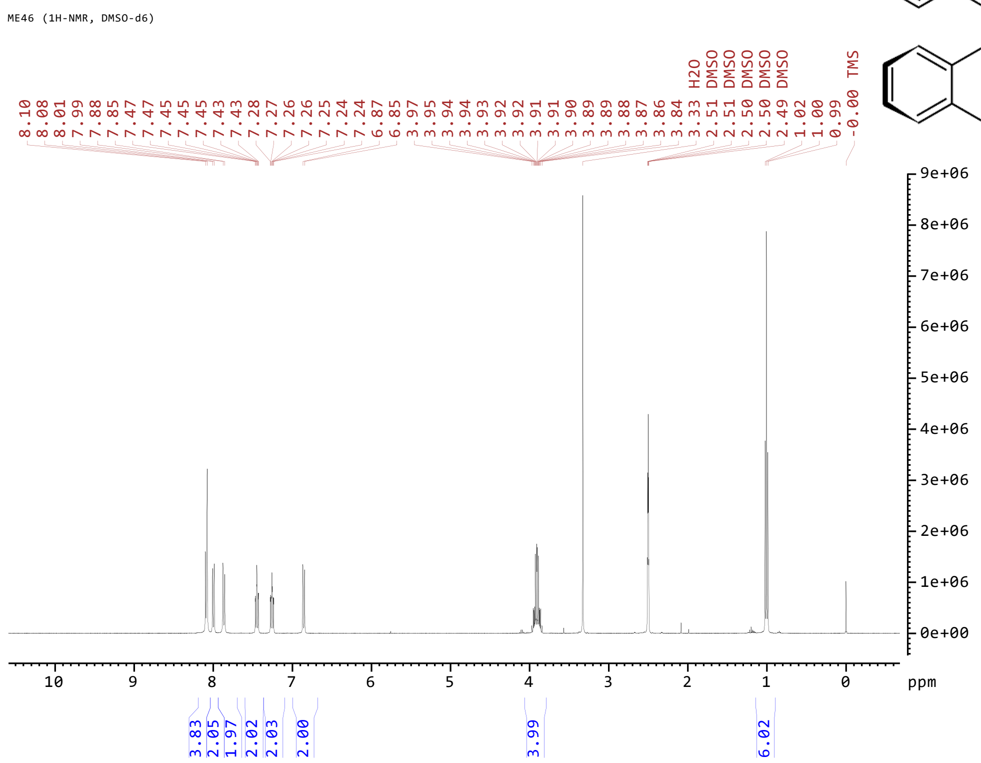

Compound 9 - ¹³C-NMR (DMSO-*d6*)

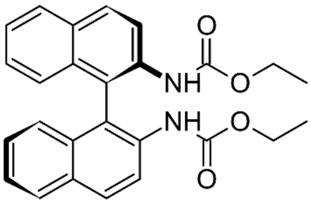

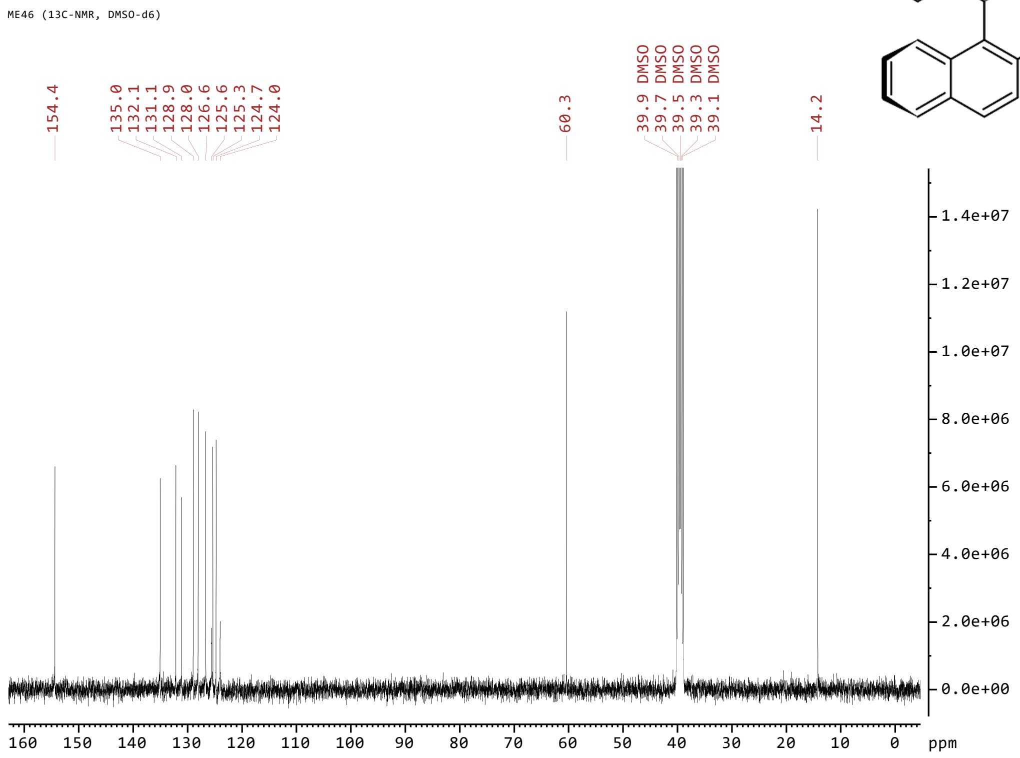

Compound 10 - ^{1}H-NMR (DMSO-*d6*)

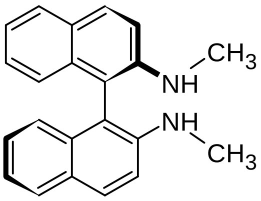

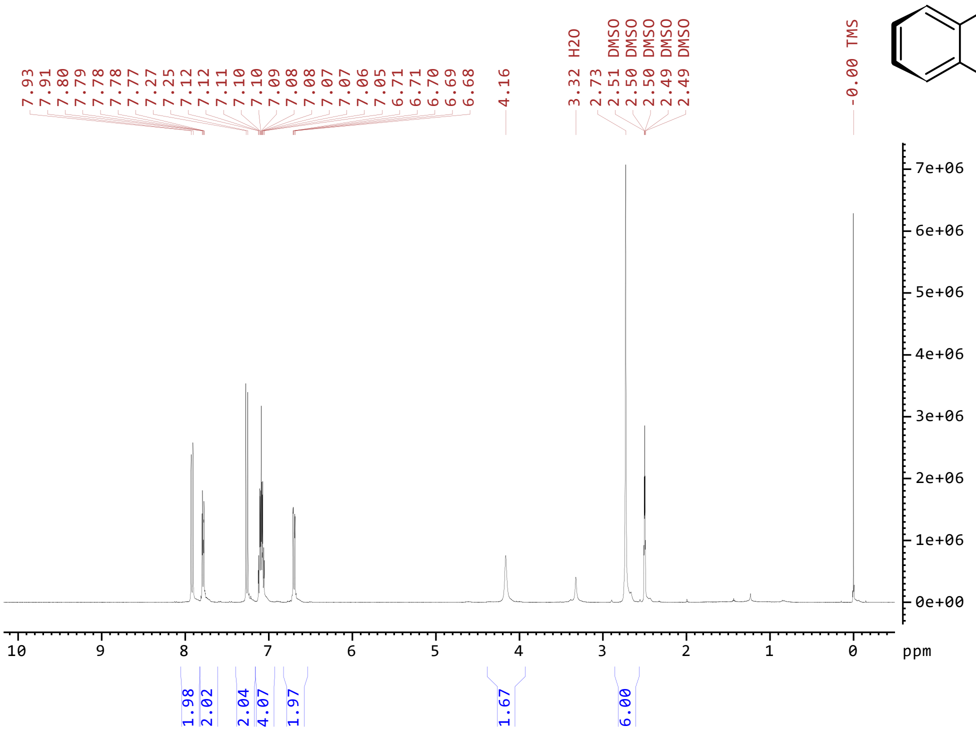

Compound 10 - ^{13}C-NMR (DMSO-*d6*)

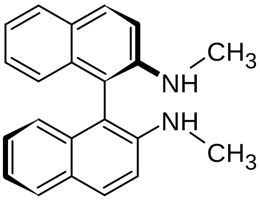

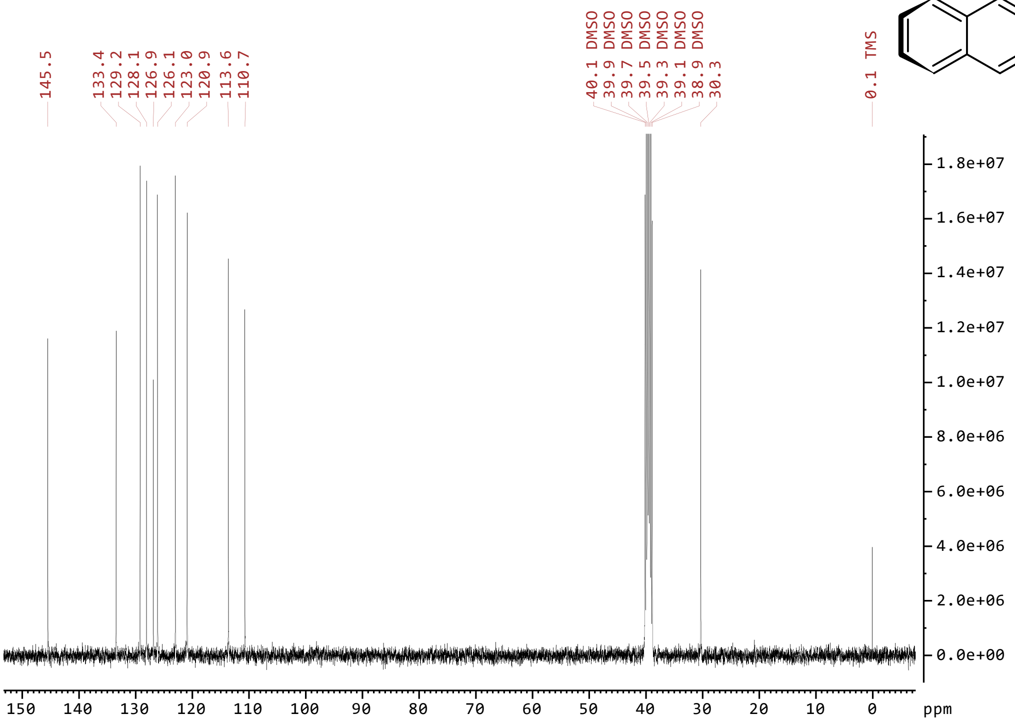

Compound 11(*R*) - ¹H-NMR (DMSO-*d*6)

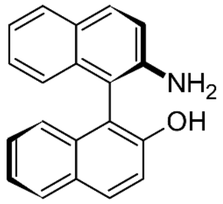

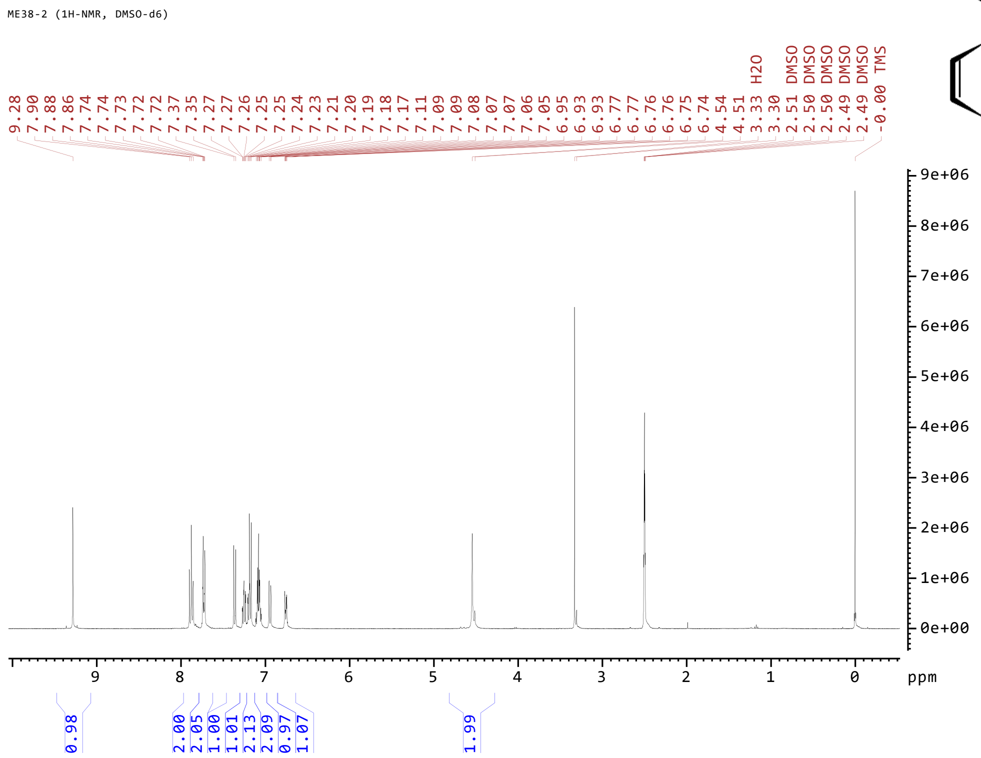

Compound 11(*R*) - ¹³C-NMR (DMSO-*d*6)

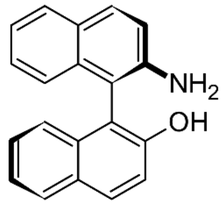

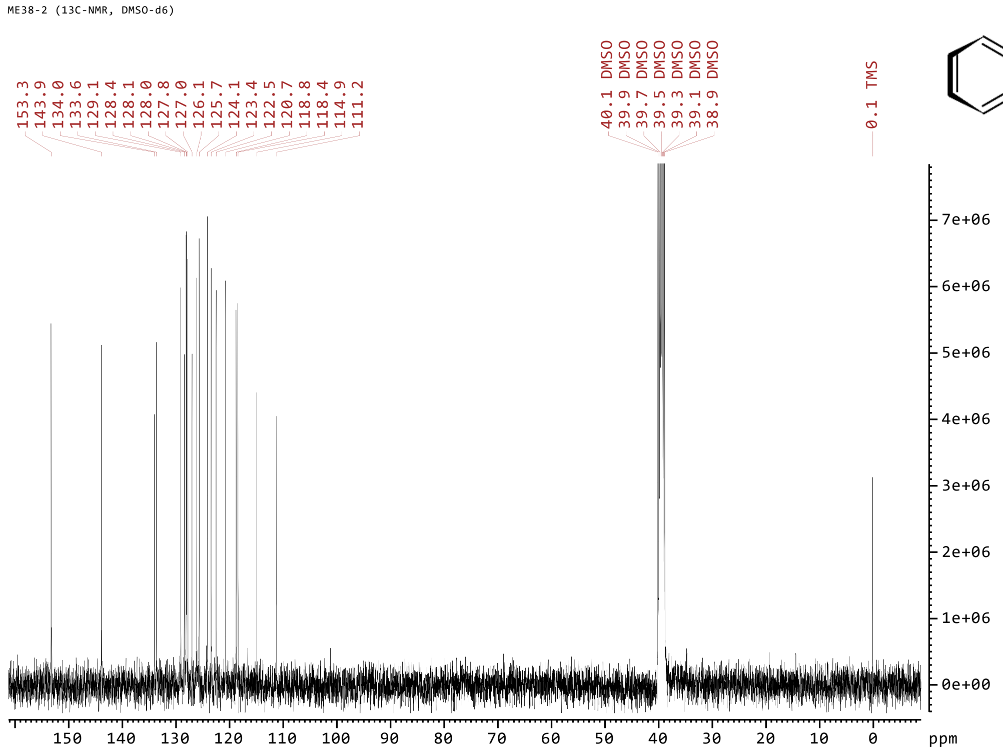

Compound 11(*S*) - ^{1}H-NMR (DMSO-*d*6)

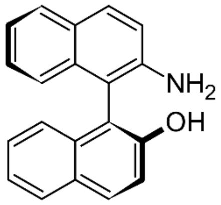

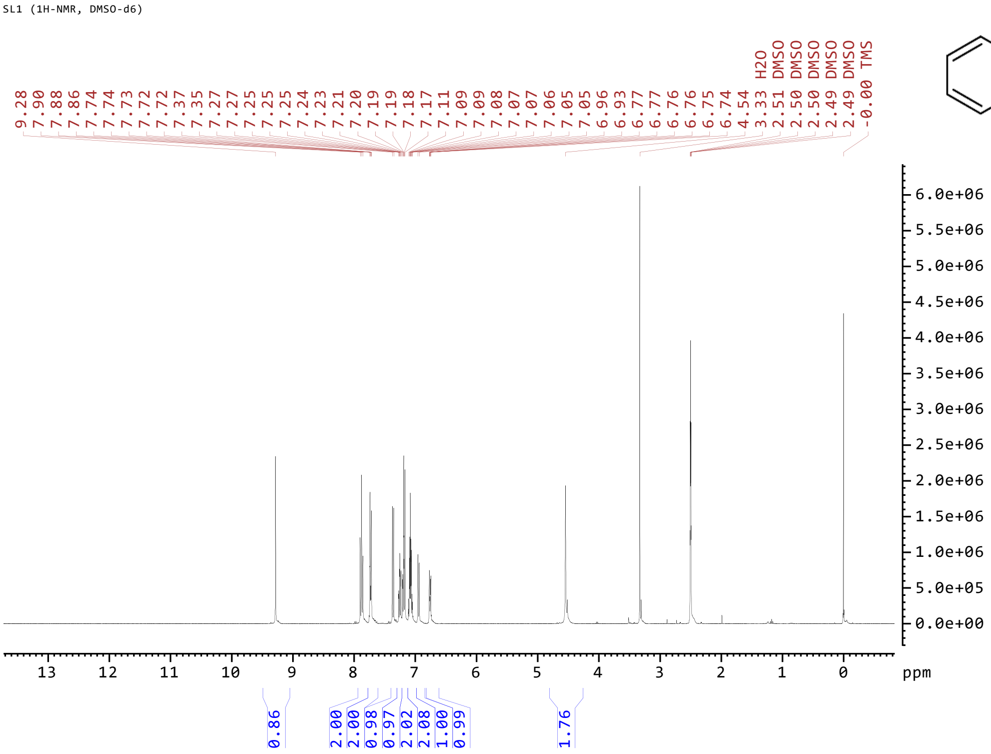

Compound 11(*S*) - ^{13}C-NMR (DMSO-*d*6)

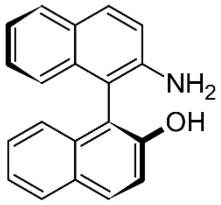

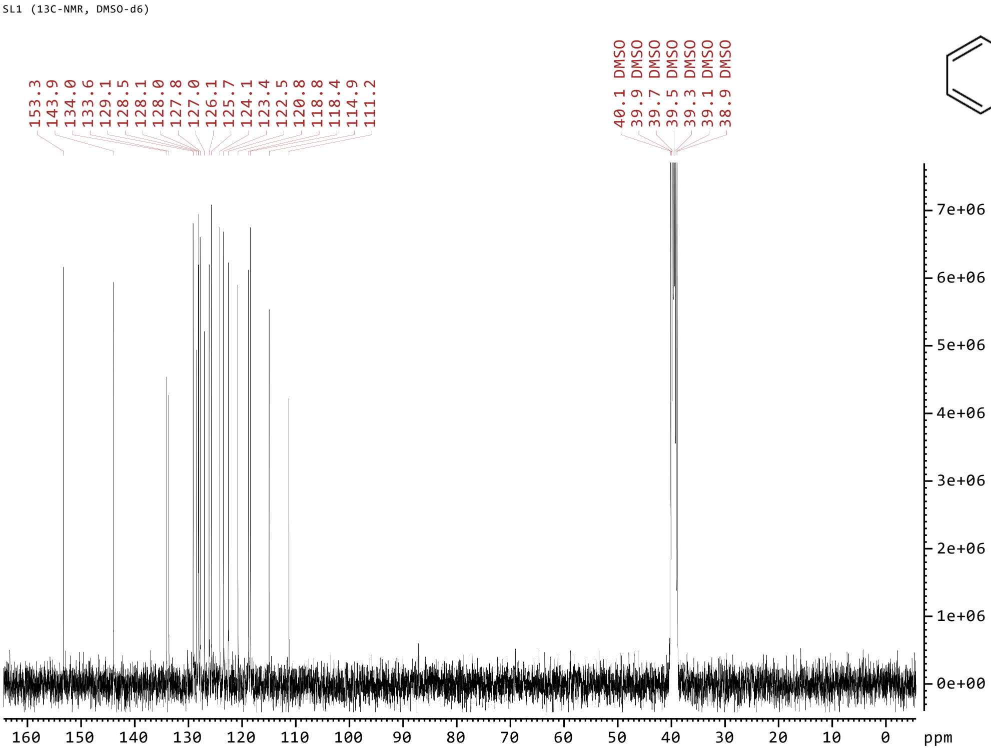

Compound 12(*R*) - ^{1}H-NMR (DMSO-*d6*)

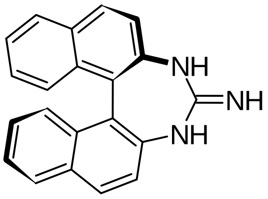

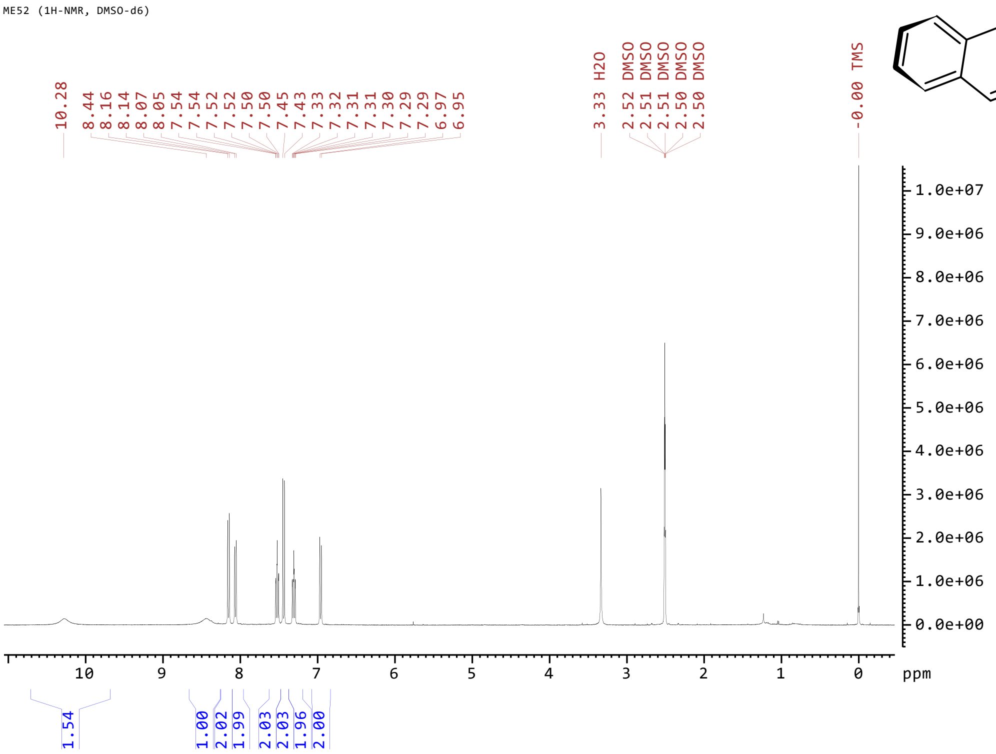

Compound 12(*R*) - ^{13}C-NMR (DMSO-*d6*)

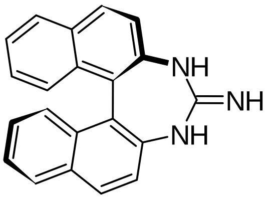

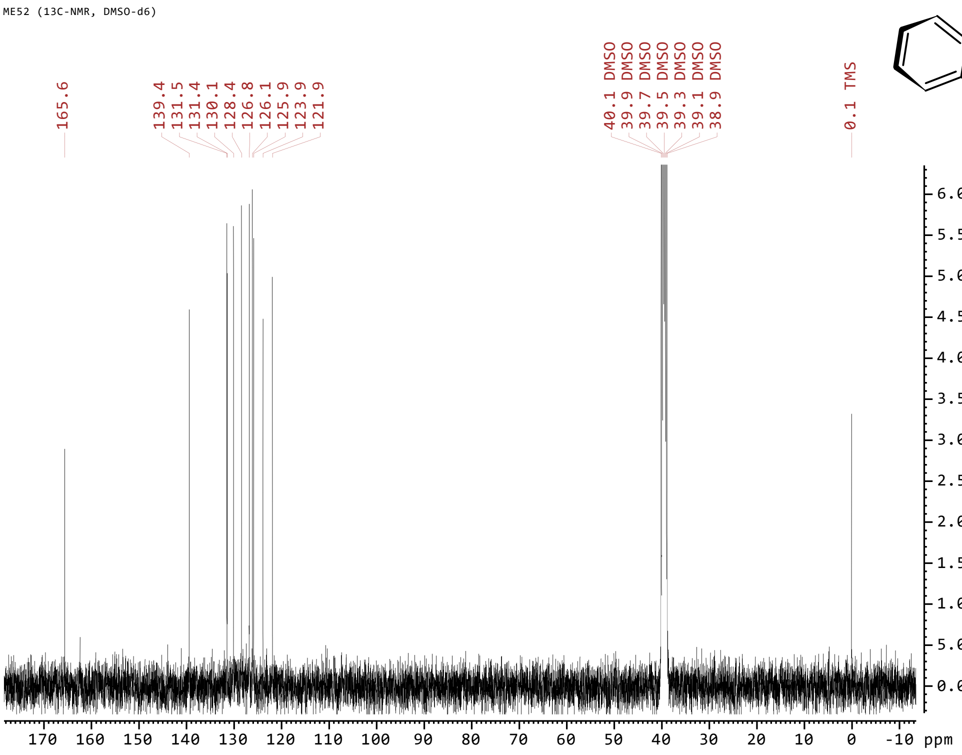

Compound 12(*S*) - ^{1}H-NMR (DMSO-*d6*)

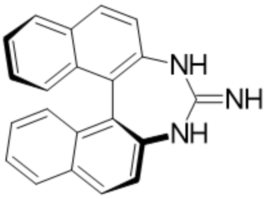

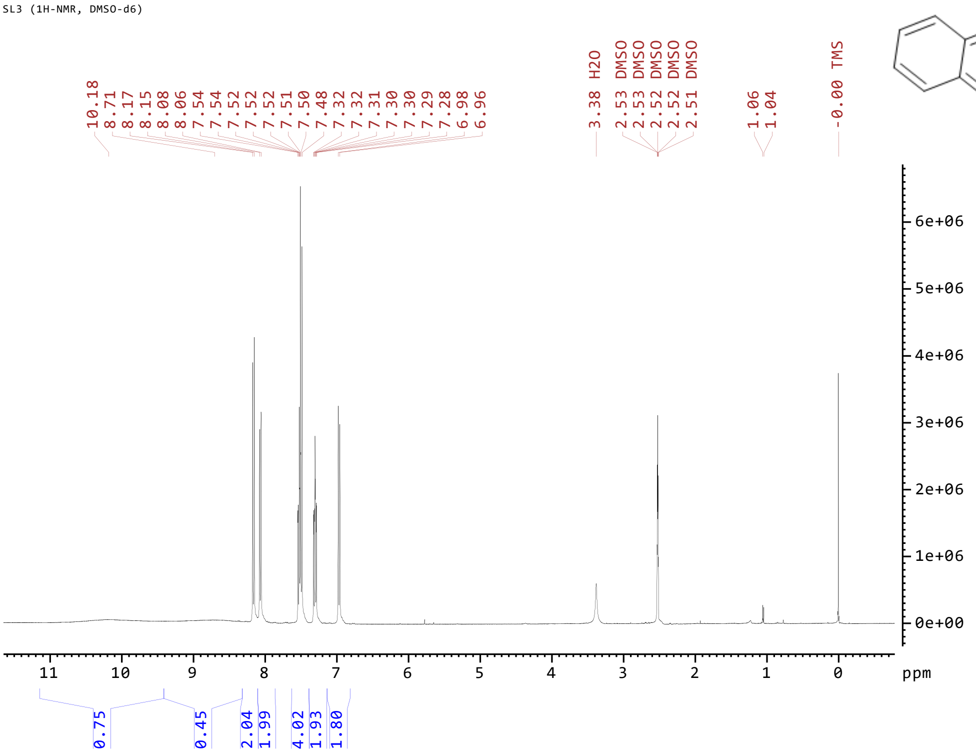

Compound 12(*S*) - ^{13}C-NMR (DMSO-*d6*)

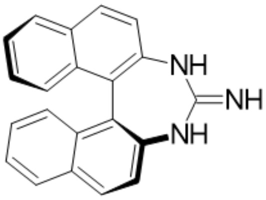

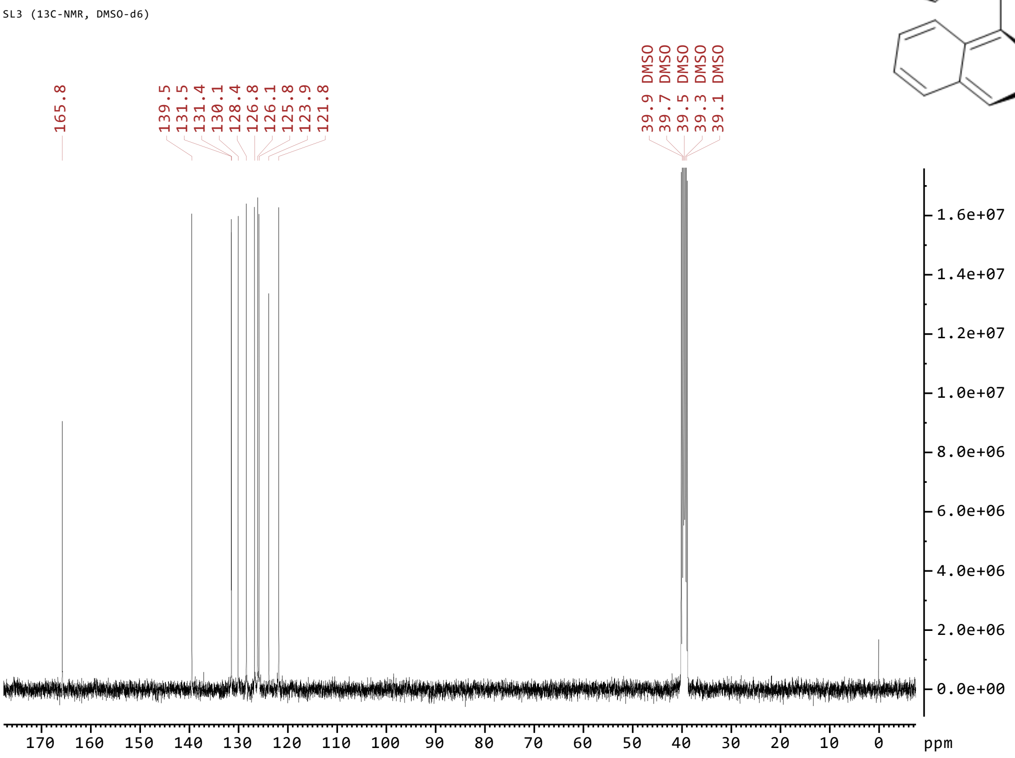

Compound 14(*R*) - ^{1}H-NMR (DMSO-*d6*)

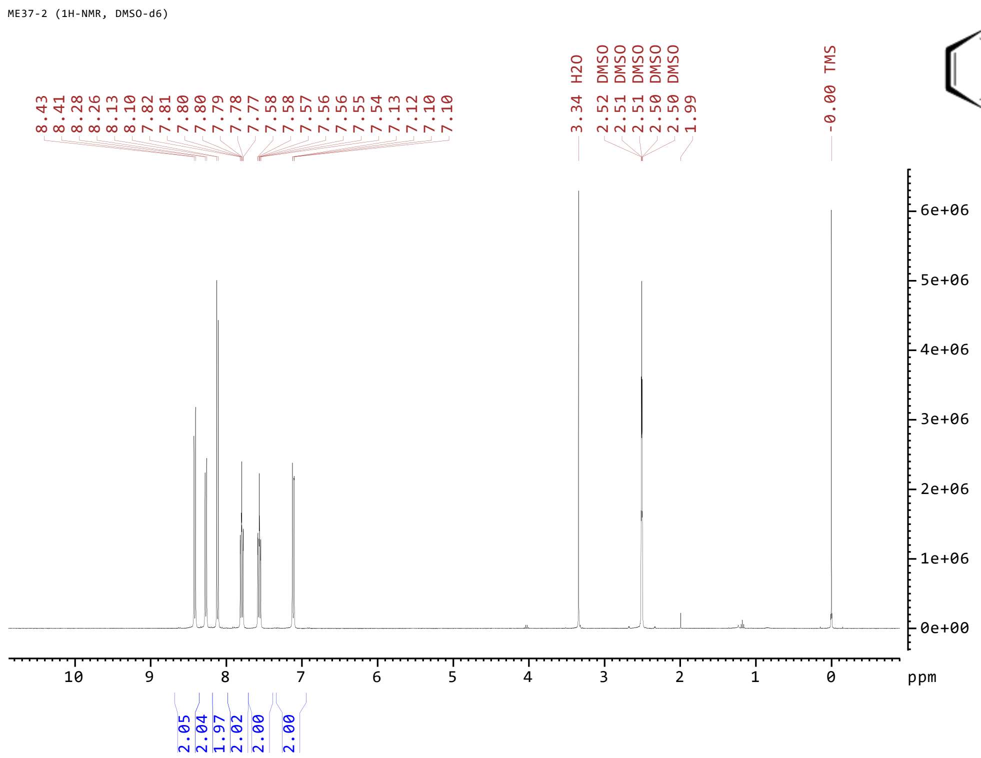

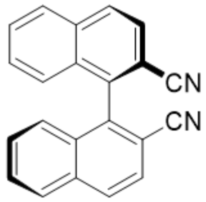

Compound 14(*R*) - ^{13}C-NMR (DMSO-*d6*)

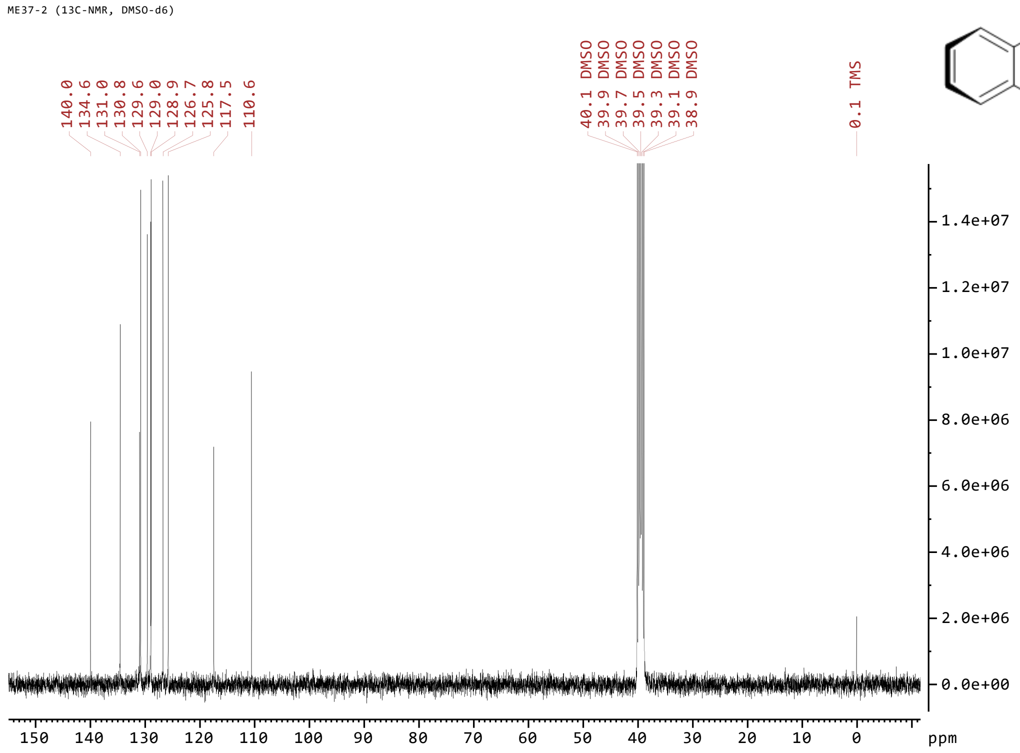

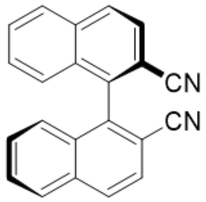

Compound 14(*S*) - ^{1}H-NMR (DMSO-*d*6)

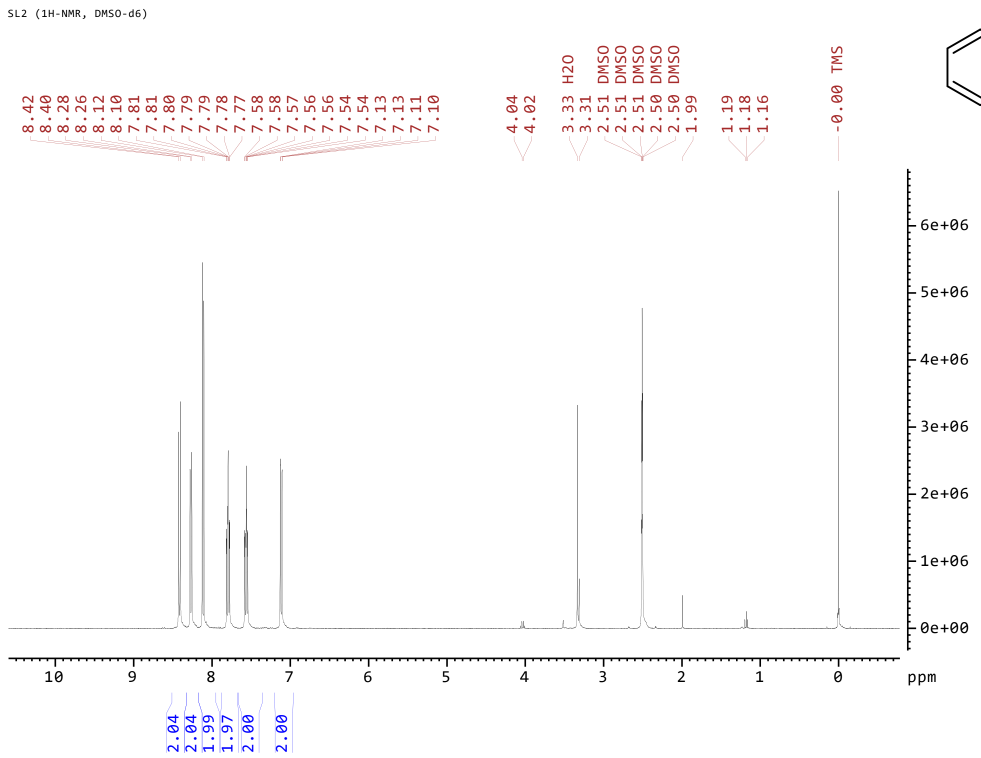

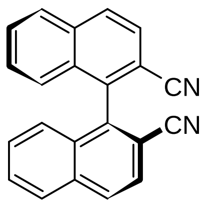

Compound 14(*S*) - ^{13}C-NMR (DMSO-*d*6)

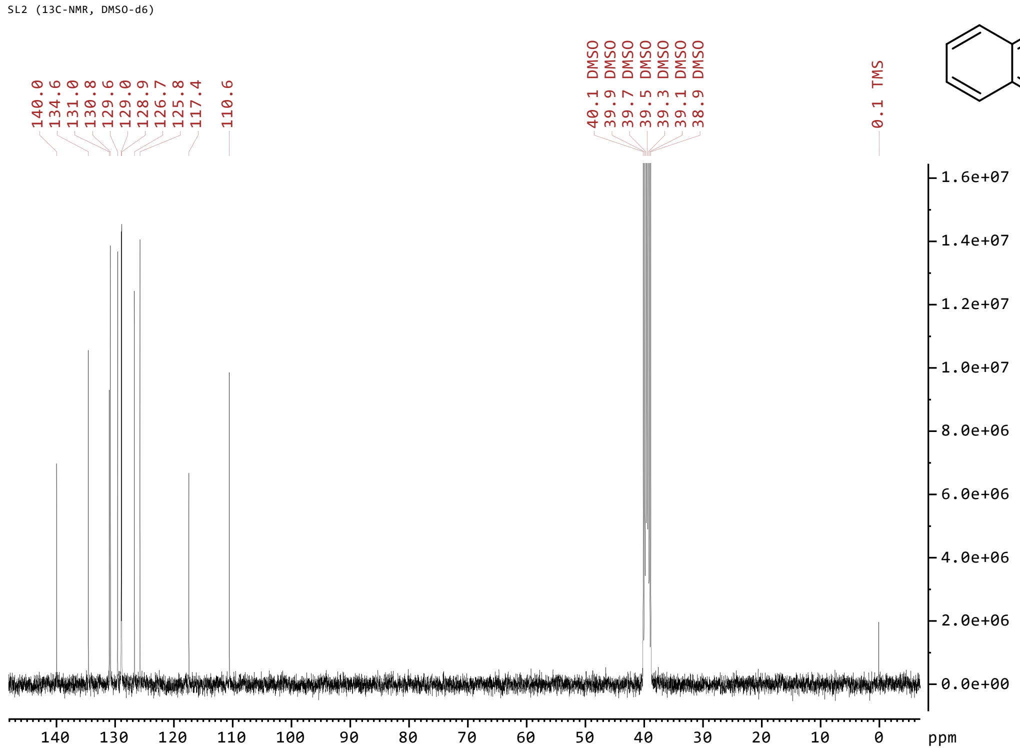

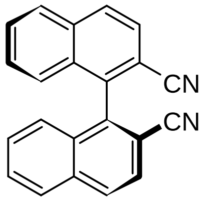

Compound 15(*R*) - ^{1}H-NMR (DMSO-*d6*)

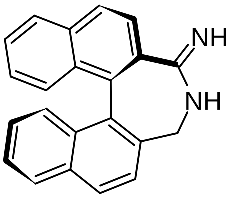

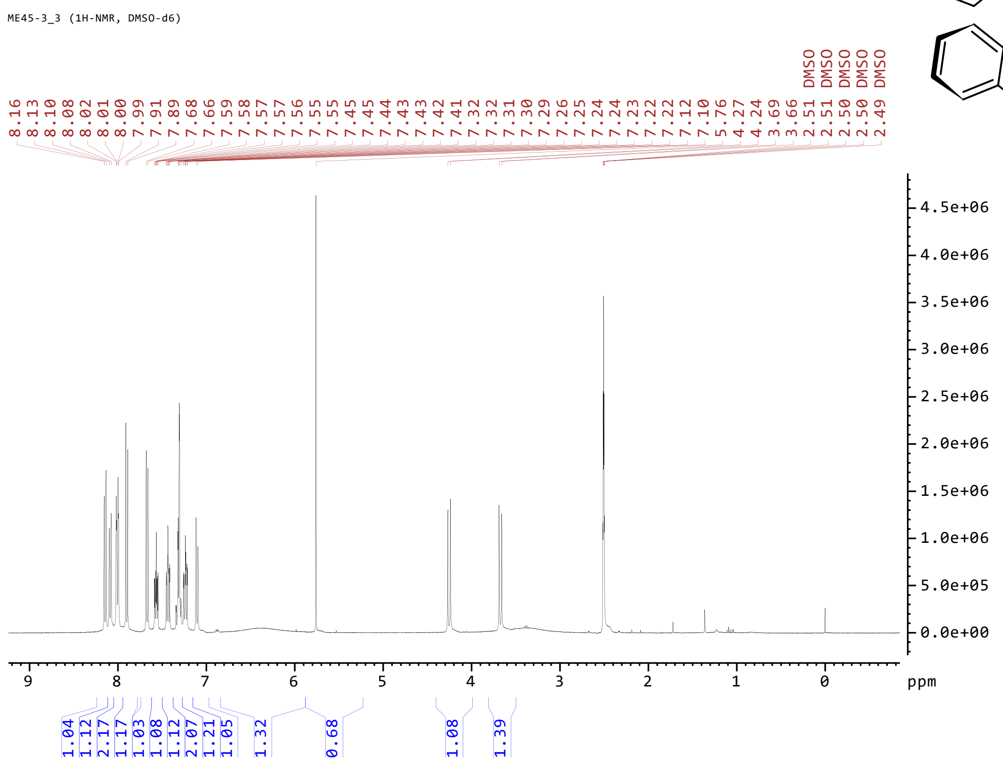

Compound 15(*R*) - ^{13}C-NMR (DMSO-*d6*)

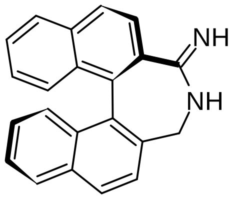

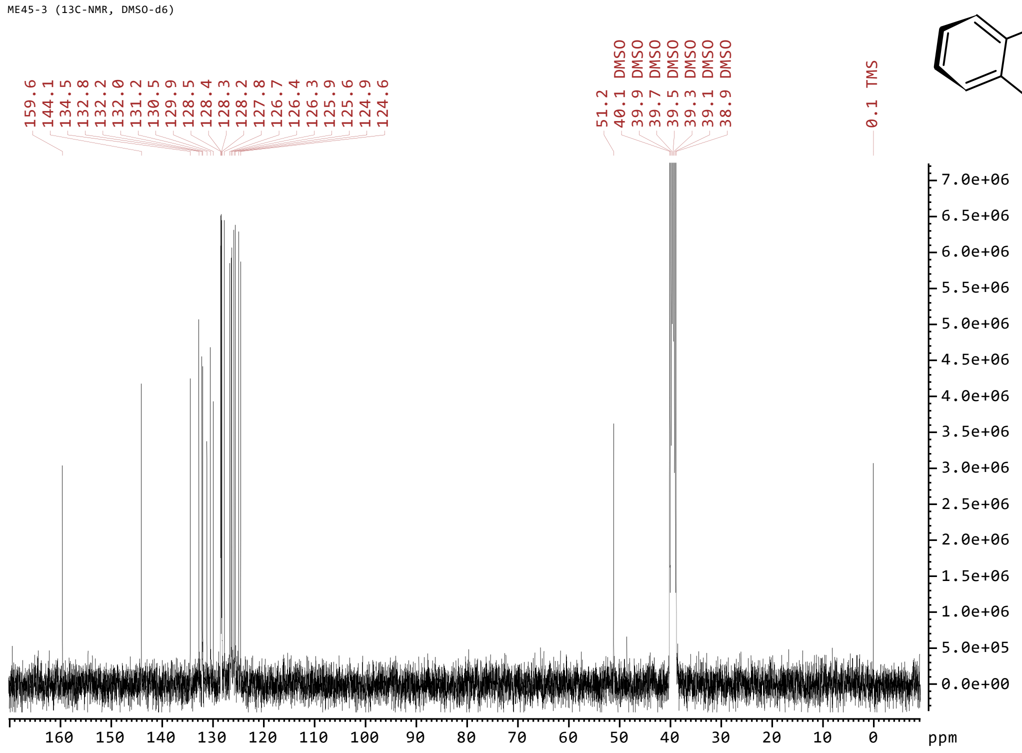

Compound 15(*S*) - ^{1}H-NMR (DMSO-*d6*)

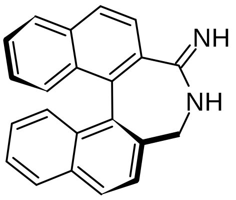

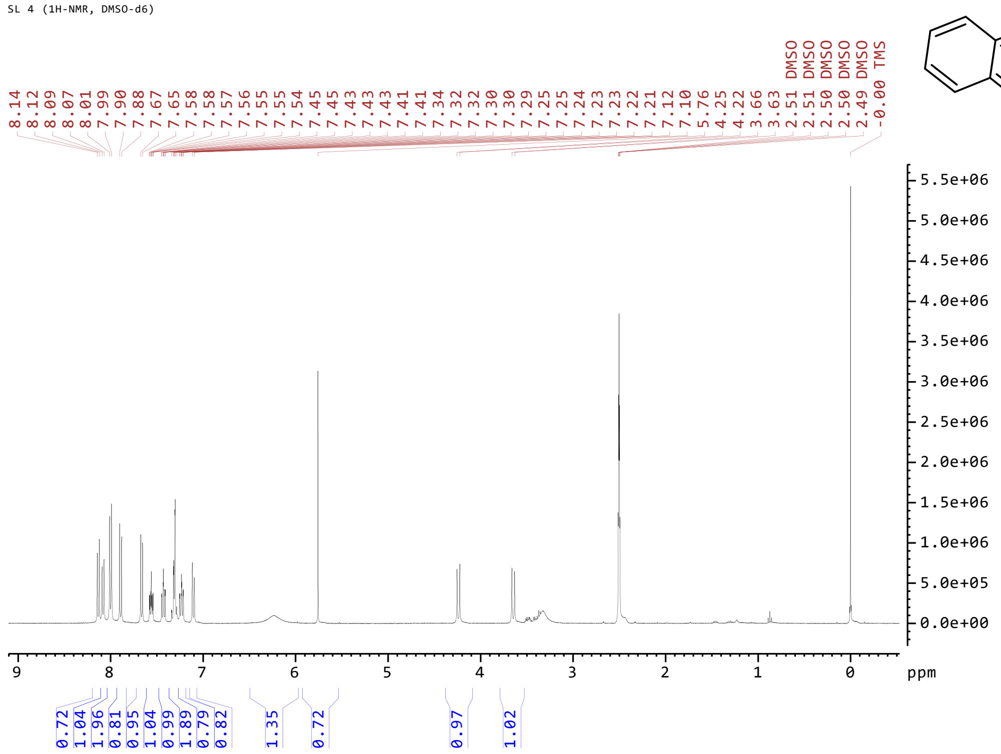

Compound 15(*S*) - ^{13}C-NMR (DMSO-*d6*)

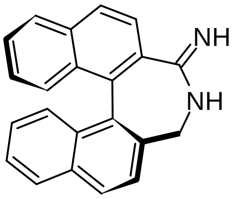

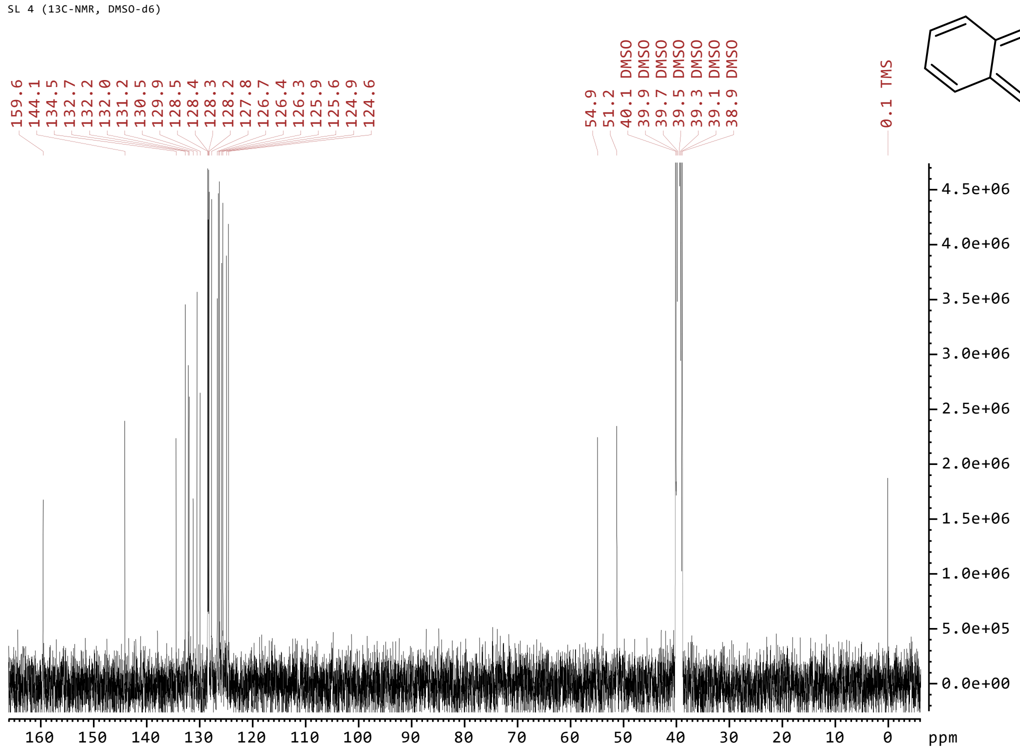

Compound 16(*R*) - ^{1}H-NMR (DMSO-*d6*)

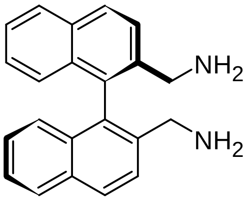

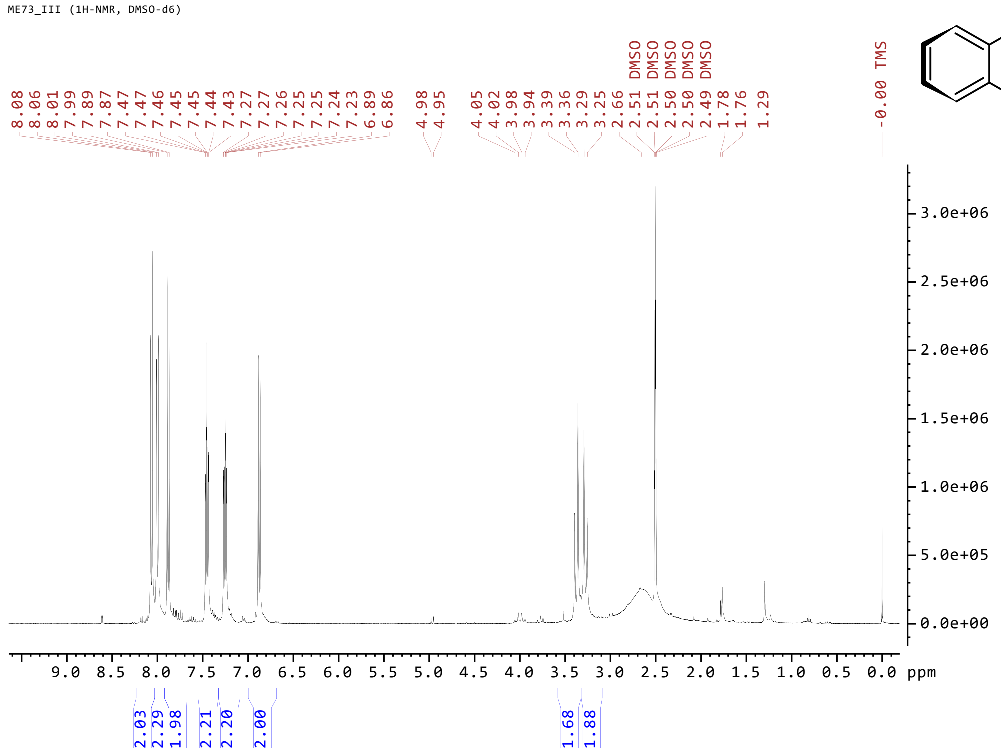

Compound 16(*R*) - ^{13}C-NMR (DMSO-*d6*)

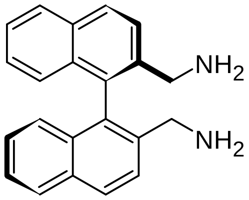

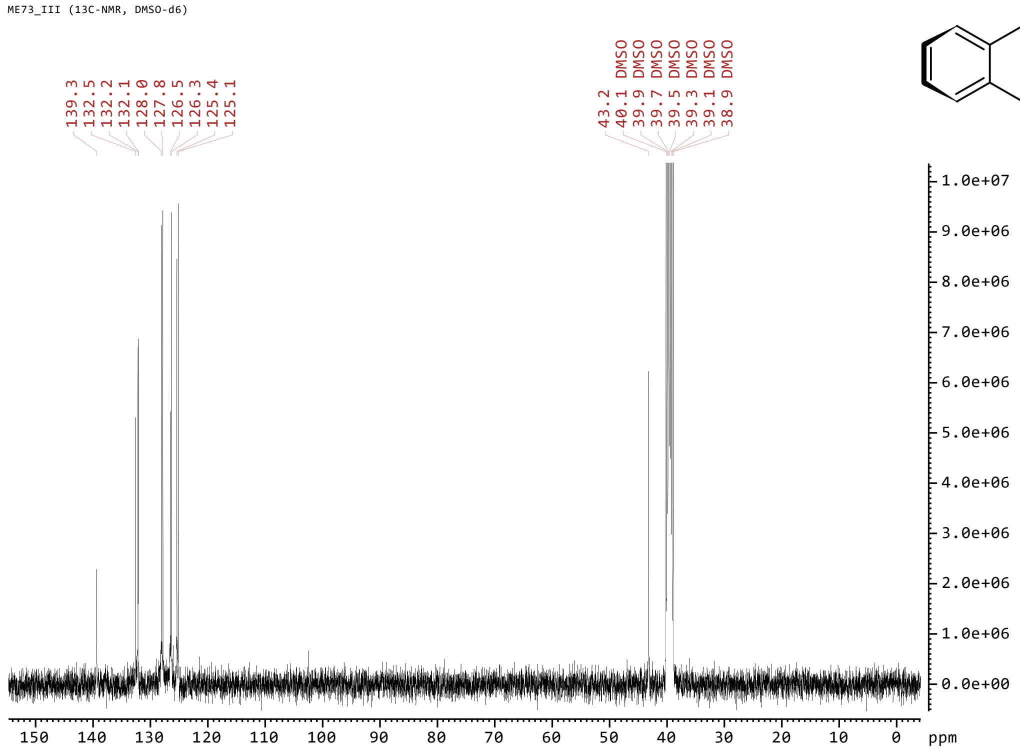

Compound 16(*S*) - ^{1}H-NMR (DMSO-*d6*)

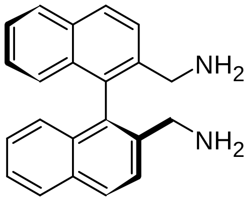

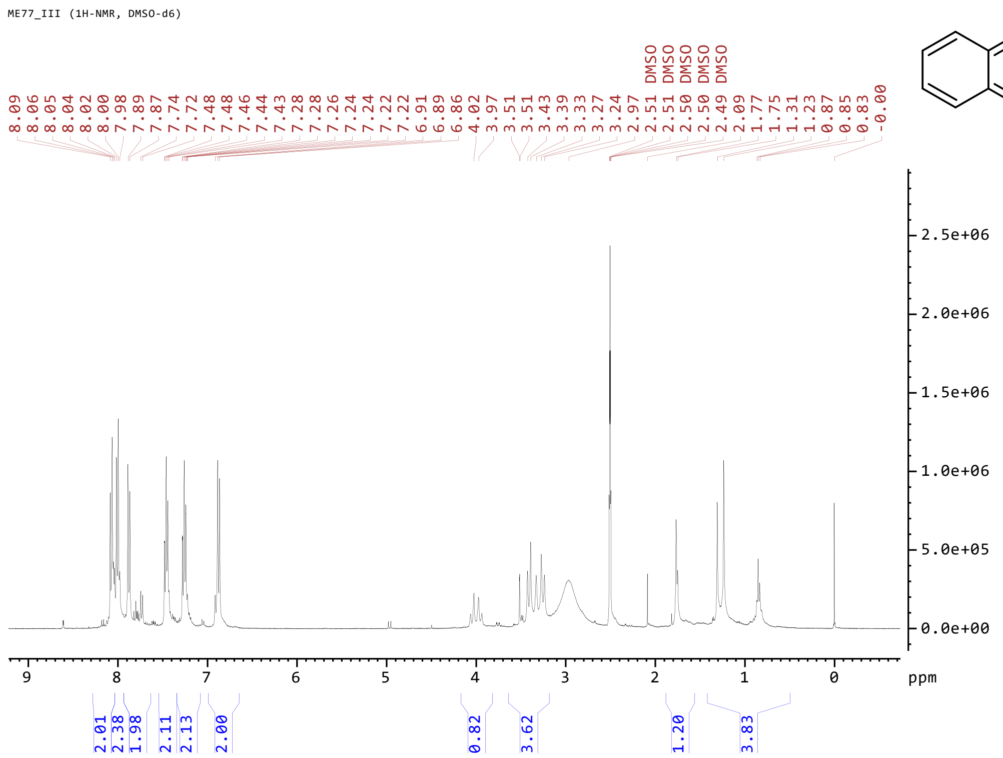

Compound 16(*S*) - ^{13}C-NMR (C*D*Cl3)

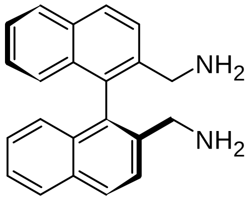

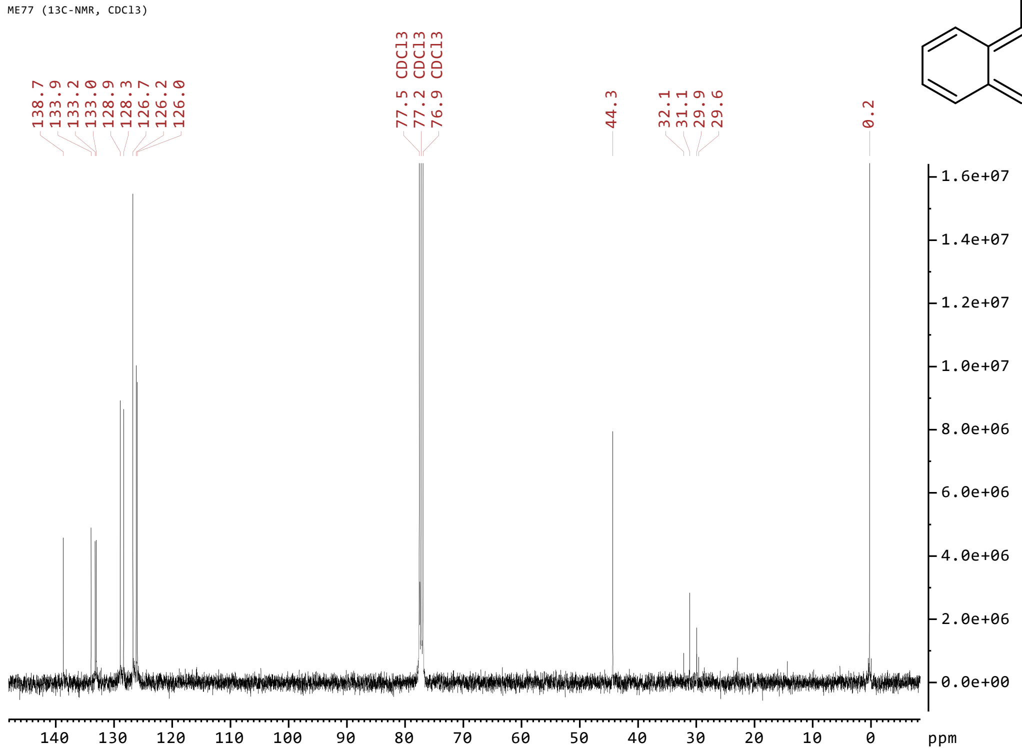

Compound 17 - ^{1}H-NMR (DMSO-*d6*)

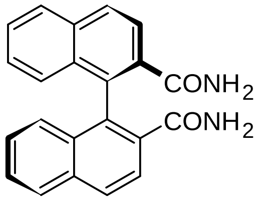

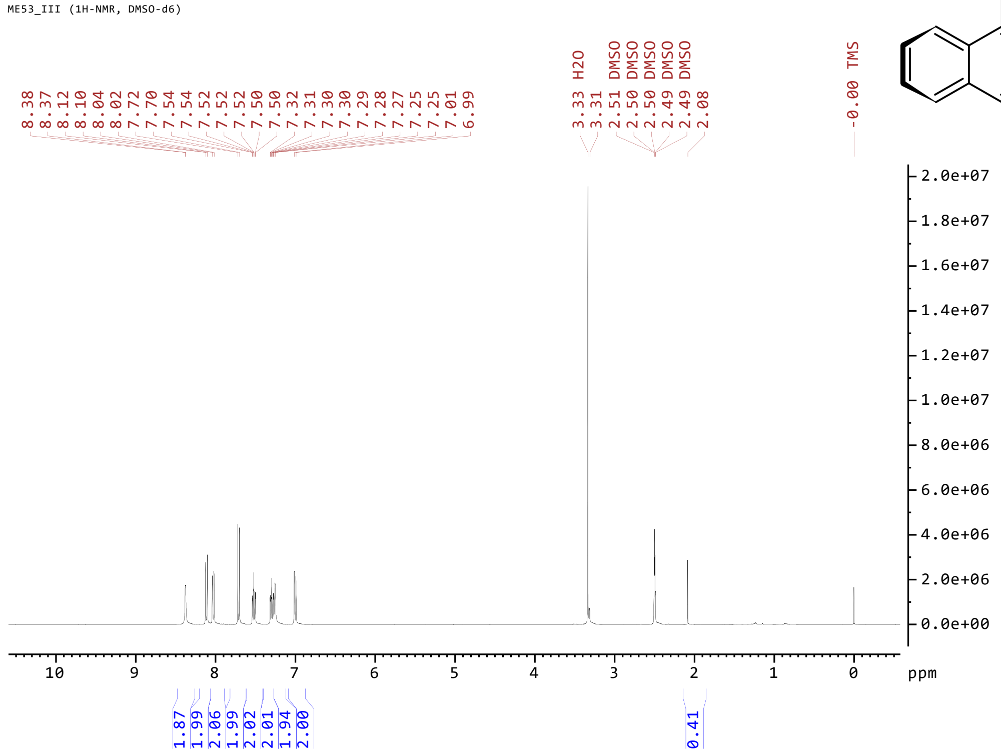

Compound 17 - ^{13}C-NMR (DMSO-*d6*)

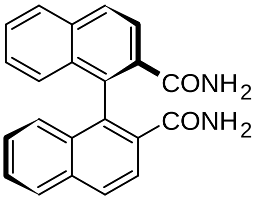

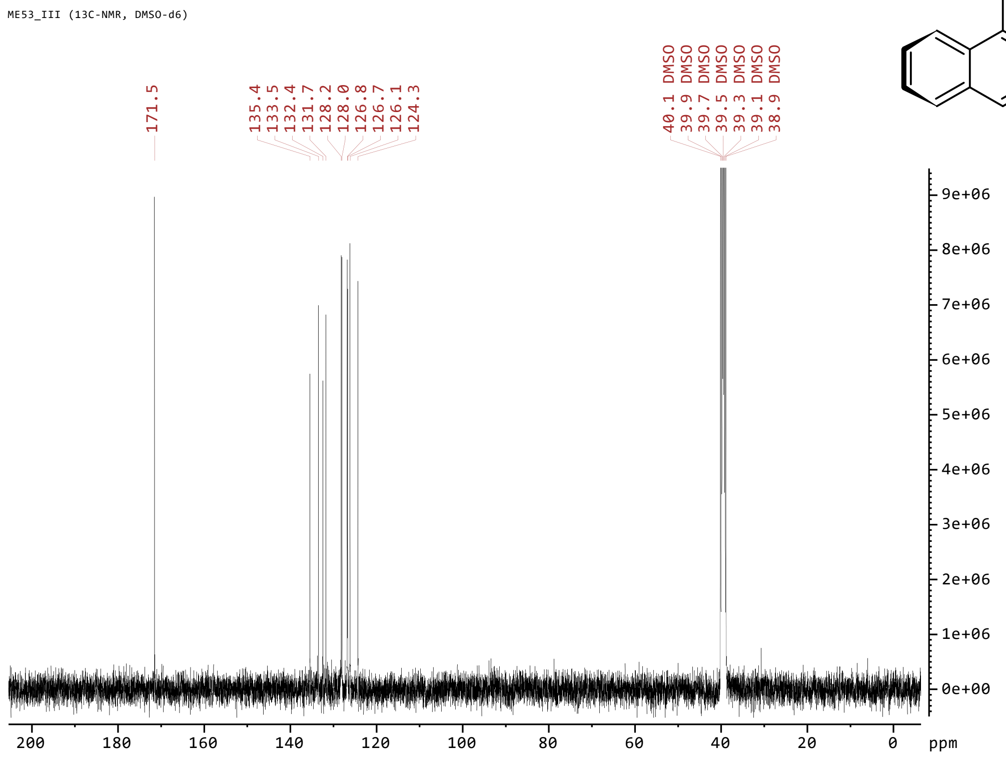

Supplement: Supplementary file 1 — Supplementary Material [file CMDC-20-e202500426-s001.pdf]
